# European Heart Rhythm Association (EHRA)/Heart Rhythm Society (HRS)/Asia Pacific Heart Rhythm Society (APHRS)/Latin American Heart Rhythm Society (LAHRS) expert consensus on risk assessment in cardiac arrhythmias: use the right tool for the right outcome, in the right population

**DOI:** 10.1002/joa3.12338

**Published:** 2020-06-15

**Authors:** Jens Cosedis Nielsen, Yenn‐Jiang Lin, Marcio Jansen de Oliveira Figueiredo, Alireza Sepehri Shamloo, Alberto Alfie, Serge Boveda, Nikolaos Dagres, Dario Di Toro, Lee L. Eckhardt, Kenneth Ellenbogen, Carina Hardy, Takanori Ikeda, Aparna Jaswal, Elizabeth Kaufman, Andrew Krahn, Kengo Kusano, Valentina Kutyifa, Han S. Lim, Gregory Y. H. Lip, Santiago Nava‐Townsend, Hui‐Nam Pak, Gerardo Rodríguez Diez, William Sauer, Anil Saxena, Jesper Hastrup Svendsen, Diego Vanegas, Marmar Vaseghi, Arthur Wilde, T. Jared Bunch, Alfred E. Buxton, Gonzalo Calvimontes, Tze‐Fan Chao, Lars Eckardt, Heidi Estner, Anne M. Gillis, Rodrigo Isa, Josef Kautzner, Philippe Maury, Joshua D. Moss, Gi‐Byung Nam, Brian Olshansky, Luis Fernando Pava Molano, Mauricio Pimentel, Mukund Prabhu, Wendy S. Tzou, Philipp Sommer, Janice Swampillai, Alejandro Vidal, Thomas Deneke, Gerhard Hindricks, Christophe Leclercq

**Affiliations:** ^1^ Department of Cardiology Aarhus University Hospital Skejby Denmark; ^2^ Division of Cardiology Department of Medicine Taipei Veterans General Hospital Taipei Taiwan; ^3^ Electrophysiology Service Department of Internal Medicine University of Campinas Hospital Campinas Brazil; ^4^ Department of Electrophysiology Leipzig Heart Center at University of Leipzig Leipzig Germany; ^5^ Division of Electrophysiology Instituto Cardiovascular Adventista Clinica Bazterrica Buenos Aires Argentina; ^6^ Department of Cardiology Clinique Pasteur Toulouse France; ^7^ Division of Electrophysiology Department of Cardiology Argerich Hospital and CEMIC Buenos Aires Argentina; ^8^ Department of Medicine University of Wisconsin‐Madison Madison WI USA; ^9^ Division of Cardiology Virginia Commonwealth University School of Medicine Richmond USA; ^10^ Heart Institute University of São Paulo Medical School Arrhythmia Unit Instituto do Coração ‐InCor‐ Faculdade de Medicina de São Paulo São Paulo Brazil; ^11^ Faculty of Medicine Department of Cardiovascular Medicine Toho University Japan; ^12^ Department of Cardiac Electrophysiology Fortis Escorts Heart Institute New Delhi India; ^13^ The Heart and Vascular Research Center Metrohealth Campus of Case Western Reserve University Cleveland OH USA; ^14^ Division of Cardiology Department of Medicine University of British Columbia Vancouver Canada; ^15^ Division of Arrthythmia and Electrophysiology Department of Cardiovascular Medicine National Cerebral and Cardiovascular Center Osaka Japan; ^16^ University of Rochester Medical Center Rochester USA; ^17^ Heart and Vascular Center Semmelweis University Budapest Hungary; ^18^ Department of Cardiology Austin Health Melbourne VIC Australia; ^19^ Cardiovascular Medicine University of Melbourne Melbourne VIC Australia; ^20^ Liverpool Centre for Cardiovascular Science University of Liverpool and Liverpool Heart & Chest Hospital Liverpool UK; ^21^ Aalborg Thrombosis Research Unit Department of Clinical Medicine Aalborg University Aalborg Denmark; ^22^ Department of Electrocardiology National Institute of Cardiology “Ignacio Chavez” Mexico City Mexico; ^23^ From the Division of Cardiology Department of Internal Medicine Yonsei University Health System Seoul Republic of Korea; ^24^ Department of Electrophysiology and Hemodynamic Arrhytmias Unity CMN 20 de Noviembre ISSSTE Mexico City Mexico; ^25^ Cardiovascular Division Brigham and Women’s Hospital and Harvard Medical School Boston USA; ^26^ Department of Cardio Electrophysiology Fortis Escorts Heart Institute New Delhi India; ^27^ Department of Cardiology, Rigshospitalet University of Copenhagen Copenhagen Denmark; ^28^ Amsterdam UMC University of Amsterdam Heart Center Department of Clinical and Experimental Cardiology Amsterdam The Netherlands; ^29^ Hospital Militar Central Bogotá Colombia; ^30^ UCLA Cardiac Arrhythmia Center UCLA Health System David Geffen School of Medicine, at UCLA Los Angeles USA; ^31^ Heart Center Department of Clinical and Experimental Cardiology Amsterdam UMC University of Amsterdam Amsterdam The Netherlands; ^32^ Department of Medicine Intermountain Heart Institute Intermountain Medical Center Salt Lake City USA

## TABLE OF CONTENTS

**Table:** 

1 INTRODUCTION	5
1.1. Evidence review	5
1.2 Relationships with industry and other conflicts	6
2. GENERAL TOOLS FOR RISK ASSESSMENT, STRENGTHS, LIMITATIONS, AND PRETEST PROBABILITY	7
2.1 Value of clinical history and characteristics including clinical risk scores such as CHA_2_DS_2_‐VASc	7
2.2 Electrocardiographic methods including monitoring	9
2.2.1 Electrocardiographic methods	9
2.2.2 P wave and PR interval	9
2.2.3 QRS, QT interval, and T‐wave	10
2.2.4 Ambulatory electrocardiogram monitoring	12
2.3 Imaging	13
2.3.1 Risk assessment of ventricular tachyarrhythmia using imaging modalities	13
2.3.2 Imaging modalities for atrial arrhythmias	14
2.4 Invasive electrophysiological study	15
2.5 Implantable loop recorders	17
2.5.1 Implantable loop recorder to diagnose unexplained syncope/atrial fibrillation with cryptogenic stroke	17
2.5.2 Implantable loop recorder to diagnose atrial and ventricular arrhythmia events	17
2.6 Wearables/direct to consumer	19
2.7 Biomarkers, tissue, genetics	21
2.7.1 Biomarkers	21
2.7.2 Tissue diagnostics	21
2.7.3 Genetics	22
2.8 Artificial intelligence	23
3 HOW TO ASSESS RISK FOR ATRIAL FIBRILLATION IN SPECIFIC POPULATIONS	24
3.1 Patients of advanced age	24
3.2 Patients with heart failure	26
3.2.1 Clinical risk factors	26
3.2.2 Electrocardiography	27
3.2.3 Biomarkers	27
3.2.4 Imaging	27
3.2.5 Genetics	27
3.3 Patients with obesity, hypertension, diabetes, sleep apnoea or structural heart disease	29
3.4 Patients who have undergone cardiac surgery	31
3.5 Patients with cryptogenic stroke	32
3.6 How to assess high risk of atrial fibrillation in professional athletes	34
3.6.1 Atrial fibrillation risk in athletes—general	34
3.6.2 Atrial fibrillation risk in athletes—exercise paradox	34
3.6.3 Atrial fibrillation risk in athletes—structural cardiac changes	34
3.7 Patients with inherited rhythm disease (long QT syndrome/short QT syndrome/catecholaminergic polymorphic ventricular tachyarrhythmia/Brugada syndrome)	36
4 HOW TO ASSESS RISK FOR ADVERSE OUTCOMES IN PATIENTS WITH ATRIAL FIBRILLATION	**38**
4.1 Risk assessment for stroke/transient ischaemic attack/cognitive decline	38
4.2 Risk assessment for stroke/transient ischaemic attack status post‐left atrial appendage occlusion/ligation	40
4.3 Risk for heart failure incidence and progression	41
4.4 Risk for death in atrial fibrillation patients	43
4.5 Risk of adverse outcomes in patients treated with catheter ablation	46
4.5.1 Post‐ablation atrial fibrillation recurrence	46
4.5.2 Other adverse outcomes	46
4.5.3 Catheter ablation in Wolff–Parkinson–White patients	47
4.6 Risk of adverse outcomes in patients treated with surgical Maze	48
4.6.1 Atrial fibrillation surgery	48
4.6.2 Surgical Maze in patients with concomitant heart surgery	48
4.6.3 Stand‐alone surgical Maze	48
4.6.4 Left atrial appendage exclusion or removal during surgical Maze	48
5 HOW TO ASSESS RISK FOR VENTRICULAR TACHYARRHYTHMIA IN SPECIFIC POPULATIONS	
5.1 Patients with ischaemic heart disease	**50**
5.1.1 Secondary prevention of ventricular tachyarrhythmia/ventricular fibrillation in patients with ICM	50
5.1.2 Primary prevention of ventricular tachyarrhythmia/ventricular fibrillation in patients with ICM and a left ventricular ejection fraction ≤35%	50
5.1.3 Primary prevention of ventricular tachyarrhythmia/ventricular fibrillation in patients with ICM and left ventricular ejection fraction >35%	51
5.2 Patients with non‐ischaemic heart failure	53
5.3 Patients with inflammatory cardiomyopathies	54
5.4 Patients with congenital heart disease	56
5.5 Patients with inherited arrhythmia diseases (Inherited channelopathies and inherited structural diseases including arrhythmogenic right ventricular cardiomyopathy)	58
5.6 Risk stratification in patients with arrhythmogenic cardiomyopathy, specified for arrhythmogenic right ventricular cardiomyopathy	60
5.7 Patients with Chagas disease	62
6. HOW TO ASSESS RISK FOR ADVERSE OUTCOMES IN PATIENTS WITH VENTRICULAR TACHYARRHYTHMIA	**63**
6.1 Risk for appropriate and inappropriate implantable cardioverter‐defibrillator therapies	63
6.1.1 Appropriate shock predictors	63
6.1.2 Inappropriate shock predictors	63
6.2 Risk for heart failure incidence and progression	64
6.3 Risk for death in ventricular tachyarrhythmia patients	66
6.4 Risk of adverse outcomes in patients treated with catheter ablation	68
7 HOW TO ASSESS RISK FOR ADVERSE OUTCOME IN Patients With Other Specific Cardiac Conditions	**69**
7.1 Patients with ventricular premature contractions	70
7.1.1 Premature ventricular complex frequency	70
7.1.2 Premature ventricular complex morphology	70
7.1.3 Premature ventricular complex coupling interval	70
7.2 Patients with supraventricular tachyarrhythmia such as Wolff–Parkinson–White syndrome and focal atrial tachycardia	72
SUMMARY	74
REFERENCES	75

## 1 INTRODUCTION

Patients with cardiac diseases or conditions with high risk of developing cardiac diseases undergo risk assessment by cardiologists, primary care physicians, and scientists based on referral for more advanced risk assessment strategies, institution of preventive treatments, counselling of patients and their relatives, and selection of patients for scientific trials. The various methods used for risk assessment differ with respect to availability, complexity, and usefulness in different patient populations. Parameters associated with increased risk of e.g. death may also be associated with higher risk of other adverse outcomes. However, risk assessment strategies including specific methods for risk assessment and risk scores should be used only for the purposes for which they are validated.

This expert consensus statement of the European Heart Rhythm Association (EHRA), Heart Rhythm Society (HRS), Asia Pacific Heart Rhythm Society (APHRS), and the Latin American Heart Rhythm Society (LAHRS) summarizes the consensus of the international writing group based on a thorough review of the medical literature regarding risk assessment in cardiac arrhythmias. To create a tool for clinicians to perform rational and evidence‐based risk stratification, this task force was set down by EHRA, HRS, LAHRS, and APHRS, including representatives from each of the four societies.

With this document, we intend to describe and review status of performing risk assessment in different patient populations with cardiac diseases or conditions with high risk of developing such. Our objectives are to raise awareness of using the right risk assessment tool for a given outcome in a given population, and to provide physicians with practical proposals that may lead to improvement of patient care in this regard. For quick reference, sub‐chapters start with a short section on consensus statements. The document concludes with a summary of consensus statements.

### 1.1 Evidence review

Members of the Task Force were asked to perform a detailed literature review using PubMed and EMBASE, weigh the strength of evidence for or against a particular treatment or procedure, and include estimates of expected health outcomes for which data exist. Patient‐specific modifiers, comorbidities, and issues of patient preference that might influence the choice of particular tests are considered, as are frequency of follow‐up and cost‐effectiveness. In controversial areas, or with regard to issues without evidence other than usual clinical practice, consensus was achieved by agreement of the expert panel after thorough deliberations. This document was prepared by the Task Force and peer‐reviewed by official external reviewers representing EHRA, HRS, APHRS, and LAHRS.

Consensus statements are evidence‐based and derived primarily from published data or determined through consensus opinion if no data available. Current systems of ranking level of evidence are becoming complicated in a way that might compromise their practical utility.^1^ In contrast to guidelines, we opted for an easier user‐friendly system of ranking using ‘coloured hearts’ that should allow physicians to easily assess the current status of the evidence and consequent guidance (Table 1). This EHRA grading of consensus statements does not have separate definitions of the level of evidence. The categorization used for consensus statements must not be considered directly similar to the one used for official society guideline recommendations which apply a classification (Class I‐III) and level of evidence (A, B, and C) to recommendations used in official guidelines.

**Table 1:** Scientific rationale of consensus statements

Definitions related to a treatment or procedure	Consensus statement instruction	Symbol
Scientific evidence that a treatment or procedure is beneficial and effective. Requires at least one randomized trial, or is supported by strong observational evidence and authors’ consensus (as indicated by an asterisk).	‘Should do this’	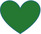
General agreement and/or scientific evidence favour the usefulness/efficacy of a treatment or procedure. May be supported by randomized trials based on a small number of patients or not widely applicable.	‘May do this’	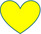
Scientific evidence or general agreement not to use or recommend a treatment or procedure.	‘Do not do this’	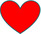

The categorization for our consensus document should not be considered directly similar to the one used for official society guideline recommendations which apply a classification (I‐III) and level of evidence (A, B, and C) to recommendations.

Thus, a green heart indicates a ‘should do this’ consensus statement or indicated risk assessment strategy based on at least one randomized trial or supported by strong observational evidence that it is beneficial and effective. A yellow heart indicates general agreement and/or scientific evidence favouring a ‘may do this’ statement or the usefulness/efficacy of a risk assessment strategy or procedure. A ‘yellow heart’ symbol may be supported by randomized trials based on a small number of patients or not widely applicable. Risk assessment strategies for which there is scientific evidence of no benefit or potential harm and should not be used (‘do not do this’) are indicated by a red heart.

Finally, this consensus document includes evidence and expert opinions from several countries. The risk assessment approaches discussed may therefore include tests not approved by governmental regulatory agencies in all countries.

### 1.2 Relationships with industry and other conflicts

All members of the writing group, as well as reviewers, have disclosed any potential conflicts of interest. Details are available in Supporting Information online.

All consensus statements were voted upon by the writing committee independently and reached the predefined level of ≥75% consensus for inclusion in consensus statement tables. Each partner society officially reviewed the document, and all reviewer comments were addressed. Each partner society approved the final document and consensus statements.

## 2 GENERAL TOOLS FOR RISK ASSESSMENT, STRENGTHS, LIMITATIONS, AND PRETEST PROBABILITY

### 2.1 Value of clinical history and characteristics including clinical risk scores such as CHA_2_DS_2_‐VASc

Clinical assessment of the patient with cardiac arrhythmias starts with a good clinical history and basic investigations for an underlying aetiological factor for the arrhythmia or its associated complication(s). In addition, an assessment of the risks and benefits of any therapeutic intervention should be made, and appropriate management initiated.

Following on from clinical history and assessment, there is a proposal toward a more integrated and holistic approach to arrhythmia management, as evident in guidelines. Such an integrated approach requires multidisciplinary teams of healthcare professionals, patient involvement, access to treatment options, and decision‐support tools to optimize the patient journey. Many proposals have been made towards the operationalization of such an integrated approach to risk assessment and practical management in cardiac arrhythmias, which has been of varying complexity. As an example, the management of atrial fibrillation (AF) has been simplified into the ABC pathway (‘A’ Avoid stroke with Anticoagulation; ‘B’ Better symptom management, with patient‐centred and symptom‐directed decisions on rate or rhythm control; ‘C’ Cardiovascular and comorbidity risk management), which has been shown to be associated with improved clinical outcomes and reduced healthcare costs.^2^, ^3^, ^4^, ^5^, ^6^

This makes a strong argument for using the right approaches and clinical tools for patient assessment, but using them appropriately for the reasons they were first proposed (e.g. stroke risk scores to assess stroke risk, and not other outcomes).

Taking AF as an *illustrative example* with regard to using the right score for the right reason there are many risk factors for stroke (but the more common and validated ones have been used to formulate risk stratification tools).^7^ The most common in use is the CHA_2_DS_2_‐VASc score^8^ but it is not meant to include every possible stroke risk factor, and was designed to be simple, reductionist and practical to help decision‐making for stroke risk. As with all clinical scores based on clinical factors, the CHA_2_DS_2_‐VASc score only performs modestly for predicting high‐risk patients who sustain events. The use of more clinical factors and biomarkers improves prediction (at least statistically) but the practical added value is marginal, and less impressive in real‐world cohorts.^9^, ^10^ Use of simplified scores to artificially categorize patients into low‐, moderate‐ and high‐risk strata can be problematic, as in the real‐world patients do not necessarily fall into three neat categories of risk. Also, not all risk factors carry equal weight, hence, the move to focus the initial decision‐making on identifying low‐risk patients who do not need antithrombotic therapy first, following which stroke prevention can be offered to AF patients with ≥1 stroke risk factors.^9^ Stroke risk is also highly dynamic, and although logistically challenging, a clinical reassessment may be needed every 4‐6 months to optimize risk re‐assessment.^11^, ^12^, ^13^ As the CHA_2_DS_2_‐VASc is a cluster of common cardiovascular risk factors, it is predictive of death, cardiovascular hospitalizations, and other adverse outcomes that the CHA_2_DS_2_‐VASc score was not designed for. Also, given that many components of the CHA_2_DS_2_‐VASc score are associated with incident AF, the CHA_2_DS_2_‐VASc score is used to predict new onset AF, again something it was not designed for. Another misuse of the CHA_2_DS_2_‐VASc score is the prediction of bleeding risk. Nevertheless, formal comparisons show that the CHA_2_DS_2_‐VASc (and older CHA_2_DS_2_) score are inferior to a formal bleeding risk score such as the HAS‐BLED score, for the prediction of major bleeding in AF patients.^14^

Indeed, bleeding risk is also highly dynamic, and the appropriate use of bleeding risk scores such as HAS‐BLED is to address modifiable bleeding risk factors (e.g. uncontrolled hypertension, labile INR, concomitant aspirin, or NSAID use) then to schedule the ‘high risk’ patients for early and more frequent follow‐up visits (e.g. 4 weeks rather than 4 months).^15^ Only focusing on modifiable bleeding risk factors is an inferior strategy for bleeding risk assessment, compared to the HAS‐BLED score.^8^

We should use the scores only for the purposes they were designed for. Attention to appropriate methodology, statistics, etc.—as well as other clinical states merits consideration e.g. sudden death prediction (or failed ablation, device infection, etc.), Charlson Comorbidity Index, frailty etc.—but using the right score designed for that purpose.

If appropriately used, some of these (simplified) tools help with clinical management. Indeed, the value of a medical test is measured by its accuracy as well as how it impacts medical decisions and ultimately patient health. As medical tests are considered and new ones emerge, they should be considered and evaluated in a framework of accuracy and patient impact.^16^ A test must not only be accurate, but also feasible. Tests that are difficult to reproduce, subject to technical failures, or difficult to interpret are likely to impact patient care as a consequence of a primary failure to produce a definitive and actionable result.

### 2.2 Electrocardiographic methods including monitoring

**Table:** 

Electrocardiographic methods including monitoring	Class	References
Twelve‐lead electrocardiogram (ECG) should be obtained in all patients undergoing evaluation for known or suspected heart disease.	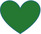	^17^
The 12‐lead ECG provides diagnostic and prognostic information in patients with inherited high‐risk syndromes including long QT syndrome (LQTS), short QT syndrome, Brugada Syndrome, and arrhythmogenic cardiomyopathy and should be obtained.	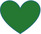	^17^
Exercise ECG provides diagnostic and prognostic information for patients with LQTS arrhythmogenic cardiomyopathy, hypertrophic cardiomyopathy (HCM), catecholaminergic polymorphic ventricular tachycardia, and documented or suspected arrhythmias related to exertion, and should be obtained.	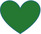	^17^
Ambulatory ECG evidence of non‐sustained ventricular tachycardia provides prognostic information in ischaemic cardiomyopathy, arrhythmogenic cardiomyopathy, and HCM and should be obtained.	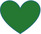	^17^
The signal‐averaged ECG and QRS fragmentation may aid in the diagnosis of arrhythmogenic cardiomyopathy.	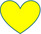	^18^
The signal‐averaged ECG and QRS fragmentation may be useful in risk stratification of Brugada syndrome.	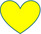	^18^
Heart rate variability, heart rate turbulence, signal‐averaged ECG, and T wave alternans analysis, when used in combination with additional clinical, electrocardiographic, and structural measures, may be useful for identifying high‐ and low‐risk groups among patients with acquired structural heart disease.	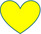	^19^

#### 2.2.1 Electrocardiographic methods

The ECG is the gold standard for risk assessment in patients with or at risk of developing cardiac arrhythmias. The 12‐lead ECG is inexpensive and widely available. Risk stratification with the ECG is limited in general by its low positive predictive value (PPV) determined to a large extent by the low prevalence of cardiovascular events in the general population. However, the prognostic significance of the ECG is enhanced in patients with heart disease.

#### 2.2.2 P wave and PR interval

The prognostic value of P wave characteristics has been examined in subjects enrolled in clinical trials of AF for prediction of the development of AF, where maximum P wave duration was a significant independent risk marker for the development of AF over 10 years.^20^ This observation was confirmed by epidemiologic/population studies (including ARIC and the Copenhagen ECG studies) that showed increased risk of AF in patients with prolonged P wave duration and PR interval prolongation,^21^, ^22^, ^23^ and summarized in a review by Nikolaidou et al*.* ^24^ Moreover, a prolonged P wave duration was determined as a sensitive predictor of post‐operative AF in patients undergoing coronary artery bypass grafting (CABG).^25^ The definition of an abnormal P wave varies greatly depending on how it is measured, and definitions vary depending on whether P wave area, duration, terminal forces in lead V1 or signal‐averaged P wave are analysed. Abnormal P wave morphology was associated with incident stroke in the Multi‐Ethnic Study of Atherosclerosis.^26^ The prognostic significance of PR interval prolongation, which is variably defined as PR intervals greater than 196‐220 milliseconds, is controversial and depends on the patient population studied. Most studies show that PR interval prolongation is not associated with increased mortality in healthy middle‐aged individuals during medium term follow‐up. On the other hand, a number of reports show worse survival in patients with suspected heart failure (acute and chronic) or heart disease (coronary artery disease [CAD]). Additionally, PR prolongation and P wave prolongation predict increased risk of AF and the greater degrees of PR prolongation and P wave duration predicted higher risks of AF.^27^, ^28^ An increased PR interval is also associated with poor cardiovascular outcomes in patients with AF.^29^ Several studies have shown that PR prolongation in patients undergoing cardiac pacing or receiving cardiac resynchronization therapy (CRT) is an independent predictor of worse prognosis and lower probability of reverse remodelling as well as an increased risk of AF, death, and hospitalization.^30^, ^31^ There are no data indicating whether the degree of PR prolongation portends a worse outcome compared to patients who have lesser degrees of PR prolongation, nor is there information on its prognostic value in acute inferior wall myocardial infarction (MI).

#### 2.2.3 QRS, QT interval, and T‐wave

Over the years, a number of ECG techniques have been developed to assess risk of ventricular tachyarrhythmias (VTs). These have the advantage of being non‐invasive and, often, inexpensive. For almost all of these techniques, there are conflicting data, and not one technique has proven beneficial in patients with structural heart disease. Moreover, studies have varied in their reporting of sudden arrhythmic death vs. total mortality. Among the risk predictors shown to have value are QRS widening and fragmentation, QT prolongation, T‐wave abnormalities, and ventricular ectopy. Although the prognostic value of each ECG parameter in isolation is limited, in combination with additional ECG, imaging, and genetic testing, these parameters can contribute to effective risk stratification.

##### QRS

QRS prolongation has been associated with all‐cause mortality in heart failure patients, implantable cardioverter‐defibrillator (ICD) shocks, and inducibility of sustained VT. QRS prolongation in patients on Class IC antiarrhythmic drugs is a predictor of pro‐arrhythmia, and should be monitored, particularly during exercise. QRS prolongation predicts risk in patients with myotonic dystrophy and in Brugada Syndrome. Additional prognostic information from the QRS is obtained from the signal‐averaged ECG, which amplifies the QRS, averages multiple complexes to reduce noise, and filters out the T‐wave in order to detect late potentials, and provides evidence of slow conduction substrate that associates with risk of re‐entry tachyarrhythmias.^17^ The signal‐averaged ECG has been used to detect risk of ventricular arrhythmias in post‐infarction patients, arrhythmogenic cardiomyopathy, and Brugada Syndrome. Although its specificity is limited, its negative predictive value is high, particularly in survivors of inferior wall myocardial infarction. The signal‐averaged ECG is not useful in patients with underlying bundle branch block. QRS fragmentation, which includes abnormally notched narrow and wide QRS complexes, is associated with the presence of myocardial scar and is also associated with mortality in patients with cardiomyopathy and with Brugada Syndrome.^32^ The presence of an unprovoked type 1 Brugada Syndrome pattern is associated with increased risk as is discussed later in the document.

##### QT interval

Measurement of the QT interval can be complicated by QRS prolongation and by the need to correct for heart rate, as has been described elsewhere.^33^ Despite these limitations, prolongation of the heart rate‐corrected QT interval (QTc) has been associated with mortality in several population studies.^34^, ^35^ In congenital long QT syndrome (LQTS), the length of the QT interval is a major predictor of risk of cardiac events, including sudden cardiac death (SCD). When initiating QT‐prolonging drugs such as sotalol or dofetilide, a QT interval of 500 milliseconds or higher should prompt reduction or discontinuation of the offending drug(s).

##### QT dispersion

This measure of ventricular repolarization heterogeneity is typically defined from the 12‐lead ECG as the QT_max_ − QT_min_. It has been used to predict a wide variety of events, including ventricular pro‐arrhythmia, VTs, although the sensitivity, specificity, and accuracy are poorly defined and highly dependent on the patient population studied.^36^

##### T wave

T wave inversions are common and may be non‐specific or may signal important abnormalities such as ischaemia or hypertrophy. Widespread deep T wave inversions in combination with QT prolongation, such as may occur in acute stress cardiomyopathy, can be associated with torsades de pointes. Abnormal T wave notching can be a clue to abnormal repolarization and is often seen in patients with QT prolongation. Computerized T‐wave analytic techniques such as principal component analysis, T‐wave residuum, flatness, asymmetry, and notching have been developed in an effort to detect and quantify abnormal repolarization and may have particular value in identifying patients with LQTS.^37^, ^38^ Moreover, it has been shown that adding T‐wave morphology characterizations to age, gender, and QTc in a support vector machine model can improve LQTS diagnosis.^39^ However, these additional analytic techniques are not used in routine clinical practice.

The Tpeak‐end interval, measured from the peak to the end of the T‐wave, thought to reflect heterogeneity of repolarization in the heart, has been associated with arrhythmic risk in various populations.^40^ However, considerable controversy remains as to how it should be measured and applied.^41^

T‐wave alternans is a beat‐to‐beat alternation of T wave morphology. When seen with the naked eye, it usually accompanies marked QT prolongation and is a harbinger of immanent torsades de pointes. Analysis of more subtle T‐wave alternans has been used for assessing abnormal and heterogeneous repolarization to predict mortality and arrhythmic risk. Abnormal microvolt T‐wave alternans assessed using the spectral method during graded exercise has a high negative predictive value and has been used to identify a subgroup of patients with reduced ejection fraction who are not likely to benefit from defibrillator implantation.^18^ Microvolt T‐wave alternans analysis cannot be performed when the rhythm is AF, and patients with ventricular pacing have not been studied extensively.

##### Early repolarization

Early repolarization pattern, highly prevalent in the overall population, defined as an elevation of the J point of at least 0.1 mV, may occur in the anteroseptal or inferolateral leads. In 2008, Haissaguerre reported an association of inferolateral early repolarization with increased risk of idiopathic ventricular fibrillation (VF) in a case–control study^42^ and subsequently confirmed in other case–control studies. Exercise testing or isoproterenol testing improved the pattern of repolarization, and the pattern was accentuated with exposure to beta‐adrenergic blockers. In a meta‐analysis of population‐based studies, inferolateral early repolarization was associated with increased risk of arrhythmic death, but the risk was still quite low in general (70/100 000 patient‐years).^43^ It appears that individuals at highest risk have early repolarization in multiple (especially inferior) leads, with high voltage (at least 0.2 mV), and with notching or horizontal/down‐sloping ST segments. Early repolarization is especially prevalent in young men, particularly young black men, and in athletes.^44^ Because the absolute risk of arrhythmic death is so low, asymptomatic individuals with early repolarization, even those with higher risk ECG patterns, do not require further evaluation except when there is a strong family history of sudden cardiac death or when the J point elevation is associated with Brugada syndrome (discussed later in this document) or short QT syndrome (SQT).

#### 2.2.4 Ambulatory electrocardiographic monitoring

In 1984, Bigger et al. showed that ventricular ectopy recorded on a Holter monitor, especially when combined with a low left ventricular ejection fraction (LVEF), predicted a higher risk of mortality in post‐infarction patients compared to those without ectopy.^45^ Non‐sustained VT is also associated with increased risk in patients with arrhythmogenic and hypertrophic cardiomyopathy (HCM). Other data that can be extracted from ambulatory monitoring include heart rate, heart rate variability, and heart rate turbulence measurements, which can predict mortality risk at least in ischaemic cardiomyopathy, but have not been incorporated into clinical practice.^19^, ^46^

### 2.3 Imaging

**Table:** 

Imaging (echo, computed tomography (CT), magnetic resonance imaging (MRI), perfusion)	Class	References
Echocardiography should be used to evaluate EF for risk assessment for primary prevention of sudden cardiac death and the presence of structural heart disease. Alternatively, MRI or cardiac CT can be used.	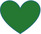	47, 48
Cardiac MRI is useful in assessing aetiology‐driven risk of VT and for the presence of scar or myocardial inflammation.	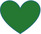	49, 50, 51
Cardiac positron emission tomography may be useful for the assessment of aetiology‐driven risk of ventricular arrhythmias and the presence of scar or myocardial inflammation in patients without CAD.	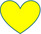	52, 53

#### 2.3.1 Risk assessment of ventricular tachyarrhythmia using imaging modalities

Evaluation for the presence of structural heart disease (SHD) is important for patients suspected of being at risk for sudden cardiac death. Left ventricular ejection fraction remains the key independent parameter for risk stratification of sudden cardiac death and to guide implantation of an ICD.^47^, ^48^ Randomized controlled trials have shown a survival benefit from ICDs in patients with SHD and an EF ≤35%.^54^, ^55^, ^56^ Although EF is currently the only proven imaging modality demonstrated to risk stratify for sudden cardiac death, only 1%‐5% of patients with ICDs, implanted based upon a low EF, require therapies each year and the large majority of patients who receive ICDs will not have ICD therapies over the 3‐year period after implantation.^57^, ^58^ In addition, up to 70% of all sudden cardiac deaths in the community occur in individuals with EF >35%.^58^, ^59^, ^60^ Although the Efficacy of ICDs in Patients with Non‐ischaemic Systolic Heart Failure (DANISH) trial showed that primary prevention ICD in the setting of severe non‐ischaemic cardiomyopathy did not reduce all‐cause mortality in patients on optimal medical therapy for heart failure, ICD implantation was associated with a 50% reduction in arrhythmic death. Of note, within this non‐ischaemic cardiomyopathy population, younger patients (<68 years old) experienced a mortality benefit of 36% if treated with an ICD.^61^

Ejection fraction is most readily evaluated with echocardiography (recommendation level: green), given both lower cost, availability of equipment, and available expertise; however, cardiac MRI or CT can also be used to evaluate EF and SHD, particularly if obtained in combination of other assessment aims, such as CAD or if there is controversy over the quantified EF with echo (recommendation level: green). The imaging modality used to estimate EF has not been shown to determine benefit from ICD.^48^

Additional parameters beyond EF remain to be tested in large studies. Cardiac MRI with late gadolinium enhancement (LGE) can provide important prognostic information and may allow for more accurate assessment of scar. Presence and location of scar can portend a higher risk of sustained VT.^49^, ^50^, ^51^, ^62^, ^63^ In a study of 452 non‐ischaemic cardiomyopathy patients with New York Heart Association Class II or II and EF <35%, ICD implantation was only associated with reduced mortality in the population that had presence of scar on cardiac MRI.^64^ Cardiac positron emission tomography (PET) may elucidate areas of inflammation which may identify inflammatory cardiomyopathies and sarcoidosis, a condition that is associated with higher risk of ventricular arrhythmias in patients without CAD (increased F‐2‐fluorodeoxyglucose uptake) or can be used to identify sympathetic denervation (carbon‐11‐metahydroxyephedrine imaging) or regions of inflammation. Greater sympathetic denervation on PET in a prospective study of ischaemic cardiomyopathy patients was a better predictor of ICD shocks than EF.^65^ Uptake of iodine‐123 meta‐iodobenzylguanidine (MIBG) to evaluate heart to mediastinum ration (H/M ratio) has shown mixed results in predicting arrhythmic death with some studies suggesting additional prognostic benefit for this parameter, while others have not demonstrated additional value.^66^, ^67^ Importantly, the value of these additional parameters in determining risk of sustained VT, VF, or benefit from ICD in various population remains to be clarified. Finally, routine use of viability assessment using PET to guide revascularization in order to reduce risk of SCD remains an area of investigation. In patients with an EF <35% and CAD amenable to revascularization, routine use of PET to guide revascularization was not beneficial in reducing overall mortality.^68^

#### 2.3.2 Imaging modalities for atrial arrhythmias

Echocardiography (transthoracic or transoesophageal) is a valuable tool in patients who present with atrial arrhythmias, specifically atrial flutter and AF, to evaluate for the presence of structural heart disease, left atrial enlargement, and valvular heart disease in order to better define treatment options. Cardiac MRI or CT may also be used if images obtained at echocardiography are not reliable. However, routine use of echocardiography, including atrial strain or atrial function in patients who do not have atrial arrhythmias to assess risk for the development of AF or atrial flutter is not warranted, unless other structural cardiac abnormalities are suspected.

### 2.4 Invasive electrophysiological study

**Table:** 

Invasive electrophysiological study (EPS)	Class	References
EPS is indicated in patients with syncope and previous myocardial infarction, or other scar‐related conditions when syncope remains unexplained after non‐invasive evaluation.	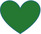	^69^
EPS may be considered in patients with syncope and asymptomatic sinus bradycardia, in a few instances when non‐invasive tests (e.g. ECG monitoring) have failed to show a correlation between syncope and bradycardia	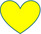	70, 71, 72
EPS may be considered in patients with EF ≤ 40%, without a primary prophylactic ICD indication, and non‐sustained VT in ischaemic cardiomyopathy (MUSTT criteria) to ascertain the presence of sustained VT events.	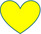	^73^
EPS may be helpful in patients with syncope and presence of a cardiac scar, including those with a previous myocardial infarction, or other scar‐related conditions, when the mechanism of syncope remains unexplained after non‐invasive evaluation.	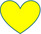	66, 70, 71, 73
EPS may be considered in patients with syncope and bifascicular block, when the mechanism of syncope remains unexplained after non‐invasive evaluation.	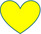	67, 70, 71, 74
EPS may be considered for risk stratification of SCD in patients with tetralogy of Fallot who have one or more risk factors among LV dysfunction, non‐sustained VT and QRS duration exceeding 180 ms.	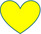	67, 70, 71, 74
EPS may be considered in patients with congenital heart disease and non‐sustained VT to determine the risk of sustained VT or identify SVT that could be ablate.	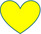	67, 70, 71, 74
EPS may be considered in asymptomatic patients with spontaneous type 1 Brugada ECG pattern, or drug‐induced type 1 ECG pattern and additional risk factors.	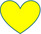	75, 76, 77
EPS is not recommended for additional risk stratification in patients with either long or short QT, catecholaminergic VT or early repolarization.	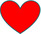	70, 71
EPS is not recommended for risk stratification in patients with ischaemic or non‐ischaemic dilated cardiomyopathy 40 (DCM) who meet criteria for ICD implantation.	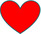	70, 71

Currently, there are a few indications to perform an electrophysiological study (EPS) to further assess the risk of arrhythmias in at‐risk cardiac patients. Such patients include those with structural heart disease, LVEF >35%, pre‐syncope, syncope, palpitations, or markedly abnormal ECG suggesting severe conduction disease. These patients can be considered for an EPS to assess the risk of ventricular arrhythmias and sudden cardiac death to decide on need of an ICD, or to identify conduction disturbances or supraventricular tachycardias that can be treated with ablation or pacing.^70^, ^71^

Patients with ischaemic cardiomyopathy without a primary indication for an ICD, EF ≤40%, and non‐sustained VT on ambulatory cardiac monitoring are candidates for an EPS according to the findings in the MUSTT trial,^73^ in which, 35% of patients with inducible sustained VT had a significantly lower risk of death with an ICD.^66^ The MADIT trial initially also utilized an EPS in post‐MI patients with an EF ≤30%, and non‐sustained VT events to implant an ICD, and showed survival benefit with the ICD.^54^ However, MADIT‐II subsequently eliminated the need for an EPS in post‐MI patients with an EF ≤30% and similarly showed the life‐saving benefit of the ICD in a broader patient cohort.^55^ Therefore, post‐MI patients with an EF ≤30% do not currently need to undergo an EPS to guide decisions on whether to implant an ICD.

In patients with heart failure and EF ≤35%, an EPS is not recommended for risk assessment for the decision on ICD indication. Some centres perform an EPS for inducibility to better characterize induced, sustained VT events, and their response to antitachycardia pacing (ATP), which may potentially help to tailor ICD programming. Furthermore, in patients who have syncope of uncertain origin, an EPS could identify ventricular arrhythmias or document electrical conduction disorders.^67^, ^70^, ^71^, ^74^

In the case of channelopathies, there is no indication for an EPS, except for Brugada syndrome. In Brugada syndrome, EPS may be useful in asymptomatic patients with spontaneous or drug‐induced type 1 pattern, especially when there is a family history of sudden death.^75^, ^76^, ^77^

### 2.5 Implantable loop recorders

**Table:** 

**Implantable cardiac devices**	**Class**	**References**
An ILR is indicated in the evaluation of patients with infrequent recurrent syncope of uncertain origin especially when ambulatory monitoring is inconclusive	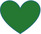	78, 79, 80
An ILR is indicated in patients with syncope and high‐risk criteria in whom a comprehensive evaluation did not demonstrate a cause of syncope or lead to a specific treatment, and who do not have conventional indications for primary prevention ICD or pacemaker.	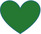	78, 79, 80
An ILR can be considered in patients with palpitations, dizziness, pre‐syncope, frequent premature ventricular complexes (PVCs)/non‐sustained VT, and in those with suspected AF, and following AF ablation.	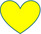	78, 79, 80

#### 2.5.1 Implantable loop recorder to diagnose unexplained syncope/atrial fibrillation with cryptogenic stroke

The implantable loop recorder (ILR) provides long‐term continuous monitoring and improves the diagnosis in patients with unexplained syncope.^81^ In a meta‐analysis of 49 studies that included 4381 participants, the diagnostic yield for the detection of arrhythmogenic syncope was 26.5%.^78^ Moreover, the CRYSTAL‐AF trial^80^ revealed that the ILR can detect subclinical AF following cryptogenic stroke. Still, any benefit of these findings needs to be confirmed in large randomized trials. Early use of the ILR has been advocated by the European guidelines^82^ and in the American guidelines following inconclusive non‐invasive monitoring.^83^ The indications for ILR have been expanded in the current guidelines (Table 2).

**Table 2:** High‐risk and low‐risk criteria for syncope at initial evaluation (Adapted from 2018 ESC Guidelines for the diagnosis and management of syncope^82^)

Syncopal events
Low‐risk
Associated with prodrome typical or reflex syncope (e.g. light‐headedness, feeling of warmth, sweating, nausea, vomiting)
After sudden unexpected unpleasant sight, sound, smell, or pain^a^
After prolonged standing or crowed, hot places
During a meal or postprandial
Triggered by cough, defaecation, or micturition
With head rotation or pressure on carotid sinus (e.g. tumour, shaving, tight collars)
Standing from supine/sitting position
High‐risk
Major
New onset of chest discomfort, breathlessness, abdominal pain, or headache
Syncope during exertion or when supine
Sudden onset palpitation immediately followed by syncope
Presence of structural heart disease especially left ventricular dysfunction and/or history of myocardial infarction
Minor (high‐risk only if associated with structural heart disease or abnormal ECG):
No warning symptoms or short (<10 s) prodrome
Family history of sudden cardiac death at young age
Syncope in the sitting position

ECG, electrocardiogram; VF, ventricular fibrillation.

^a^ Sudden loud sounds (as an alarm clock) may trigger VF in some long QT syndrome patients.

#### 2.5.2 Implantable loop recorder to diagnose atrial and ventricular arrhythmia events

While the ILR can be useful to detect atrial and ventricular arrhythmias, a large cohort study indicated that most of the current use of ILRs is primarily in patients with unexplained syncope (84%), followed by palpitations (13%), and suspected AF (12%).^79^ Another smaller study specifically studying the risk of SCD and arrhythmias in patients with haemodialysis, found that 20% of these patients had SCD or bradyarrhythmia events necessitating pacemaker implantation, and 33% of these patients had an arrhythmic endpoint. Interestingly, the median time to event was 2.6 years, confirming the need for long‐term monitoring. Surprisingly however, bradyarrhythmias were very commonly diagnosed in this cohort suspected to be at high risk for ventricular arrhythmias and sudden cardiac death.^84^ Further studies are needed to establish the role of ILR in risk stratification.

### 2.6 Wearables/direct to consumer

**Table:** 

Wearables/direct to consumer	Class	References
Wearables may provide diagnostic data that contribute to disease detection and management when integrated into the clinical context and physician judgement	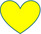	85, 86

The direct to consumer or wearable technology market, comprised of devices that monitor physiological parameters such as heart rate and sleep pattern, is anticipated to grow to 929 million connected devices by 2021.^87^ These devices encompass wristbands, glasses, in‐ear monitors, chest straps, and smart phone‐enabled recording electrode systems or electronic shirts, with varying capacity to monitor heart rate, heart rhythm, blood pressure, physical activity, respiratory rate, blood glucose, and sleep patterns.^88^, ^89^, ^90^ For heart rate monitoring, most wearable devices use photoplethysmography (PPG) technology, meaning they are inherently less accurate than conventional electrocardiography monitoring techniques. Accuracy of various devices varies, with correlation to reference standard ECG monitoring ranging from 0.76 to 0.99.^91^ Recent advances in wearable ECG acquisition include use of direct electrode recording that represents a regulatory approved medical device generating a lead I like rhythm strip, blurring the lines between consumer and medical devices.^92^

A growing body of evidence suggests that these technologies can be harnessed to facilitate arrhythmia detection in the appropriate context. Although marketed as consumer devices, many wearable devices may generate health data comparable to that of medical grade ECG monitors, with several devices migrating to approved medical use.^85^ Despite this promise, there are clear concerns regarding accuracy, particularly false positives in asymptomatic patients where device‐based alerts can raise unwarranted concern and generate low yield screening for disease, with associated costs. Wearable technologies represent an important frontier in health evaluation, with the potential to provide readily accessible health data for large segments of the population, including those not captured by conventional monitoring techniques. Though intended for personal use focused on health promotion and physical activity, wearable technologies promise to invert the traditional paradigm of healthcare delivery, with data collection and health queries often initiated by consumers and not providers. Providers may see wearables as accessible risk stratification tools for detection of AF in high‐risk cohorts (such as high CHADS_2_‐VASC_2_ score patients), and patients may equally present for evaluation after device‐based observations that call into question whether they are at risk. The confluence of these factors is illustrated in the recently presented Apple Heart Study, wherein 419 297 participants were recruited in only 8 months to participate in an AF screening study that deployed a PPG‐based algorithm followed by a 7‐day patch if AF was suspected.^93^ Using a complex tachogram algorithm, 2126 individuals were sent irregular pulse notifications and prompted for a telemedicine visit and 7‐day ECG patch. The authors reported a PPV of 84% for each irregular pulse notification, and 71% for each irregular tachogram. The burden of notifications and the performance of the technology showed promise to inform AF detection in the broader public. Similarly, the Huawei Heart Study evaluated 187 912 individuals that used smart devices to monitor their pulse rhythm, with notification of suspected AF in 424 participants, with a strong relationship between advancing age and detecting AF. The predictive value of the algorithm in the 62% of notified participants that pursued medical evaluation was promising (87%).^94^

Studies evaluating PPG‐based wearables in conjunction with machine‐learning algorithms have shown promise in arrhythmia detection, such as AF.^86^ Studies to date have not focused on ventricular arrhythmia detection. Future wearables will benefit from improved reliability and accuracy, collect additional health and fitness parameters, support chronic disease management, and provide real‐time connectivity and feedback that may supplant conventional medical monitoring. Wearables have the potential to become truly disruptive in our healthcare sector, with large segments of the population accessing cardiac monitoring that the physician must interpret. Currently, we have no data on how the information provided by PPG‐based wearables will affect management and outcomes of patients, or how risk scores derived in other populations such as the CHA_2_DS_2_‐VASc score apply in these previously undetected subjects.

### 2.7 Biomarkers, tissue, genetics

**Table:** 

Biomarkers, tissue, genetics	Class	References
Genetic testing should be considered in several inherited arrhythmic diseases associated with an increased risk of ventricular arrhythmia and SCD	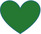	95, 96, 97
MRI with LGE to detect fibrosis and scar may be useful in assessing the risk of arrhythmic events in AF patients and patients with cardiomyopathies	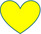	98, 99, 100
Plasma NT‐proBNP may be useful in differentiating patients with higher vs. lower burden of AF	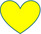	101, 102, 103, 104, 105
Plasma CRP or other inflammatory markers may be useful in risk assessment, for identifying individuals with increased risk of future AF and for identifying individuals with high degree of atrial fibrosis	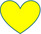	106, 107, 108

The use of biomarkers, tissue biopsy, and genetic assessment can be used for risk assessment in patients suspected of specific arrhythmias or syndromes. The utility of using these tools broadly spans determining arrhythmic risk, refining a clinical diagnosis and estimating prognosis.

#### 2.7.1 Biomarkers

Cardiac myocytes express and secrete natriuretic hormones that have a central function on blood pressure regulation, blood volume, and plasma sodium balance. Levels of B‐type natriuretic peptide (BNP) and its stable N‐terminal peptide pro‐BNP (NT‐proBNP) are increased in AF.^101^ AF burden has been shown to be associated with increased NT‐proBNP.^102^ In a large meta‐analysis consortium, BNP and C‐reactive protein (CRP) associate with AF but only BNP was superior to well‐known clinical variables in AF risk prediction.^103^ Inflammatory processes and fibrosis are central to pathogenesis of AF,^106^, ^109^ and the inflammatory marker CRP is associated with longer AF duration and atrial remodelling.^110^ CRP levels are elevated in patients with permanent AF compared to persistent AF patients and are predictive of recurrent AF after catheter ablation,^111^, ^112^ indicating that CRP levels can be used to identify AF subtypes and evaluate prognosis. Higher levels of CRP correlated to an increased risk of developing AF in general and after acute myocardial infarction.^107^, ^113^ Similarly, the plasma protein YKL‐40 may have diagnostic and prognostic use in AF patients^108^ because plasma serum chondrex (YKL‐40) is associated with atrial fibrosis severity in patients with lone AF.^114^ Patients who experience recurrent AF following ablation have significantly increased YKL‐40 baseline levels, although plasma YKL‐40 is not an independent predictor of recurrent AF.^108^, ^115^ Increasing levels of YKL‐40 have been shown to associate with a two‐fold increased risk of future AF.^116^ Other simple AF biomarkers include body weight and blood pressure, which are also major intervention targets.^117^, ^118^, ^119^, ^120^, ^121^, ^122^

#### 2.7.2 Tissue diagnostics

Tissue diagnostics can be beneficial to differentiate various infiltrative myopathic processes that can contribute to the risk for arrhythmic events. Fibrosis and scarring are well‐recognized substrates for arrhythmia both in atria and ventricles.^109^ Fibrosis may be assessed in atria as well as in ventricular myocardium and its quantification can be used in evaluating the risk of arrhythmia in AF and cardiomyopathies.^98^, ^99^ Specific patterns of scarring can assist in refinement of the diagnosis for infiltrative myopathies, hypertrophic cardiomyopathy, sarcoidosis, arrhythmogenic cardiomyopathy, and amyloidosis. The development and validation of advanced imaging techniques including bio‐metabolic imaging (sarcoid), and contrast enhanced cardiac MRI (amyloid) have largely replaced the need for invasive diagnostics.

#### 2.7.3 Genetics

The majority of clinically applicable genetic testing is intended to be driven by phenotype and the pre‐test probability of specific diagnosis determines the utility of genetic investigation.^95^ Due to incomplete penetrance of genetic arrhythmia syndromes, harbouring a genetic variant with known pathogenicity is almost never solely enough to meet diagnostic criteria for a particular syndrome.^123^

For LQTS, part of the diagnostic framework (along with the ECG biomarker of QT prolongation) can include a positive genetic test.^123^ Moreover, understanding the genetic diagnosis is important for treatment and prognostication. For example, patients with Jervell and Lange‐Nielsen and Timothy Syndrome patients (LQT8) have more malignant clinical courses,^124^, ^125^ and for LQT1 the arrhythmic risk depends partly on which region of the channel the mutation affects.^126^ In catecholaminergic polymorphic ventricular tachyarrhythmia (CPVT),^127^ genetic testing of suspected individuals has a moderately high yield.^95^ Identification of an at risk first‐degree relative of a CPVT affected individual is essential due to the high penetrance but more so the lethality of this syndrome.^123^, ^128^ Similar to LQT1, CPVT due to RYR2 mutations may have some degree of risk depending on where in the ryanodine receptor the mutation falls.^129^ Brugada syndrome can be particularly difficult to clinically diagnose and the utility of genetic testing for improving diagnosis is poor. For patients who are clinically diagnosed with Brugada Syndrome the yield of genetic testing is ~30%,^130^ the majority of whom harbour SCN5a mutations, a gene associated with a plethora of arrhythmia syndromes.^131^, ^132^ Genetic testing can be useful for family members of an appropriately genotype identified proband but is not recommended in the absence of a diagnostic ECG.^95^ Using genetics as part of diagnostic criteria for arrhythmogenic cardiomyopathies will be discussed later in the document. Lastly, genetics in AF is a developing area, but certain primary electrical sudden death syndromes have increased AF association as discussed in Patients with inherited rhythm disease (long QT syndrome/short QT syndrome/catecholaminergic polymorphic ventricular tachyarrhythmia/Brugada syndrome) section. For families with a substantial number of AF cases or in early onset AF,^133^, ^134^, ^135^, ^136^ genetic testing can be considered but the yield is low.

### 2.8 Artificial intelligence

Machine learning is a broad term of artificial intelligence derived from the extraction of patterns from large data sets. The marriage with healthcare analytics and decision processes has been rapidly forwarded with computerized medical records and the creation of large data warehouses.

A deep neural network was created to analyse raw ECG data from an ambulatory heart monitor and classify it into 12 categories based upon the presence of arrhythmia. Machine learning performed very well with an average under the reviewer operating characteristic curve (ROC) of 0.97 and an average F1 score (mean of the PPV and sensitivity) of 0.837; a score better than an average cardiologist (0.780).^137^

Machine learning has been applied to standard ECG characteristics in sinus rhythm to predict incident AF using the eight independent ECG leads (leads I, II, V1‐6) through a convolutional neural network.^138^ The ROC area under the curve for the detection of AF was 0.87 (0.86‐0.88) using the internal validation dataset and 0.87 (0.86‐0.88) using the testing dataset.

In an analysis of the Atrial Fibrillation Prediction Database, a machine learning approach based upon heart rate variability predicted onset of AF with sensitivity of 100%, specificity of 95.6%, and accuracy of 96.2%.^139^ Machine learning based upon ECG characteristics identified left ventricular dysfunction with an area under the curve of 0.93, sensitivity of 86.3%, and specificity of 85.7% including risk of left ventricular dysfunction in those without.^140^

Machine learning has shown accuracy in predicting mortality and risk stratification of patients with CAD.^141^ Machine learning has also been shown to accurately discriminate between athletic hearts compared to hypertrophic cardiomyopathy hearts.^142^ Machine learning has great potential in this area of risk assessment because of the large amount of data contained in the large ECG and clinical datasets available to determine rules.

## 3 HOW TO ASSESS RISK FOR ATRIAL FIBRILLATION IN SPECIFIC POPULATIONS

### 3.1 Patients of advanced age

**FIGURE 1:**
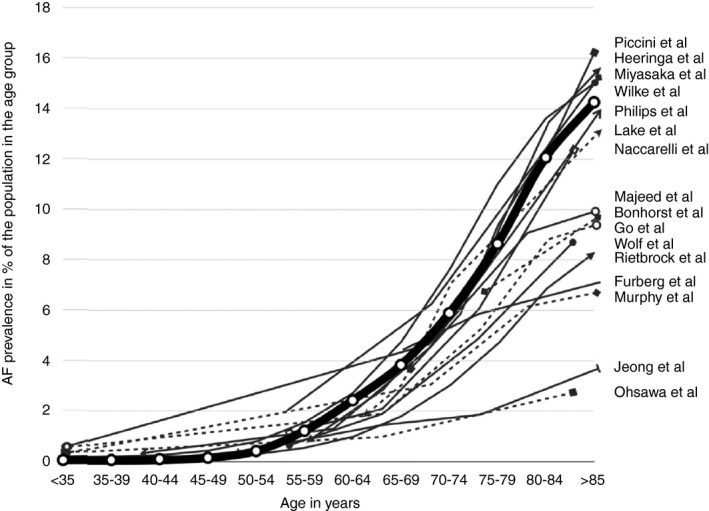
A depiction of the atrial fibrillation prevalence distribution found by each study published to date.^151^ This depiction uses the sex‐specific average rates of AF prevalence, grouped by age. The thick line represents average AF prevalence rates by age group, as derived from a pooled analysis of the individual studies weighted by sample size. (Adapted from Andrade et al.* Circ Res* 2014.) AF, atrial fibrillation.

There is agreement that the prevalence of AF in the general population in the Western world is in the order of 1%‐2%.^143^, ^144^, ^145^ It is estimated that in 2010 there were 33.5 million people in the world with AF of which 20.9 million were men and 12.6 million were women.^146^ During the past 20 years, the age‐adjusted prevalence rates of AF increased for both men and women and similarly the corresponding incidence rates have increased.^146^, ^147^, ^148^, ^149^, ^150^ Age is a major risk factor for the development of AF and in persons younger than 55 years a prevalence of AF around 0.5% is seen whereas in persons older than 85 years AF prevalence is around 15% (Figure 1).^144^ A stepwise increase in AF prevalence with increasing age has been found in several studies.^152^, ^153^ Studies in a multi‐ethnic cohort from the United States has shown large variation in AF prevalence among various race‐ethnicity groups in which AF associated hospitalizations were lower in Hispanics, Chinese, and Black Americans compared to White Americans.^153^ The predominant contributor to the increasing AF prevalence is our aging populations, more widespread use and availability of screening tools, and improved treatment for various heart diseases that enhance longevity.

Among AF patients, those aged younger than 65 years are in general healthier than those older than 65 years.^154^ Life time risks of AF in 55‐year‐old subjects without a history of AF have been found to be 20%‐24% in the Rotterdam study^155^ but considerably higher at 37% in the Framingham study.^134^ The lifetime risk of AF in Asians older than 20 years (1 in 6 for men and 1 in 7 for women; i.e. 14%‐17%) was lower than the risk reported from Western countries.^156^

**FIGURE 2:**
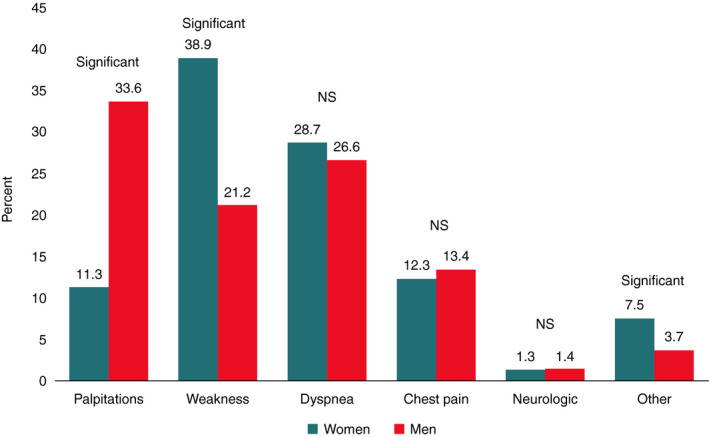
Sex differences in symptoms related to atrial fibrillationy^144^ (Adapted from fig. 2 in Andrade et al*. Can J Cardiol* 2018).

The incidence rates, prevalence, and lifetime risk of AF are higher for men than women. Despite this, the absolute number of women with AF exceeds the total number of men with AF because women live longer than men.^144^ Women have their first episode of AF about 5 years later than men and less commonly have lone AF.^144^ In general, women with AF are more likely to have hypertension or valvular heart disease compared to men.^144^ Women often present with atypical symptoms related to AF (Figure 2). On the other hand, compared to men, women are less likely to have asymptomatic AF, they have a higher symptom burden, they have higher average heart rate during AF and more often longer lasting episodes of AF.^144^ These factors contribute to the observation that women are more likely to contact their physician due to AF‐related symptoms compared to men.

Conflicting results exist with respect to risk of stroke secondary to AF and its prognosis in women compared to men.^157^, ^158^, ^159^ There does not seem to be a gender difference with respect to development of dementia secondary to AF, although women have higher rates of dementia than men in general.^145^, ^157^

Since both AF and stroke are highly associated with age and stroke may occur as a complication of AF it seems reasonable to consider screening for this arrhythmia in elderly populations. Several studies are ongoing and expected to be finalized within the next couple of years. These studies are expected to guide us with respect to cost‐effectiveness of these screening strategies.

### 3.2 Patients with heart failure

**Table:** 

**Investigations needed to assess risk for AF in patients with heart failure**	**Class**	**References**
A careful evaluation of clinical characteristics known to be associated with increased risk for AF should be performed	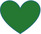	^160^
Frequent interrogation or remote monitoring of stored arrhythmia episodes in device implanted HF patients should be performed in order to diagnose AF and allow its early management	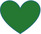	^161^
Echocardiography is useful in identifying cardiac characteristics associated with a higher risk for AF	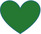	^162^
Cardiac MRI may be considered in identifying degree of atrial fibrosis and scar	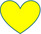	^163^
Use of biomarkers may be considered for identifying individuals with increased risk of future AF and for identifying individuals with high degree of atrial fibrosis	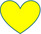	107, 164, 165
Searching for common genetic variants associated with AF risk by genetic molecular analysis has not been found to be useful in a routine clinical setting	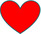	^166^

Due to common risk factors like age, hypertension, diabetes, obesity, and sleep apnoea, AF and HF are intricately linked and share common pathophysiologic mechanisms. Atrial fibrillation occurs in more than half of individuals with HF and presence of both carries greater mortality risk compared with those without either condition.^167^

In the particular case of cancer treatment, HF is also a common consequence of cardiotoxicity associated with some chemotherapeutic agents, including anthracyclines, human epidermal growth factor receptor 2 (HER2), and proteasome inhibitors. In this setting, isolated cases of AF have been reported. Even if the exact mechanism of these arrhythmias induced by such drugs remains largely unknown, it seems plausible that the negative effect on the cardiac systolic function also plays a central role.^168^

**FIGURE 3:**
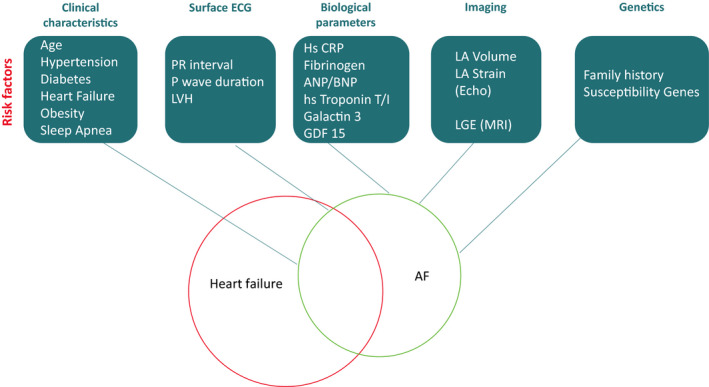
Investigations and associated risk factors useful to predict the development and progression of AF in HF patients. AF, atrial fibrillation; BNP, B‐type natriuretic peptide; CRP, C‐reactive protein; ECG, electrocardiogram; HF, heart failure; LA, left atrium; LGE, late gadolinium enhancement; LVH, left ventricular hypertrophy; MRI, magnetic resonance imaging.

Given the deleterious effects of AF in HF patients, significant interest has been directed to risk factors predicting the development and progression of this arrhythmia (Figure 3).

#### 3.2.1 Clinical risk factors

Older age and male gender are associated with a higher risk of developing AF.^160^ Diabetes confers a 1.4‐ to 1.6‐fold higher risk for AF.^160^ Because of its high prevalence in the general population, hypertension is responsible for more AF in the population (14%) than any other risk factor.^160^ Obesity and sleep apnoea are independent risk factors for AF.^169^ AF incidence also increases in case of renal or thyroid dysfunction.^170^, ^171^

With regard to HF and the type of underlying heart disease, prevalence of AF increases significantly with the severity of HF symptomatology. Among the valvular diseases, the left‐sided valve stenoses have the highest prevalence rates of AF. In addition, the presence of CAD or hypertrophic cardiomyopathy is a significant risk factor for incidence and progression of AF.^172^ Finally, in congenital heart disease patients, substantial AF rates appear decades before their onset in the general population.^173^

#### 3.2.2 Electrocardiography

Electrocardiogram‐derived variables, such as the PR interval, ECG‐based left ventricular hypertrophy (LVH), P wave indices like P wave duration, area, and terminal force have been used in various AF prediction models but their additive value over other clinical risk factors is minimal.^174^ Short duration Holter monitoring is not useful for AF detection in asymptomatic patients. Longer duration monitoring with external or implantable loop recorders may help when paroxysmal AF is suspected. In addition, frequent interrogation or remote monitoring of Holter memories in device implanted HF patients is mandatory in order to diagnose AF and allow its early management.^161^

#### 3.2.3 Biomarkers

Markers of inflammation (high‐sensitivity CRP, fibrinogen), atrial overload (atrial and B‐type natriuretic peptides), myocardial ischaemia (high‐sensitivity troponin T and I), cardiac fibrosis (galectin‐3), and others (soluble ST2, growth differentiation factor‐15), have been studied to predict AF incidence.^165^ Of these, only natriuretic peptides have consistently demonstrated added predictive value beyond information on clinical variables.^164^, ^165^

#### 3.2.4 Imaging

Many echocardiographic variables have been associated with a significantly higher AF recurrence rate. Possibly, left atrial volume is superior to left atrial diameter in predicting progression to persistent AF. Speckle left atrial strain and stiffness index can also predict the maintenance of sinus rhythm after cardioversion for AF.^162^

Concerning MRI, the amount of left atrial enhancement quantified on MRI with LGE may be helpful to predict progression of AF,^163^ but the reproducibility of such findings remains controversial.

#### 3.2.5 Genetics

A family history of AF in a first‐degree relative independently increases AF risk two‐fold.^175^ Recent research has identified several common genetic variants associated with the risk of AF.^136^ Further studies are required to evaluate whether genetic information improves our ability to predict AF on top of clinical variables.

Risk assessment of AF in patients with HF can be carried out at first by considering the clinical features, comorbidities, and underlying aetiologies. It can be further refined by more sophisticated investigations.

### 3.3 Patients with obesity, hypertension, diabetes, sleep apnoea, or structural heart disease

**Table:** 

Patients with obesity, hypertension, diabetes, sleep apnoea, or structural heart disease	Class	References
Clinical risk factors should be assessed to help identify incident AF and its complications	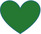	^176^
Clinical risk scores may be useful to identify risk for incident AF	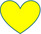	177, 178, 179

The assessment of underlying AF in people at higher risk for AF can be considered from opportunistic perspective, or the consideration of clinical risk prediction tools.^180^ Many patients with common conditions that may predispose to AF, such as obesity, sleep apnoea, hypertension, or SHD should or would be attending specialist clinics for their assessment and/or follow‐up. Hence, an opportunistic strategy of pulse palpation and clinical assessment (e.g. symptoms) followed by appropriate ECG monitoring to confirm AF would be an appropriate and cost effective method for screening.^181^ In general, clinical scores have been less useful as most only have modest predictive value for identifying the population at risk; ultimately, these patients would also require their AF documented. A strategy of using risk scores to target high‐risk patients for more intense screening efforts merits consideration.

The systematic review by Allan et al*.* ^176^ found that in relation to the relative risk of incident AF:

- For every 1‐10 kg/m^2^ increase in body mass index (BMI), or BMI ≥25‐30 kg/m^2^, all 19 reports showed significant direct associations (from 1.04 [1.02‐1.05] to 2.24 [1.41‐3.58]).
- For every 10‐22 mm Hg increase in systolic blood pressure, or systolic blood pressure ≥160 mm Hg, most reports showed significant direct associations (from 1.14 [1.05‐1.25] to 2.63 [1.83‐3.78]).
- For diabetes mellitus (type unspecified), eight reports showed a direct but non‐significant (from 1.02 to 1.49) and six reports showed significant direct associations (from 1.17 [1.16‐1.19] to 1.80 [1.30‐2.60]).

Many of these conditions are present concomitantly. Also, obesity and hypertension are commonly associated with sleep apnoea, which is another risk for incident AF.

Obesity has been associated with incident AF,^182^ but clinical trial data have a suggestion of an ‘obesity paradox’ whereby overweight AF patients tended to have improved outcomes; however, the relationship between obesity and outcomes from real‐world observational cohorts are less clear.^183^, ^184^, ^185^ In a systematic review of trial and real‐world evidence, there was suggestion of an obesity paradox in AF patients, particularly for all‐cause and cardiovascular death outcomes.^184^ An obesity paradox was also evident for stroke/systemic embolic event outcomes in the non‐vitamin K antagonist oral anticoagulant (NOAC) trials, with a treatment effect favouring NOACs over warfarin for both efficacy and safety that was significant only for normal weight patients. Nonetheless, proactive management of obesity is part of the lifestyle advice for patients with AF.

On a population basis, hypertension is the most common aetiological factor for AF, and contributes to its complications. Indeed, AF can be regarded as a manifestation of hypertension target organ damage. The optimal blood pressure targets in AF patients have been described, being 120‐129/<80 mm Hg.^186^ Also, longer hypertension duration is associated with the increased risk of ischaemic stroke; however, this long‐term effect of hypertension duration can be attenuated by long‐term strict SBP control throughout the entire duration of hypertension.^187^

Poor diabetes control is associated with incident AF. In the diabetic AF patient, longer disease duration is related to a higher risk of stroke/thromboembolism in AF, but not with a higher risk of anticoagulant‐related bleeding.^188^ These risks were similar for Type 1 and Type 2 diabetes.^189^ Evidence of other target organ damage such as diabetic retinopathy increased risk, although it did not add to the predictive value of risk assessment using the CHA_2_DS_2_‐VASc score.^190^ Indeed, the ATRIA study also confirmed that duration of diabetes is a more important predictor of ischaemic stroke than glycaemic control in patients who have diabetes and AF.^191^

Unsurprisingly SHD is a potent risk factor for incident AF, as well as its complications, such as stroke and HF.^177^, ^192^ Systolic HF is one of the components of the simple C2HEST score (Chronic obstructive pulmonary disease and CAD [1 point each]; hypertension [1 point]; elderly [age ≥75 years, 2 points]; systolic HF [2 points]; thyroid disease [hyperthyroidism, 1 point]) which has been derived and validated in a large cohort of AF patients.^177^ This score could potentially be considered to target the high‐risk patients that may be suited for more intense screening for incident AF, e.g. post‐stroke where the C2HEST score was superior to the other scores such as the Framingham score.^178^ The risks of AF with associated valvular heart disease are well recognized, as recently discussed in an EHRA position document.^193^ In terms of HF, there is a link between AF complications and HF, whether HF with a reduced EF (HFrEF) or HF with a preserved EF (HFpEF).^194^ In the CHA_2_DS_2_‐VASc score, the ‘C’ component refers to recent decompensated HF, irrespective of the EF, or the presence of moderate‐severe systolic dysfunction whether asymptomatic or not.^7^ Of note, the CHA_2_DS_2_‐VASc score is predictive of stroke in HF, whether or not AF is present.^195^

### 3.4 Patients who have undergone cardiac surgery

**Table:** 

Patients who have undergone cardiac surgery	Class	References
Heart rhythm monitoring for 4‐7 d is recommended for detection of post‐operative AF	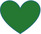	196, 197, 198
Patients with post‐operative AF may undergo follow‐up rhythm monitoring to assess for the presence of symptomatic and asymptomatic arrhythmias	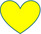	196, 197, 198, 199

Post‐operative AF remains the most common complication following cardiac surgery and its incidence ranges between 20%‐50% across numerous studies.^196^ This risk increases from isolated CABG surgery, to valvular surgery, and in turn to concomitant CABG/valvular surgery.

Risk factors for developing AF may be divided into procedural‐ and patient‐related factors. Procedural‐related risk factors include type of surgery, mitral valve surgery, use of intra‐aortic balloon pump, longer cardiopulmonary bypass and aortic clamp times, and perioperative issues such as inflammation, infection, fluid overload, inotropic use, atrial ischaemia, hypokalaemia, and hypomagnesaemia. Patient‐related risk factors include advanced age, history of AF, history of HF, renal failure, hypertension, chronic obstructive pulmonary disease, post‐operative withdrawal or absence of beta‐blocker, or angiotensin‐converting enzyme inhibitor (ACE inhibitor) therapy.^197^, ^200^ Left atrial remodelling predisposes to post‐cardiac surgery AF, with risk factors such as enlarged left atrial size, diastolic dysfunction, LVH, obesity, obstructive sleep apnoea, and the CHADS_2_ and CHA_2_DS_2_‐VASc score further predisposing to post‐operative AF.^197^, ^201^, ^202^

The majority of post‐cardiac surgical AF occurs within the first 4 post‐operative days, and is most common on the 2nd post‐operative day, while recurrences are most common on the 3rd post‐operative day.^197^, ^203^ In another study of CABG patients, 94% of post‐operative AF occurred by the 7th post‐operative day.^198^ Hence rhythm monitoring such as inpatient telemetry or ECG for post‐operative AF should focus on this time frame.

While post‐cardiac surgical AF likely occurs as a result of the interaction between acute perioperative triggers and the underlying atrial and cardiac substrate, its occurrence identifies a subset of patients associated with long‐term morbidity and mortality. In a study of patients who underwent CABG, post‐operative AF conferred an eight‐fold increased risk of future AF and doubled cardiovascular mortality on long‐term follow‐up.^199^ Follow‐up rhythm monitoring, for example with ECG or Holter monitoring is advisable in this subset of patients particularly in the setting of symptom development. There is emerging data on the use of implantable cardiac monitors for long‐term monitoring of this subset of patients. While implantable cardiac monitors allow continuous long‐term monitoring for arrhythmias and asymptomatic arrhythmias, the risk–benefit ratio is balanced by the arrhythmia detection rate beyond the immediate post‐operative period and level of invasiveness of the monitoring device. Its routine use will depend on further results from prospective medium to long‐term studies.

### 3.5 Patients with cryptogenic stroke

**Table:** 

Patients with cryptogenic stroke	Class	References
Patients should initially undergo brain diffusion‐weighted MRI imaging for the diagnosis of cryptogenic stroke.	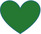	204, 205
AF is more likely to be detected after cryptogenic stroke with more intense investigation with longer and more sophisticated monitoring.	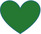	205, 206, 207
Long‐term ECG monitoring techniques, such as trans‐telephonic ECG monitoring or cardiac event recorders or ILR can increase yield of AF diagnosis after cryptogenic stroke in selected patients.	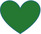	205, 206
The use of an ILR should be considered for detecting AF in selected patients who are at higher risk of AF development, including the elderly, patients with cardiovascular risk factors or comorbidities.	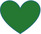	80, 207
TOE may lead to the reclassification of cryptogenic stroke because many cases are embolic and due to a cardiogenic source, mainly AF.	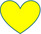	205, 206

**FIGURE 4:**
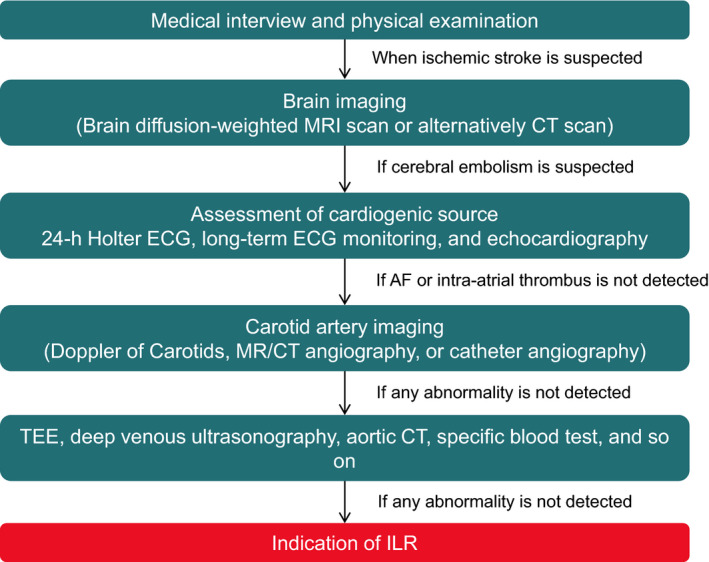
Proceeding of evaluation for cryptogenic stroke. AF, atrial fibrillation; CT, computed tomography; ECG, electrocardiogram; ILR, implantable loop recorder; MRI, magnetic resonance imaging; TOE, transoesophageal echocardiography.

Cryptogenic stroke is defined as ischaemic stroke of undetermined aetiology.^208^ The diagnosis of cryptogenic stroke is generally made by exclusion. Although cryptogenic stroke includes few potential causes, such as paradoxical embolism through a patent foramen ovale, atrial septal aneurysm, and aortic arch atheroma, the majority of cases are thought to be caused by cardio‐embolism due to undetected paroxysmal AF.^205^ For the diagnosis of cryptogenic stroke or a suspected transient ischaemic attack (TIA), patients should initially undergo brain imaging. Diffusion‐weighted MRI is more recommended than any other MRI sequence or CT as brain imaging, except when contraindicated.^204^, ^205^ Advances in cardiac imaging techniques such as transoesophageal echocardiography (TOE) have prompted the reassessment of cryptogenic stroke because most cases are thought to be embolic due to a cardiogenic source, mainly AF. Transoesophageal echocardiography can easily detect a thrombus of the left atrial appendage, particularly with contrast enhancement, which cannot be detected using conventional transthoracic echocardiography. Transthoracic echocardiography with contrast could be useful to detect a left ventricular thrombus (Figure 4).

The detection of permanent or persistent AF is relatively easy, whereas that of paroxysmal AF is more difficult. Current guidelines recommend the use of ECG monitoring among patients with ischaemic stroke including cryptogenic stroke and TIA for whom transient (paroxysmal) AF is suspected and no other causes of stroke are identified.^205^, ^206^ First, 24‐hours Holter ECG is performed to detect the AF burden. If undetected, other long‐term ECG monitoring techniques such as trans‐telephonic ECG monitoring or cardiac event recorders (a symptom event monitor or a ILR) may be attempted as alternative methods. A meta‐analysis indicated that a longer duration of ECG monitoring is associated with an increased detection of new AF when examining monitoring time as a continuous variable. Studies with monitoring lasting ≤72 hours detected new AF in 5.1% of cases, whereas monitoring lasting ≥7 days detected AF in 15% of cases.^209^ The proportion of new diagnosis of AF was increased to 29.1% with 3‐months extended monitoring. Recently, smartphone‐based ECG recording systems have been developed and conferred acceptable sensitivity and specificity of detecting AF^191^ (see Wearables/direct to consumer section).

The use of an ILR is indicated for detecting the presence of AF or arrhythmia burden that might cause ischaemic stroke in selected patients, for example those who are at higher risk of AF development including elderly, patients with cardiovascular risk factors or comorbidities. An ILR is a useful tool for detecting arrhythmias. In the CRYSTAL AF study, AF was newly detected in 8.9% of patients with an ILR by the 6th month compared with 1.4% among those receiving conventional ambulatory ECG monitoring, increasing further to 12.4% by 12 months compared with 2.0% in conventional monitoring.^80^ A similar outcome was observed in the EMBRACE trial, in which AF was newly detected in 16.1% of patients who received 30‐day ILR compared with 3.2% who received ambulatory 24‐hours monitoring.^210^ A systematic review indicated that AF was newly detected in nearly one‐quarter of patients with stroke or TIA by sequentially combining cardiac monitoring methods: 7.7% in phase 1 (emergency room), 5.1% in phase 2 (in‐hospital), 10.7% in phase 3 (first ambulatory period), and 16.9% in phase 4 (second ambulatory period consisting of trans‐telephonic ECG monitoring, cardiac event recorders, and ILR), and 23.7% in the overall detection after all phases of sequential ECG monitoring.^207^ Thus, if we ‘look harder, look longer and look in more sophisticated ways’ we are more likely to detect AF. It is possible that if we use clinical risk stratification (e.g. the C2HEST score) to identify patients post‐stroke at high risk of incident AF, targeted intensive monitoring can be applied.^211^

### 3.6 How to assess high risk of atrial fibrillation in professional athletes

**Table:** 

Atrial fibrillation in athletes	Class	References
In athletes who participate long term in endurance exercises with symptoms of arrhythmia screening for AF is recommended	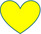	^212^
Risk assessment for AF risk in athletes may include the duration and intensity of exercise as a potential modifiable risk factor	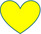	213, 214

#### 3.6.1 Atrial fibrillation risk in athletes—general

Paroxysmal or persistent AF is common in athletes and may be autonomically mediated or triggered by other supraventricular tachycardias.^215^ AF is the primary arrhythmia observed in middle‐aged athletes.^216^ AF in athletes tends to be paroxysmal, vagally mediated, and highly symptomatic.^213^ The mechanism of increased AF risk at either end of the physical activity spectrum likely includes autonomic, structural, inflammatory, and fibrotic changes to the heart. For example, increased vagal tone, which is often observed in the endurance athlete, has been shown to result in a short atrial refractory period, and thus initiates AF.^217^

#### 3.6.2 Atrial fibrillation risk in athletes—exercise paradox

Recent studies have observed a U‐shaped risk relationship of physical activity to AF. At one end of the spectrum, a large observational study^218^, ^219^ of people showed that those at the lowest levels of physical fitness had a 5‐fold increased risk of AF.^220^ Increasing the physical activity of sedentary patients could help reduce the risk or burden of AF. Long‐term endurance training, as well as a sedentary lifestyle,^221^ increase chronic systemic inflammation, which in turn could also facilitate AF.^106^ For example, one randomized study demonstrated that just 12 weeks of moderate‐intensity physical activity decreased the AF burden by 41%.^222^ Of the physically inactive with AF, the obese might benefit the most from moderate levels of physical activity.^220^ In contrast, a meta‐analysis of 655 endurance athletes also demonstrated a five‐fold increased risk of AF.^212^ Of these studies, increased AF risk was generally only observed with the highest levels of physical activity that was maintained over a prolonged period of time.^213^, ^214^ One uniform explanation for the exercise paradox is that both long‐term endurance training and a sedentary lifestyle increase chronic systemic inflammation.

#### 3.6.3 Atrial fibrillation risk in athletes—structural cardiac changes

Most studies have shown structural changes in endurance athletes, which have resulted in the term athlete’s heart. These changes include dilatation of all four heart chambers, increase in left ventricular mass, and mild right ventricular hypertrophy.^223^ Studies show that moderate physical activity might reduce inflammatory markers.^224^, ^225^, ^226^ Extreme levels of exercise are a known cause of cardiac fibrosis, particularly in hinge point locations of the heart, such as the right ventricle; however, the significance of MRI‐detected fibrosis remains controversial.^227^ Athletes who experience higher levels of fibrosis also have higher levels of coronary calcium.^228^ In turn, fibrosis is a well‐established risk factor of AF.^163^ In one study, the fibrotic changes caused by vigorous exercise were reversed after an 8‐week period of physical activity cessation.^229^ Among young elite athletes, age, years of competition, and echocardiographically measured parameters, including left atrial anterior–posterior diameter and atrial strain, were associated with higher AF risk.^230^, ^231^ Although increasing physical activity might reduce AF in sedentary patients, decreasing physical activity levels in elite endurance athletes may also reduce AF.^215^ Currently, the role of deconditioning to lower AF risk in elite athletes for primary or secondary prevention of arrhythmia requires prospective evaluation.

### 3.7 Patients with inherited rhythm disease (long QT syndrome/short QT syndrome/catecholaminergic polymorphic ventricular tachyarrhythmia/Brugada syndrome)

**Table:** 

Patients with inherited rhythm disease	Class	References
Patients with certain inherited arrhythmia syndromes are at higher risk for AF and benefit from symptom‐driven and periodic surveillance	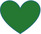	^123^
Evaluation should include non‐invasive symptom‐driven surveillance for patients at risk for AF and periodic non‐invasive surveillance for asymptomatic patients	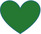	232, 233, 234
EPS to determine atrial AF substrate or susceptibility is not useful.	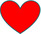	^123^

Some patients with primary electrical sudden death syndromes have an increased AF association, including Brugada Syndrome, LQTS, SQT, and catecholaminergic polymorphic ventricular tachycardia (CPVT). These patients are at risk for arrhythmia symptoms from AF and are vulnerable to AF consequences such as pro‐arrhythmia and inappropriate ICD shocks.

Brugada Syndrome is characterized by ST‐segment elevation in the precordial ECG leads and increased risk of SCD due to VF.^235^ Brugada Syndrome is associated with a higher incidence of SVTs, and AF is the most common SVT in these patients.^236^, ^237^ AF susceptibility has been described with patients harbouring mutations in SCN5A, CACNA1C and patients without an identified genotype,^234^, ^238^ suggesting a lack of genetic AF specific risk but AF may be more prevalent with more advanced disease.^239^, ^240^ Importantly, AF events can be pro‐arrhythmic for Brugada Syndrome patients^123^, ^241^ and contribute to the high inappropriate ICD shock rates for Brugada Syndrome patients.^241^

Long QT syndrome is a genetically heterogeneous syndrome associated with mutations in 17 different genes with some unique phenotypic characteristics based on genotype and electrically results in prolonged repolarization and risk for fatal ventricular arrhythmia torsade de pointes. While generally, prolonged repolarization inhibits AF initiation and this is the mechanism for Vaughn–Williams Class III anti‐arrhythmic drugs, rare patients with LQTS have also been noted to have AF.^242^, ^243^ This has been limited to single case reports and unverified, 1.7% of patients in a LQTS cohort, which is a higher prevalence than the general population.^133^, ^244^ Not surprisingly, some genes associated with AF in LQTS have overlap with familial AF: LQT1 (KCNQ1), LQT2 (KCNH2), LQT3 (SCN5a), and LQT7 (KCNJ2). However, for potassium channels, in LQTS the genetic defect results in ‘loss of function’ in contrast to a ‘gain of function’ in familial AF.^245^, ^246^ It is less clear how prolonged repolarization results in AF susceptibility but it may involve similar mechanisms to torsade de pointes^247^ or perhaps dispersion of repolarization and induction of early after depolarizations.^248^, ^249^

From an electrical substrate standpoint, it is easier to understand why SQTS and CPVT are associated with AF. Short QT syndrome is a rare disorder caused by a gain of function of potassium channels encoded by KCNQ1, KCNH2, and KCNJ2, causing a shortening of the action potential and manifests in the atrium by a decreased atrial refractory period and electrical substrate for AF.^250^, ^251^, ^252^ CPVT is an autosomal dominant disorder associated with polymorphic VT and bidirectional VT due to cellular calcium overload caused by mutations in calcium handling genes.^253^, ^254^, ^255^ A reciprocal condition can exist in the atria of patients with CPVT with AF susceptibility and has been shown to be more prevalent in patients with more dysfunctional ryanodine receptor2 channels.^256^ It is also unclear how clinically significant AF is for CPVT patients. However, the failure to recognize and treat AF can result in inappropriate shocks, pro‐arrhythmia, and death.^232^, ^233^

These issues highlight the need for AF recognition, ICD programming to reduce the risk of inappropriate shocks, and preventative treatment. Because of the small cohort sizes and lack of systematic studies, it is difficult to prospectively estimate AF risk. Invasive EP studies evaluating atrial refractory periods, conduction time, and AF inducibility have been inconclusive^236^, ^237^ and either not systematically evaluated in large populations or are contraindicated (LQTS and CPVT).^123^ We support vigilant non‐invasive surveillance in these conditions. For patients with ICD, close follow‐up is needed to decipher and to adjudicate if atrial arrhythmias are present and proactively increase the rate cut‐off for VF detection and turn SVT discriminators on, if available. Patients without ICD, but suggestive symptoms, should undergo ambulatory monitoring and asymptomatic patients should have surveillance monitoring done every 1‐2 years. Treatment is not the focus of this article, but it should be recognized that many AADs can worsen the electrical substrate for inherited arrhythmia patients (i.e. LQTS, Brugada Syndrome) and care should be taken when choosing antiarrhythmic drugs.

## 4 HOW TO ASSESS RISK FOR ADVERSE OUTCOMES IN PATIENTS WITH ATRIAL FIBRILLATION

### 4.1 Risk assessment for stroke/transient ischaemic attack/cognitive decline

**Table:** 

Risk assessment for stroke/TIA/Cognitive decline	Class	References
A risk factor‐based approach is recommended for stroke risk assessment in patients with AF	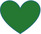	8, 257
Cognitive assessment should be performed in AF patients where there is suspicion of cognitive impairment.	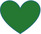	258, 259
Assessment of cognitive function may be multifaceted, and cognitive impairment screening by available tools is just one component.	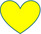	^258^
Risk reduction of cognitive dysfunction and its comorbidities in AF may include risk assessment for vascular disease and/or Alzheimer’s disease.	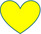	258, 260
General health measures may reduce the concomitant risks of AF and stroke, with a putative benefit on cognitive function.	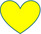	1, 2

Patients with AF have increased mortality and morbidity compared with non‐AF patients and may experience significant adverse events. Stroke and thrombo‐embolic events are well known complications that can be avoided by oral anticoagulation. Since the risk of individual patient differs significantly, an individual risk assessment is necessary. Several stroke risk scores, including ABC‐stroke (age, biomarker, clinical history), ATRIA (Anticoagulation and Risk Factors in Atrial Fibrillation), GARFIELD (Global Anticoagulant Registry in the FIELD), and Qstroke have been proposed as support tools for the decision on oral anticoagulation.^261^, ^262^, ^263^, ^264^ However, the one currently most widely applied and recommended by international guidelines is the CHA_2_DS_2_‐VASc risk scheme. According to CHA_2_DS_2_‐VASc, patients with score of ≥1 in a male or ≥2 in a female should be considered for stroke prevention strategies.^265^, ^266^, ^267^, ^268^ Nevertheless, it has to be kept in mind that no stroke risk scheme has perfect predictive accuracy.

Another major adverse effect of AF is impairment of cognitive function.^258^, ^259^ Multiple risk factors for dementia have been identified in the general population, including modifiable and non‐modifiable ones.^269^ Apart from these AF‐non‐specific risk factors, AF may lead to cognitive impairment by multiple mechanisms. These include apparent stroke, silent stroke but also other mechanisms that are independent of thromboembolism.^270^ A detailed description of the association between AF and cognitive impairment and possible preventive mechanisms has been provided recently in an expert consensus document.^258^ In terms of prevention of cognitive impairment in AF patients, there is evidence that early and effective use of oral anticoagulation in patients with stroke risk factors reduces the rate of cognitive decline and currently, this represents the most important preventive strategy. Consequently, the main risk assessment for cognitive impairment in AF patients is the assessment of stroke risk factors, preferably by use of the CHA_2_DS_2_‐VASc risk scheme that can guide the decision on oral anticoagulation. When cognitive impairment is suspected, brief screening tools such as General Practitioner Assessment of Cognition (GPCOG), Mini Mental State Examination (MMSE) and Montreal Cognitive Assessment (MOCA), and Informant Questionnaire on Cognitive Decline in the Elderly (IQCODE) may be applicable.^258^ In addition, more comprehensive assessments may be done after appropriate referral to a psychiatrist, geriatrician, or neurologist.^258^

### 4.2 Risk assessment for stroke/transient ischaemic attack status post‐left atrial appendage occlusion/ligation

**Table:** 

Risk assessment for stroke/TIA after LAA occlusion/ligation	Class	References
TOE after 6 weeks and if necessary after 1 year is useful for detecting peri‐device residual flow, incomplete appendage ligation, or device‐related thrombus to identify patients at higher risk of stroke	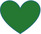	271, 272
Clinical features such as previous TIA/stroke, persistent AF, low LVEF, vascular disease, and early discontinuation of anticoagulation may be helpful to guide decisions regarding imaging for device related thrombus	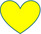	273, 274
Multi‐detector CT and cardiac CT angiography have been found to be equivalent to TOE to detect peri‐device flow	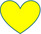	275, 276
After surgical occlusion or exclusion of the left atrial appendage, imaging may be useful to look for a residual appendage and its function or a residual leak after ligation to guide decisions regarding anticoagulation	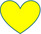	277, 278, 279

Left atrial appendage (LAA) occlusion/ligation using one of several devices or surgical techniques has been developed as an alternative to anticoagulation in high‐risk patients with non‐valvular AF.^280^, ^281^, ^282^ The maximum experience has been with the Watchman device (Boston Scientific), which has been found to be non‐inferior to warfarin in patients who are still candidates for short‐term warfarin treatment.^283^, ^284^, ^285^ Results of comparison between LAA occlusion/ligation and NOACs are awaited. Current guidelines recommend use of LAA occlusion as a possible strategy in patients having contraindications to long‐term anticoagulation.^279^

The residual risk of stroke/TIA following LAA occlusion/ligation can be related to procedural or patient related risk factors. Among the procedure related factors, peri‐device leak, and device‐related thrombus are important factors for thrombo‐embolic events in short and medium term after the procedure. Stroke risk is significantly elevated in patients in whom LAA ligation fails after surgical^286^ or percutaneous approaches.^287^

Post‐procedure surveillance is therefore important to assess long‐term risk of stroke and need for continued anticoagulation. These may be detected on TOE immediately or after few weeks/months.^271^, ^272^ Multidetector CT and cardiac CT angiography have been compared with TOE and found to be an effective alternative technique to detect peri‐device flow.^275^, ^276^ Device‐related thrombus is seen in 3%‐7% of patients after LAA closure, and leads to a 3‐4 fold higher risk of stroke.^273^, ^274^ Factors predicting device‐related thrombus are previous TIA/stroke, persistent AF, low LVEF, vascular disease, and early discontinuation of anticoagulation.^273^, ^274^

If surgical LAA ligation fails or is incomplete, stroke rates are significantly increased. Similarly, with percutaneous closure devices, residual LAA leaks were associated with increased risk of thromboembolism in excess of that associated with baseline risk factors or echocardiogram findings.^286^

### 4.3 Risk for heart failure incidence and progression

**Table:** 

Risk for heart failure incidence and prognosis	Class	References
Screening for AF in patients with HF should be performed because of the increased risk of adverse cardiovascular outcomes in combination more than the risk conveyed by either disease state alone.	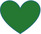	288, 289
Interval use of echocardiography and arrhythmia directed monitoring for development of AF‐induced cardiomyopathy and risk assessment over time should be part of standard follow‐up for patients with AF.	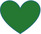	290, 291

Atrial fibrillation and HF are conditions that coexist in many patients, and sometimes it will be difficult to establish if HF was the cause of AF or AF caused HF (tachycardia‐induced cardiomyopathy).^288^, ^292^ In the Framingham study, 41% of patients with AF and HF developed HF first, 38% developed AF first, and in the remaining 21%, AF and HF occurred at the same time.^289^ AF is associated with a three‐fold increased risk of incident HF.^293^ In trials of patients with chronic systolic heart failure, the prevalence of AF was 4% in patients with Class I symptoms, 10%‐27% in patients with Class II‐III symptoms, and 50% for those with Class IV HF symptoms.^291^ Additionally, aging and the structural and neurohormonal changes in HF make the development and progression of AF much more likely. The risks of developing an AF‐induced cardiomyopathy appear to be related to the ventricular rate during AF and the duration of AF. However, the precise incidence of tachycardia‐induced cardiomyopathy with AF, in patients with and without SHD is unknown.

The mechanisms and pathophysiology of AF and HF share several risk factors and common pathophysiologic processes. Hypertension, smoking, obesity, diabetes, renal impairment, sleep apnoea, and CAD are all associated with an increased risk of developing both HF and AF, and each condition increases morbidity and mortality when associated with the other. All types of HF (HFpEF or HFrEF) are associated with an increase prevalence of AF.^294^, ^295^ There are no studies examining the role of monitoring to detect AF in asymptomatic patients with HF or the management of AF if detected. For patients with cardiac implantable electronic devices, remote monitoring is a tool for determining AF burden and is part of routine device follow‐up. In patients with HF, the risk of AF is increased by several mechanisms, remodelling of atrial structure and increased fibrosis, ectopy promoted by atrial stretch, increased spontaneous firing in the pulmonary veins and alterations in calcium current handling in the atrial muscle and sarcoplasmic reticulum calcium content.^290^

The loss of atrial systole in AF impairs LV filling and can result in left ventricular dilatation, decrease in myocardial blood flow and increase in LV wall stress and end‐diastolic pressure. Atrial fibrillation can decrease cardiac output by 25% particularly in patients with diastolic dysfunction. The mechanisms for reduction in cardiac output include loss of atrial contribution to ventricular filling, increased mitral regurgitation and decreased left ventricular filling time. The irregular and rapid ventricular contraction in AF can lead to LV dysfunction in an unknown percentage of patients and in some patients a tachycardia‐induced cardiomyopathy results.^291^ The irregular ventricular response also compromises ventricular performance through changes in calcium handling and reduced expression of Serca and phospholamban phosphorylation. Management can vary widely according to presentation and should be individualized since treatments shown to be effective in one or other condition alone, may give rise to safety or efficacy issues in an individual patient. Several recent trials have suggested a preferential role for primary catheter ablation of AF in select AF patients with HF compared to medical therapy alone.^296^, ^297^, ^298^ Treatment of AF by either rate or rhythm control may reverse the cardiomyopathy and improve clinical HF substantially in selected patients.

### 4.4 Risk for death in atrial fibrillation patients

**Table:** 

Risk for death in AF patients (including risk for SCD)	Class	References
Clinical characteristics of the patient including presence of advanced age, cognitive dysfunction or dementia, diabetes mellitus, hypertension, prior stroke, vascular disease, and HF should be used as important risk markers of higher mortality in patients with AF.	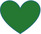	299, 300

**FIGURE 5:**
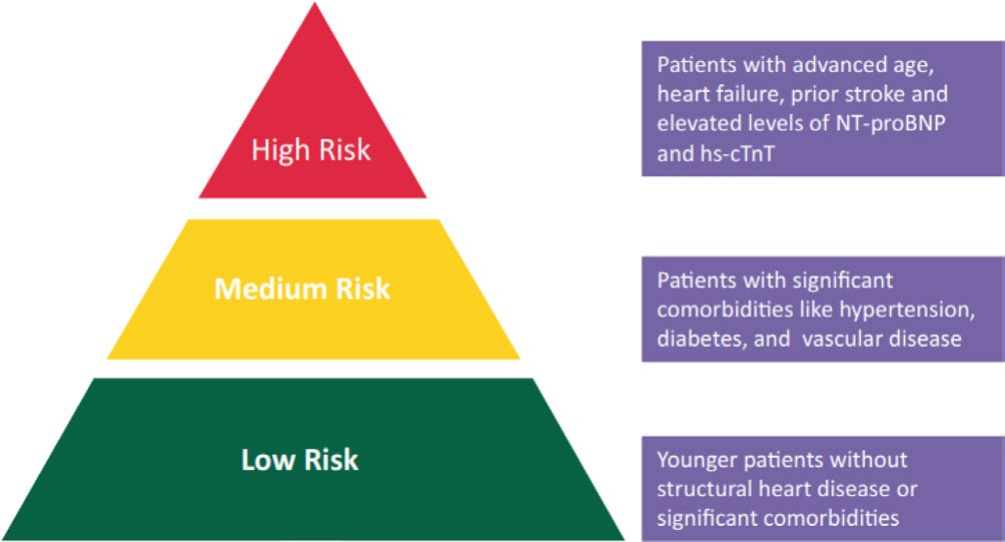
Mortality risk in patients with atrial fibrillation.

Atrial fibrillation is associated with 1.5‐ to 2‐fold higher risk of all‐cause mortality which may result from stroke, HF, or SCD.^279^ Of the mortality associated with AF, only 1 in 10 deaths are stroke, and >7 out of 10 are cardiovascular.^301^ A multipronged strategy incorporating stroke prevention, better symptom control, and cardiovascular risk optimization is associated with improved outcomes, including a reduction in mortality.^3^, ^4^ Females with AF have slightly higher mortality compared to male patients. Ethnic or racial differences exist in mortality risk, with one study showing highest risk in African Americans among all racial/ethnic groups.^302^ Also, presence of comorbidities increases the risk compared with ‘lone’ AF. Advanced age, renal failure, pulmonary disease, and HF have been found to be most important risk factors for higher mortality in AF (Figure 5).^299^, ^300^

Numerous risk scores have been designed to assess the mortality risk in AF. The CHA_2_DS_2_‐VASc score was designed to assess stroke risk, but given it is a cluster of common risk factors for cardiovascular mortality also predicts mortality risk.^303^ More complex clinical risk scores designed to predict mortality, such as an integrated GARFIELD‐AF risk tool, statistically improves mortality prediction, being superior to the CHA_2_DS_2_‐VASc score.^304^ All clinical risk scores only have modest predictive value (c‐indexes 0.6‐0.7) but can always be statistically improved by the inclusion of cardiac biomarkers, such as NT‐proBNP and hs‐TnT. Both biomarkers (and others) have been found to be independently associated with increased midterm mortality in AF patients presenting to emergency room.^305^ Indeed, risk scores incorporating biomarkers have been proposed, such as the ABC‐death risk score, which utilizes age, biomarkers, and clinical history. The ABC‐death score achieved a c‐index of 0.74 (95% confidence interval [CI] 0.72‐0.76), while the CHA_2_DS_2_‐VASc score achieved a c‐index of 0.58 (95% CI 0.56‐0.61).^306^ However, the clinical usefulness of any risk‐prediction score for mortality has not been established, and further validation studies are needed. Indeed, many risk factors or biomarkers are based on measurements done at baseline, and follow‐up events occur many years later. Cardiovascular risk is not static but changing with increasing age and incident risk factor(s), thus repeat risk re‐assessment is more appropriate given that a change in risk scores is more highly predictive of adverse outcomes.

Importantly, many biomarkers are non‐specific, more likely reflecting a patient with significant comorbidities and significant underlying heart disease, and are predictive of various endpoints apart from death, including stroke, heart failure, etc.^307^, ^309^ Indeed, biomarker‐based scores like ABC‐death were derived from a highly selected clinical trial cohort which was anticoagulated, and values were determined at study entry (baseline). Many biomarkers also have a diurnal variation and inter/intra laboratory variability and are predictive of non‐cardiovascular outcomes. Real‐world studies investigating the usefulness of sequential addition of biomarkers have shown limited value over conventional clinical risk scores.^10^, ^310^, ^311^ Thus, statistically significant improved prediction should not be confused with clinically improved risk prediction. A balance is therefore needed between (statistically) improved risk prediction and simplicity or practicality for everyday clinical use in busy clinical settings. In summary, any novel biomarker (or biomarker‐based scores) would need to be validated in large non‐anticoagulated cohorts. This is the starting point of risk stratification with the newly diagnosed AF patient in any patient care pathway, and be simple, practical and adequately validated to account for the dynamic nature of risk factors and changes in drug therapies (including the use of antithrombotic drugs) over time.

Stroke resulting from AF has significant medium‐term mortality, which can be as high as 30.5% at 1 year.^311^ An 8‐point GPS‐GF score utilizing variables including Glasgow Coma Scale, pneumonia, midline shift on brain images, blood glucose, and female sex has been developed and was found useful to predict 30‐day mortality in patients with AF‐related stroke.^312^

Spontaneous AF is associated with an increased risk of SCD in patients with Wolff–Parkinson–White (WPW) syndrome, HCM, and channelopathies such as Brugada syndrome.^279^ Several recent studies on HF and LVH and those on the general population have reported that AF is linked to an increased risk of SCD.^314^, ^315^, ^316^ Mechanisms for SCD due to AF are well understood for WPW syndrome or HCM, but are unclear regarding other cardiac disorders. A meta‐analysis demonstrated a significant association between AF and SCD in the general population as well as in patients with CAD, congestive HF, HCM, Brugada syndrome, and implanted rhythm devices.^316^ In a nationwide cohort study from Taiwan, 352 656 patients were identified. Among AF patients, age ≥75 years, congestive heart failure, hypertension, diabetes mellitus, previous stroke/TIA, vascular diseases, chronic kidney disease, and chronic obstructive pulmonary disease were important risk factors for SCD or ventricular arrhythmias.^317^ A recent study suggested that optimal pharmacological treatment, in addition to anticoagulant therapy, can reduce SCD rates in patients with AF.^318^ Since pharmacological rhythm control has so far been relatively ineffective in preventing SCD in AF patients with low LVEF,^319^ catheter ablation may be more appropriate for improving prognosis in patients with AF.^297^ To assess the risk of SCD in patients with AF, recognizing the presence of CAD, HF, LVH/HCM, pre‐excitation, Brugada syndrome, and implanted rhythm devices is crucial. Examinations including 12‐lead ECG, echocardiography, and other imaging modalities such as cardiac MRI are useful for detecting various cardiac disorders. Electrophysiological testing is useful for identifying risks in patients with WPW syndrome and paroxysmal AF.

### 4.5 Risk of adverse outcomes in patients treated with catheter ablation

**Table:** 

Risk of adverse outcomes in patients treated with catheter ablation	Class	References
Patients that undergo an AF ablation should be monitored closely in the first 30 days after the procedure due to a higher risk of neurological, gastrointestinal, cardiovascular, vascular and peripheral complications	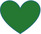	321, 322, 323, 324, 325, 326
Wolff–Parkinson–White syndrome patients following radiofrequency ablation may benefit from additional follow‐up due to a persistent elevated risk of developing AF compared to the general population	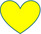	296, 327

Radiofrequency (RF) ablation has emerged as a main therapeutic option for treatment of AF patients since 1998 after the observation that AF mostly initiates from arrhythmogenic triggers in muscular sleeves in the pulmonary veins.^327^ There is abundant evidence that AF ablation is an effective method for AF suppression leading to significant reduction of AF episodes and burden accompanied by substantial improvement in symptoms and quality of life if performed in symptomatic patients. For this reason, AF ablation is mainly recommended by current guidelines as a method for symptom improvement in symptomatic AF patients.^279^

#### 4.5.1 Post‐ablation atrial fibrillation recurrence

Post‐ablation AF recurrence is one of the most important and frequent adverse outcomes, which occurs in 30%‐50% of cases.^329^, ^330^ In fact, although the acute success rate of AF catheter ablation seems high, achieving a durable treatment efficacy has remained a main challenge.^279^, ^330^ Different factors including female gender, older age, traditional cardiac risk factors, left ventricular dysfunction, increased epicardial adipose tissue, myocardial fibrosis, and atrial enlargement have been proposed as possible predictors of post‐ablation AF recurrence.^331^, ^332^, ^333^ Moreover, diverse AF recurrence risk‐prediction scores, including APPLE, ALARMEc, ATLAS, BASE‐AF2, CAAP‐AF, DR‐FLASH, and MB‐LATER have been introduced; however, their integration into the daily clinical practice needs further support by healthcare systems.^334^, ^335^, ^336^, ^337^, ^338^, ^339^, ^340^, ^341^, ^342^, ^343^

#### 4.5.2 Other adverse outcomes

Apart from AF recurrence, according to the available real‐world data, around 5%‐15% of patients undergoing AF catheter ablation experience complications, mainly during the index hospitalization and early in the post‐procedure course.^321^, ^322^, ^323^, ^324^, ^325^, ^326^ A variety of complications, including neurological, gastrointestinal, cardiovascular, vascular and peripheral, as well as pulmonary complications have been reported to occur after ablation procedures.^321^, ^322^, ^323^, ^324^, ^325^, ^326^, ^344^, ^345^, ^346^, ^347^, ^348^ Although different modifiable factors such as metabolic syndrome, hypertension, alcohol consumption, sleep apnoea, and obesity have been proposed to be related with arrhythmia‐free survival after catheter ablation,^349^, ^350^, ^351^, ^352^ their impact on the ablation adverse outcomes is not clear yet, and requires further investigations.

##### Mortality and morbidity

The impact of the ablation on hard clinical endpoints is much less evident. Previous findings from observational studies indicated a positive effect of the procedure on mortality and morbidity.^352^ These, however, were not confirmed in the recent large randomized Catheter Ablation vs Antiarrhythmic Drug Therapy for Atrial Fibrillation Trial (CABANA) that had as primary endpoint a composite of death, disabling stroke, serious bleeding, or cardiac arrest.^296^ In contrast, positive effects on hard clinical endpoints including mortality have been reported in patients with HF. In the CASTLE‐AF trial, patients with impaired LVEF <35% and previous ICD implantation who were treated with ablation therapy had a lower rate of death from any cause or hospitalization for worsening HF compared to patients undergoing medical treatment.^297^

##### Stroke

Regarding the impact of AF ablation on stroke and in particular the validity of stroke risk schemes for stroke risk stratification after ablation, observational data suggest a reduced stroke risk after AF ablation and a possibly safe termination of anticoagulation, at least in selected patients.^354^, ^355^ Conclusive evidence is expected from ongoing randomized trials as the Optimal Anticoagulation for Higher Risk Patients Post‐Catheter Ablation for Atrial Fibrillation Trial (OCEAN) (NCT02168829) and the Prevention of Silent Cerebral Thromboembolism by Oral Anticoagulation with Dabigatran After Pulmonary Vein Isolation for Atrial Fibrillation (ODIn‐AF) trial (NCT02067182). Until now, one randomized trial showed that ablation therapy for AF in patients with impaired LVEF was associated with significantly lower rate of death from any cause and worsening HF.^274^ Subgroup recommendations may change after the completion of trials studying the effect of ablation on stroke and the need for anticoagulation. Particularly in HF patients, it remains to be seen in which subgroups of patients the data indicating mortality reduction after AF ablation are applicable.

#### 4.5.3 Catheter ablation in Wolff–Parkinson–White patients

Careful attention must be given in WPW patients who underwent RF ablation, as it was demonstrated that they had an increased risk of AF at follow‐up when compared to general population, though an increased risk of death was not reported.^297^, ^327^

### 4.6 Risk of adverse outcomes in patients treated with surgical Maze

The surgical Cox–Maze operation was introduced in 1987 to treat patients with refractory AF.^355^ This surgical approach carries more risk of complications than the catheter ablation procedure, and is suitable for selected patients only. In this setting, we can observe three different case‐scenarios.

#### 4.6.1 Atrial fibrillation surgery

A simplification of the Cox–Maze procedure was proposed by replacing the ‘cut and sew’ lesions by different ablation devices and minimally invasive access.^356^ In the recent years, bipolar RF clamping devices guided on a beating heart, by thoracoscopic epicardial approaches have been introduced.^277^, ^358^ This evolution has allowed the implementation of this surgery for stand‐alone persistent and long‐standing persistent AF ablation, after an ineffective antiarrhythmic drug treatment or a previous endocardial ablation failure with a IIa (Level of Evidence B) indication.^278^ On another hand, this invasive approach carries some potential risks that need to be anticipated and discussed. Ideally, this step should involve an arrhythmia team in order to discuss the risk–benefit balance of the procedure on a case by case basis.^358^

#### 4.6.2 Surgical Maze in patients with concomitant heart surgery

An AF surgical ablation procedure is reasonable for selected patients with AF undergoing cardiac surgery for other indications.^279^ In patients that may receive a concomitant Maze procedure, a shared decision‐making strategy should be used with an AF heart team to make the best decision available for the patient and their heart condition.^358^ Mortality or major morbidity was not affected by concomitant AF surgery (adjusted odds ratio [OR] 1.00; 95% CI 0.83‐1.20), but pacemaker implantation was more frequent (adjusted OR 1.26; 95% CI 1.07‐1.49).^359^ Stiff LA syndrome was also reported after surgical Maze procedure, presenting with dyspnoea, pulmonary hypertension, and elevated left ventricular end‐diastolic pressure attributed to reduced LA compliance.^360^

Predictors of AF recurrence after surgery include left atrial dilatation, older age, over 10‐year history of AF, and non‐paroxysmal AF.^362^, ^363^, ^364^, ^365^, ^366^

#### 4.6.3 Stand‐alone surgical Maze

A stand‐alone AF surgical ablation procedure may be reasonable for selected patients with highly symptomatic AF not well managed with other approaches (e.g. after a failed catheter ablation, longstanding AF, dilated left atrium).^366^ After Cox–Maze IV stand‐alone procedure, overall operatory mortality was 1%‐1.8%, overall complication rate was 10%, 8% required pacemaker placement, and 12‐month freedom from atrial tachyarrhythmias was 89% (78% without antiarrhythmic drugs).^368^, ^369^

#### 4.6.4 Left atrial appendage exclusion or removal during surgical Maze

The prospective randomized trial comparing the efficacy and safety of LAA exclusion or removal with surgical Maze procedure is lacking. However, epicardial LAA Atriclip occlusion showed a high rate of complete left atrial appendage occlusion and reduces the incidence of stroke in patients with AF undergoing cardiac surgery.^282^ After surgical occlusion or exclusion of the LAA, it is recommended to continue anticoagulation in at‐risk patients with AF for stroke prevention.^279^ If surgical LAA ligation fails or is incomplete, stroke rates are significantly increased compared to patients with complete closure.^286^

## 5 HOW TO ASSESS RISK FOR VENTRICULAR TACHYARRHYTHMIA IN SPECIFIC POPULATIONS

### 5.1 Patients with ischaemic heart disease

Ventricular tachyarrhythmia/ventricular fibrillation events are closely related to the risk of SCD in patients with ICM. For this reason, the risk of VT/VF is commonly used as a surrogate for the risk of SCD. In addition, in ischaemic cardiomyopathy (ICM), myocardial ischaemia is the most common trigger for VF and SCD.

For primary prevention, our current approach to SCD risk stratification relies mainly on the evaluation of LVEF: values below 30%‐35% allow the identification of ICD candidates, who are at highest relative risk of SCD. On the other hand, patients with a LVEF >35% account for the highest absolute number of SCDs.^369^ For this reason, many researchers emphasize that EF is an inadequate marker for detecting patients who are at high risk for SCD despite having a normal or sub‐normal EF. It seems also to have very limited value to identify amongst patients with a low LVEF those who will benefit the most from an ICD. In other words, many patients with EF ≤35% are unnecessarily implanted with an ICD for primary prevention, while some others, having a EF >35% and a high risk of VT/VF, are not protected. In this setting, new markers are needed to optimize screening and patient selection for ICD implantation. For secondary prevention, SCD risk is significantly higher, and risk stratification is certainly more standardized.^61^, ^74^

#### 5.1.1 Secondary prevention of ventricular tachyarrhythmia/ventricular fibrillation in patients with ICM

**Table:** 

Secondary prevention of VT/VF in patients with ICM	Class	References
ICM substrate and ischaemic triggers for VT/VF must be evaluated when appropriate (coronary angiogram, functional ischaemic evaluation by nuclear scan, stress‐echocardiography, or MRI)	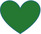	54, 70, 71
Cardiac MRI with a LGE can be considered in order to evaluate arrhythmogenic substrate including myocardial scarring to include in risk assessment, and guide a possible VT ablation procedure. This investigation should be preferably performed before ICD implantation to avoid artefacts due to the presence of an implanted device	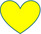	^370^

For more than 20 years, patients with a history of sustained VT/VF have been recognized to be at high risk of recurrence.^371^ Nowadays, these patients are given a Class I (Level of Evidence A) indication for ICD implantation.^70^ For this reason, the practical usefulness of VT/VF recurrence risk assessment is questionable, as additional testing is likely not going to influence decision pathways (i.e. catheter ablation or antiarrhythmic drug therapy as an alternative to ICD implantation), and patient outcomes in a secondary prevention setting.

#### 5.1.2 Primary prevention of ventricular tachyarrhythmia/ventricular fibrillation in patients with ICM and a left ventricular ejection fraction ≤35%

**Table:** 

Primary prevention of VT/VF in patients with ICM and LVEF ≤35%	Class	References
ICM substrate and ischaemic triggers for VT/VF must be evaluated when appropriate (coronary angiogram, functional ischaemic evaluation by nuclear scan, stress‐echocardiography or MRI)	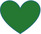	54, 70, 71
Cardiac MRI with a LGE can be considered in order to evaluate arrhythmogenic substrate including myocardial scarring to include in risk assessment and guide a possible VT ablation procedure. This investigation should be preferably performed before ICD implantation to avoid artefacts due to the presence of an implanted device	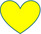	^370^

Patients presenting with ICM, in NYHA Class II‐III, with EF ≤35% after 3 months of optimized heart failure pharmacological treatment, are given a Class I/A indication for ICD implantation for the primary prevention of SCD.^70^ Nonetheless, it is widely recognized that only a small subgroup of these patients will present with VT/VF during follow‐up, and consequently will benefit from the device. A better risk stratification of these patients would be crucial to help identify those who would indeed benefit from an ICD. Most of the numerous investigations assessed in this setting, like programmed ventricular stimulation (PVS), heart rate variability (HRV), late ventricular potentials (LVP), baroreflex sensitivity, QT interval dispersion, T‐wave alternans, and heart rate turbulence have been largely abandoned because none of them have influenced routine clinical practice.^46^, ^73^, ^373^, ^374^ However, some of these explorations, like T‐wave alternans, have shown some value for SCD prediction in ICM patients.^42^ It is still uncertain whether biochemical markers as B‐type natriuretic peptide and N‐terminal pro‐BNP will prove useful in assessing risk for VT/VF. Cardiac MRI with LGE should also help to improve VT/VF and SCD risk stratification by analysing cardiac structure and myocardial scarring.^376^ Finally, a recent randomized trial suggests that assessment for hibernating myocardium performed routinely is of no use to decrease the risk of SCD.^68^

#### 5.1.3 Primary prevention of ventricular tachyarrhythmia/ventricular fibrillation in patients with ICM and left ventricular ejection fraction >35%

**Table:** 

Primary prevention of VT/VF in patients with ICM and LVEF >35%	Class	References
ICM substrate and ischaemic triggers for VT/VF must be evaluated when appropriate (coronary angiogram, functional ischaemic evaluation by nuclear scan, stress‐echocardiography or MRI)	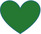	54, 70, 71
EPS and non‐sustained VT evaluation could be considered to improve VT/VF risk stratification in patients with relatively preserved LVEF, particularly in the convalescent phase (first 2 months) after an acute coronary syndrome	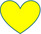	313, 375, 376
Heart rate variability (HRV), LVP, baroreflex sensitivity, QT‐interval dispersion, T‐wave alternans and heart rate turbulence have not been evaluated adequately in this population for generalized use	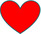	73, 373, 374

This group of patients should be the priority for VT/VF risk assessment: in absolute numbers, it represents by far the highest number of those at risk of VT/VF and SCD.^369^ In addition, these patients are currently non‐protected, as they are not targeted for ICD implantation in guidelines, due to their LVEF value.^70^ In this setting, MRI with LGE could be an option to better understand the diagnosis, prediction, and treatment of VT/VF.^370^ This investigation could possibly help improve VT/VF and SCD risk stratification by analysing cardiac structure and myocardial scarring, particularly when EF is relatively preserved. In this setting, a large prospective trial documenting that treatment guided by MRI‐based risk stratification improves outcomes in this patient group is still very much expected.^376^

Otherwise, the MUSTT Trial suggested the value of EPS for improving the SCD risk stratification, in the subgroup of ICM patients with a residual EF comprised between 30 and 40%.^377^

In addition, other non‐invasive investigations like tissue Doppler Imaging (TDI) seem also to be of potential value in predicting VT/VF in ICM. Late diastolic velocity assessed by TDI, particularly when detected in the inferior myocardial wall, seems to be a sensitive marker of future VT/VF.^374^ Finally, it is well known that non‐sustained ventricular tachycardia (NSVT) is a marker of increased risk of VT/VF and arrhythmic death. During the convalescent phase after an acute coronary syndrome, NSVT seems to be associated with an increased risk of cardiovascular death, most marked within the first 2 months after detection.^375^ The use of such investigations could help to detect those patients at higher risk of VT/VF, more particularly during the early phase after an acute coronary event. Specific measures like prolonged monitoring or use of wearable cardiac defibrillator could be undertaken on an individual patient‐case basis. However, more solid data are needed to support such recommendations broadly.

### 5.2 Patients with non‐ischaemic heart failure

**Table:** 

Patients with non‐ischaemic heart failure	Class	References
MRI may be considered for further risk stratification of sudden death in patients with non‐ischaemic cardiomyopathy who do not otherwise meet an indication for ICD implantation	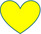	^378^
EPS may be considered for further risk stratification of sudden death in selected patients with non‐ischaemic cardiomyopathy who do not otherwise meet an indication for ICD implantation	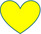	^378^

Patients with non‐ischaemic HF represent a broad and diverse group of patients including those with progressive and infiltrative forms of cardiomyopathies. For this reason, the risk of developing VT in non‐ischaemic HF is difficult to accurately predict in this group of patients. Subsequent sections in this document will address specific conditions that have unique risk profiles including inflammatory cardiomyopathies, congenital heart disease, arrhythmogenic cardiomyopathy, and Chagas’ disease.

Prior investigations into identification of the risk of developing VT in non‐ischaemic cardiomyopathy focused on the risk of SCD and the role of the implanted defibrillator for primary prevention. The DANISH trial^61^ reported no survival benefit from prophylactic ICD implantation in the overall cohort. Implantable cardioverter‐defibrillator reduced SCD to half, and subgroup analysis showed that in patients younger than 68 years, survival was prolonged with an ICD. Although pooled analysis of the five primary prevention trials (DEFINITE, SCD‐HeFT, CAT, AMIOVIRT, COMPANION, and DANISH; n = 2970) revealed that ICD therapy was superior to medical therapy in patients with non‐ischaemic cardiomyopathy with decreased cardiac function, these trials were judged globally negative.^379^

In a limited number of studies outside of these clinical trials, the role of EPS or non‐invasive programmed stimulation has revealed inconsistent results.^378^ More recently, the role of cardiac MRI for definition of scar and potential substrate has emerged as a powerful risk stratification tool in observational studies.^49^, ^380^, ^381^ Genetic testing is also useful in patients with decreased cardiac function with conduction disturbance (i.e. LMNA mutations).

In summary, non‐ischaemic HF includes a diverse group of patients with reduced ventricular function due to cardiomyopathies from different aetiologies, and at high risk for VT. Reduced cardiac function remains a powerful predictor of VT and appropriate ICD therapy in these patients as a primary prevention. Cardiac MRI and EP testing shows promise in some subsets. Further characterization based on the type of cardiomyopathy leading to HF shows the most promise for accurate assessment of VT risk.

### 5.3 Patients with inflammatory cardiomyopathies

**Table:** 

Patients with inflammatory cardiomyopathies	Class	References
In patients with non‐ischaemic heart disease who present with ventricular arrhythmias, use of cardiac MRI or cardiac PET can help delineate aetiology of non‐ischaemic cardiomyopathy, initiate aetiology‐driven treatment, and evaluate prognosis.	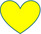	52, 53, 380

Inflammatory cardiomyopathies encompass a broad spectrum of disorders characterized by myocardial inflammation as the primary cause of cardiac dysfunction. This includes viral myocarditis (commonest cause), cardiac sarcoidosis, giant cell myocarditis, autoimmune myocarditis associated with underlying connective tissue diseases, eosinophilic cardiomyopathies, and Chagas disease (addressed in a separate chapter).

In patients who present with ventricular arrhythmias and diagnosed with non‐ischaemic cardiomyopathy, the incidence of inflammatory cardiomyopathy may be as high as 50%.^382^ Therefore, it is important to consider inflammatory cardiomyopathies as an underlying cause, given that these conditions may benefit from specific aetiology‐driven treatments. Infectious causes of myocarditis include viral (e.g. parvovirus B19 and human herpes virus 6 genomes that predominate in inflammatory cardiomyopathies, other cardiotropic viruses include enteroviruses, adenoviruses, hepatitis C, and human immunodeficiency viruses) and uncommonly bacterial and other causes depending on the geographical area and immunosuppression status. Myocarditis associated with connective tissue and autoimmune diseases encompass systemic lupus erythematosus, scleroderma, rheumatoid arthritis, dermatomyositis, polymyositis, cardiac sarcoidosis and giant cell myocarditis. Drug reactions may also cause hypersensitivity myocarditis.^382^, ^383^ In cases of an established cause of inflammatory cardiomyopathy, the focus should be on treating the underlying inflammatory condition. In the case of cardiac sarcoidosis, retrospective series have shown that specific treatment with immunosuppressive therapy can increase VT free survival.^52^

Cardiac MRI scan is the gold standard for diagnosing myocarditis and inflammatory cardiomyopathies. Oedema, hyperaemia, and LGE form the diagnosis of acute myocarditis. Further diagnostic information is gleaned from T1 and T2 mapping techniques. Although no specific LGE pattern on MRI is diagnostic of cardiac sarcoidosis, LGE is most often observed in basal segments, particularly of the septum and lateral wall, and usually in the mid‐myocardium and epicardium of the myocardium^384^, ^385^, ^386^

The presence of LGE significantly increases risk of adverse cardiac events. The presence of LGE on cardiac MRI increased the risk of ventricular arrhythmias and death by greater than 20 fold in patients with EF >35% and extracardiac sarcoidosis compared to sarcoid patients without evidence of LGE on MRI, and the burden of LGE was associated with higher rates of death/VT.^387^ In a meta‐analysis of 155 patients with systemic sarcoidosis who underwent cardiac MRI for work‐up of cardiac sarcoidosis, the presence of LGE was associated with hazard ratio of 31.6 for death, aborted SCD, or appropriate ICD discharge and provided superior prognostic information as to compared to other clinical and functional characteristics, including LVEF.^51^

In addition, the distribution of LGE confers important prognostic information, with mid‐wall anteroseptal LGE representing a more malignant form compared to a sub‐epicardial inferolateral wall LGE pattern.^388^, ^389^ Inflammatory biomarkers, such as C‐reactive protein, are typically lower in this group with septal LGE, but biomarkers of myocardial damage such as troponin are typically higher, suggestive of a subset with less inflammation but greater myocardial injury. F‐fluorodeoxyglucose (FDG)‐PET is advantageous for detecting active inflammation in cardiac sarcoidosis, and a mismatch of FDG and perfusion and involvement of the right ventricle predicts adverse cardiac events and ventricular arrhythmias, respectively.^53^ Endomyocardial biopsy is performed in cases where a histological diagnosis is required to confirm cardiac sarcoidosis or giant cell myocarditis, with its yield enhanced by electrogram guidance. Active viral genomes may also be identified by biopsy, which can differ significantly from peripheral serological tests.^383^, ^390^

Little data exist on how to assess risk of VT/VF in inflammatory cardiomyopathies. Besides EF, which is used for all non‐ischaemic aetiologies, no randomized studies have evaluated other parameters or even EF as a predictor of VT in different inflammatory cardiomyopathies. In particular, certain inflammatory cardiomyopathies may carry higher risk than others (sarcoidosis vs. viral myocarditis). Risk of ICD therapy may be as high as 15% per year in biopsy proven cardiac sarcoidosis patients.^391^ Although randomized data on use of higher EF in these patient populations is lacking, given risk of VT noted in retrospective studies, use of MRI and cardiac PET to evaluate aetiology of non‐ischaemic heart disease is warranted, and treatment of inflammation to reduce risk of VT is advised. Furthermore, cardiac PET and MRI can be used to assess for recurrent inflammation or progression of disease on treatment.

### 5.4 Patients with congenital heart disease

**Table:** 

Risk for ventricular arrhythmias in patients with congenital heart disease	Class	References
In the paediatric patient with CHD, ventricular overload, surgical scars and patches or baffles, ventricular dysfunction, and previous conduction defects are recognized risk factors for VT.	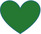	392, 393, 394
In adult patients with CHD, older age at surgery, poor haemodynamic status, and prolonged QRS represent the most common risk factors for ventricular arrhythmias.	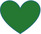	393, 394, 395
In adult patients with CHD, VTs are mainly observed after correction of tetralogy of Fallot (TOF) and left ventricular outflow tract defects.	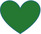	395, 396, 397
In patients with TOF, residual haemodynamic lesions and ventricular dysfunction represent the most important risk factors for VT or SCD.	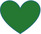	395, 396, 397
In patients with TOF, frequent PVCs, QRS >180 ms, palliative systemic to pulmonary shunts, syncope, atrial tachycardia, decreased LVEF, dilated right ventricle, severe pulmonary stenosis or regurgitation, are risk factors for sustained VT.	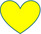	395, 396, 397

Ventricular arrhythmias in patients with congenital heart disease (CHD) may be observed in two different groups: the paediatric age group and adults with repaired congenital defects group.^398^ In the paediatric age, life‐threatening VT is rare both prior to and after surgery. Ventricular tachyarrhythmia is seen in only 1.8% of children undergoing an EPS,^392^ is usually associated with structurally normal heart and most frequently comes from the right outflow tract and left outflow tract and sinuses of Valsalva.

In paediatric patients with CHD, the haemodynamic and electrophysiologic factors related to each disease state and associated therapeutic interventions play an important role in the development of VT, with ventricular overload, surgical scars and patches, baffles and conducts, ventricular dysfunction, and previous conduction defects among the most relevant.^393^ In the early post‐operative stage, Van Hare *et al*. reported only 3 patients with VT out of 580 undergoing paediatric surgery and the most important risk factor was the surgical procedure.^392^ Sustained VT may arise in the setting of myocardial ischaemia or infarction and may be facilitated by disruption of the ventricular myocardium caused by scar due to ventriculotomy, fibrotic tissue, or ventricular dilatation.^394^

In adult patients with CHD, VTs are mainly observed after correction of tetralogy of Fallot (TOF) and left ventricular outflow tract defects but may also arise in other defects as transposition of the great arteries with atrial switch, univentricular hearts, double‐outlet RV, and ventricular septal defects. Older age at surgery, poor haemodynamic status, and prolongation of the QRS represent the most common risk factors. In patients with TOF, the correlation of residual haemodynamic lesions and right ventricular dysfunction with risk of VT or SCD has been extensively established.^395^, ^396^ Potentially treatable residual haemodynamic problems, pulmonary hypertension, elevated end‐diastolic pressures, and reduced ventricular function should be treated as part of the arrhythmia management. Particularly in this group, frequent PVCs, QRS 180 milliseconds or more, palliative systemic to pulmonary shunts, syncope, atrial tachycardia, decreased LVEF, dilated right ventricle, severe pulmonary stenosis or regurgitation are risk factors for sustained VT, and inducible sustained VT correlates with increased risk of SCD.^397^, ^399^ EPS might be considered for risk assessment of VT/VF in this group of patients with high‐risk clinical characteristics and frequent ventricular arrhythmias.^328^

### 5.5 Patients with inherited arrhythmia diseases (inherited channelopathies and inherited structural diseases including arrhythmogenic right ventricular cardiomyopathy)

**Table:** 

Risk for ventricular arrhythmias in patients with inherited arrhythmia diseases	Class	References
Patients with primary inherited arrhythmia syndromes and cardiomyopathies should undergo risk stratification that integrates clinical presentation, family history, and non‐invasive diagnostic testing	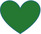	^400^
Select patients with primary inherited arrhythmia syndromes and cardiomyopathies may benefit from electrophysiologic testing to refine non‐invasive risk stratification	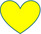	^401^

Patients with inherited arrhythmia disease are without doubt at increased risk for ventricular arrhythmias, including SCD. The extent to which this is pertinent and predictable is different for the various conditions.

The main primary inherited arrhythmia syndromes, i.e. the ‘channelopathies’ are LQTS, Brugada syndrome and CPVT.^402^ Patients that are symptomatic (syncope, cardiac arrest) at the time of presentation are at highest risk, with arrhythmic syncope representing a sentinel sign of risk, and resuscitated cardiac arrest reflecting the highest risk cohort.^97^ Despite major social impact on perceived risk, family history is not of major importance in all three diseases.

In LQTS, clearly defined disease‐specific risk factors are the extent of resting QT prolongation, documentation of arrhythmias and gene and even mutation specific associated risk.^403^ In CPVT, the extent of the arrhythmic response of an exercise test predicts events, including breakthrough symptoms on therapy.^404^ It follows that risk assessment requires a baseline ECG and an exercise test in both conditions, with potential value of ambulatory monitoring. Assessment should include asymptomatic patients often identified during family screening or after incidental unrelated medical evaluation.

In Brugada syndrome, there is uncontested agreement that symptomatic patients (arrhythmic syncope, cardiac arrest) are at high risk for SCD, requiring aggressive therapy with an ICD in most circumstances. Risk stratification in asymptomatic individuals with a spontaneous type 1 ECG is much less clear, involving a variety of ECG characteristics and potential value of programmed electrical stimulation (PES).^405^, ^406^ ECG parameters that have been associated with increased risk include QRS fragmentation, early repolarization, Brugada type changes in non‐anterior precordial leads and a positive signal‐averaged ECG. Programmed electrical stimulation with a non‐aggressive stimulation protocol may be of importance, although the risk of an inducible patient is only marginally different from a non‐inducible patient.^77^ In LQTS, CPVT, and Early Repolarization syndrome, PES is of no importance. The presence of a SCN5a mutation may contribute to risk in Brugada syndrome.^407^ Early repolarization syndrome, short‐coupled idiopathic VF (SCIVF), and SQTS are uncommon causes of cardiac arrest and sudden death. Though the early repolarization pattern conveys a small increase in risk, the only patients where the risk is substantive to consider intervention are those with prior cardiac arrest or syncope with a positive family history. There are no validated risk models in SQTS and SCIVF.

In the cardiomyopathies, i.e. the secondary inherited arrhythmia syndromes, risk stratification is also disease specific. In hypertrophic cardiomyopathy (HCM) septal thickness, the hallmark of the disease is an important contributor to risk. Other risk factors include left atrial dimension, left ventricular outflow tract gradient (all echocardiographic parameters), the presence of ventricular arrhythmias on ambulatory monitoring (Holter) or documentation otherwise, symptoms (i.e. unexplained syncope, palpitations associated with near syncope), demographic factors (age in particular), and family history. All these factors are included in the ESC risk score calculator,^408^ which is readily available in an online tool (http://www.doc2do.com/hcm/webHCM.html), and applied after standard imaging, exercise testing and ambulatory monitoring. Validation of the ESC risk calculator is not compelling, and consideration of imaging and exercise blood pressure response parameters have also been used in borderline cases. In inherited, i.e. non‐ischaemic, dilated cardiomyopathy, the genetic background is very important, with LMNA (Lamin A/C) and PLN (Phospolamban) leading to highly arrhythmic substrates.^123^, ^409^, ^410^, ^411^ Of course, reduced LVEF and the presence of ventricular arrhythmias during ambulatory monitoring are important risk factors as well. In arrhythmogenic right ventricular cardiomyopathy (ARVC) or arrhythmogenic ventricular cardiomyopathy (ACM), symptomatic arrhythmic events identify the patient at highest risk, and major risk factors include age, male sex, unexplained syncope, non‐sustained VT, number of anterior precordial leads with T wave inversion, and severe right or left ventricular dysfunction.^412^ Hence, as for the other cardiomyopathies, echocardiographic imaging, and Holter monitoring is required for risk assessment. In all cardiomyopathies, MRI is becoming increasingly important, in particular to show the presence of fibrosis (HCM, DCM, ACM) and assess left and right ventricular function. Genetic testing should be considered in any patient with a phenotype suggesting an inherited cardiomyopathy and in dilated cardiomyopathy with a suggestive family history or onset at an early age that is otherwise unexplained (i.e. not myocarditis, sarcoidosis etc.). Genetic testing is largely for diagnosis, and only informs risk when a high‐risk form of cardiomyopathy is diagnosed, such as PLN or LMNA.

### 5.6 Risk stratification in patients with arrhythmogenic cardiomyopathy, specified for arrhythmogenic right ventricular cardiomyopathy

**Table:** 

Risk stratification of ventricular arrhythmias in ARVC	Class	References
In patients with ARVC, history of aborted sudden death, sustained ventricular arrhythmias, and severe right and/or left ventricular dysfunction identify a high risk of cardiac death	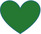	413, 414
In patients with ARVC, advice to not perform high‐level or endurance exercise should be given.	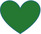	415, 416
Clinical factors including age, male sex, unexplained syncope, non‐sustained VT, number of anterior precordial leads with T wave inversion, and genetic mutation status can be used for prognostic stratification of patients with ARVC	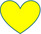	413, 414
In patients with confirmed ARVC, regular Holter monitoring and imaging for assessment of ventricular function may be useful.	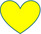	415, 416
A detailed history of exercise intensity and duration may be helpful in patients with ARVC as exercise level may represent a modified risk factor of adverse cardiovascular events and disease progression	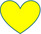	^417^

In arrhythmogenic right ventricular cardiomyopathy (ARVC or ), the most important features characterized as the high arrhythmic risk include the electric instability (i.e. sustained ventricular arrhythmia [VA]), genotype‐positive, extent of structural involvement, cardiac syncope, the presence of multiple mutations, and the history of competitive or endurance exercise.^413^, ^414^ In patients without prior VA, an available online prediction model, derived from the largest cohort of ARVC patients, using readily available clinical parameters was devised to estimate the risk of VA and to guide the decisions of ICD implantation as primary prevention (www.arvcrisk.com). 418

There is a dose‐dependent relationship between endurance exercise and the disease onset and progression in confirmed ARVC patients. Exercise restriction is recommended to prevent disease progression and SCD in confirmed ARVC patients with ICD^415^ and genotype‐positive relatives.^416^ In general, high‐level or endurance exercise is not recommended in confirmed ARVC patients or at risk.

Ambulatory ECG monitoring is crucial to detect the PVCs burden or the presence of non‐sustained VT, which also provide prognostic information in ARVC.^417^ All positive criteria of signal‐averaged ECG non‐invasively identifies the slow conduction of myocardium and has been proven for risk stratification in patients with suspicion or confirmed ARVC.^419^

Echocardiography and cardiac MRI provide accurate measurements of right ventricular global and regional dysfunction and right ventricular volume and regional/global ventricular function, as the important variable for assessment of right and left ventricular disease. The Task Force Criteria did not include cardiac MRI measures of right ventricular myocardial fat or LGE in order to risk stratify the ARVC.^420^ In summary, abnormal cardiac MRI was an independent predictor of clinical events with a cumulative effect of the abnormalities including morphology, wall motion, and fat/fibrosis in ARVC patients.^419^

An EPS may provide help distinguish ARVC from idiopathic right ventricular outflow tract (RVOT) VT. Additionally, positive inducibility on program ventricular simulation is not a perfect surrogate marker neither for ARVC diagnosis, nor the decision of ICD implantation.^413^, ^414^ EPS may be beneficial to identity patients that may benefit from ablation. In this setting, EPS with high‐dose isoproterenol may help differentiate patients with idiopathic VT or ventricular premature beats from those with ARVC.^421^ The positive inducibility of EPS can predict any ICD therapy, including VF, and can be an important parameter for risk stratification in patients with ARVC.

ARVC is considered to have desmosome dysfunction. Genetic causes of isolated or predominantly RV arrhythmia and structural abnormalities are most commonly associated with desmosomal gene variants. Positive genetic test contributes up to 50% of the diagnosis of ARVC, however, in confirmed ARVC patients, limited evidence of clinical actionable risk stratification or use of management of disease. Several gene variants have been reported in patients with left ventricular or biventricular arrhythmia. Left ventricular dysfunction is most often present in patients with ARVC with pathogenic variants in Lamin A/C, or variants in the PLN and TMEM43 genes, and followed by variants in DSP, DSG2/DSC2.^400^, ^401^, ^422^, ^423^

### 5.7 Patients with Chagas disease

**Table:** 

Patients with Chagas disease	Class	References
The Rassi score is useful in assessing risk of death in Chagas disease patients	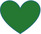	348, 349
In patients with syncope and a BBB, an invasive EPS is useful in assessing risk of sustained ventricular arrhythmias	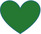	350, 351
When available, cardiac MRI with LGE should be considered to evaluate for arrhythmogenic substrate as part of a risk stratification strategy in those patients with cardiomyopathy	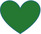	352, 353, 354, 355

Chagas disease is an infectious disease affecting 10 million people around the world and 100 million more are at risk of this infection. Due to migration, it is estimated 750 000 infected carriers live in the USA or Europe.^356^, ^424^ VA, especially sustained VT is closely related to high mortality, sudden death (SCD) happening in 17%‐50% of chronically ill patients.^356^ Based on the identification of different risk factors, Rassi *et al*. developed a mortality risk score (Table 3).^347^ Patients with HF, NYHA Class III/IV and NSVT on Holter and patients in NYHA Class I/II, with left ventricular dysfunction and NSVT on Holter are at the highest risk of death and should be regarded as candidates for aggressive therapeutic management.

**Table 3:** Rassi score

Risk factor	Points
NYHA Classes III or IV	5
Cardiomegaly (chest radiograph)	5
Segmental or global wall motion abnormality (2D echocardiogram)	3
Non‐sustained ventricular tachycardia (24‐h Holter)	3
Low QRS voltage (ECG)	2
Male sex	2

Total points	Total mortality (%)		Risk
	5 years	10 years	
0‐6	2	10	Low
7‐11	18	44	Intermediate
12‐20	63	84	High

Conversely, patients with an abnormal ECG (right or left bundle branch conduction disorders) but in NYHA Class I/II HF without left ventricular dysfunction or NSVT on Holter are at lower risk of death. These patients should be followed up annually or biannually. Between these two extremes, some patients are at intermediate risk and their treatment strategies should be individualized.

Sustained VT has been reported as the main cause of syncope in patients with non‐documented recurrent syncope and bundle branch block (BBB). In these cases, an EPS has been recommended for diagnosis elucidation.^349^ A finding of scar by LGE by cardiac MRI in patients with Chagas disease is considered a strong predictor of a combination of sustained VT and death.^357^

## 6 HOW TO ASSESS RISK FOR ADVERSE OUTCOMES IN PATIENTS WITH VENTRICULAR TACHYARRHYTHMIA

### 6.1 Risk for appropriate and inappropriate implantable cardioverter‐defibrillator therapies

ICD therapies are associated with an increase in mortality.^372^, ^425^, ^426^, ^427^ A single ICD shock is associated with a two‐ to five‐fold increase in mortality, and progressive heart failure has been reported the most common cause of mortality among these patients.^428^, ^429^, ^430^ ICD therapies are classified as appropriate, inappropriate, avoidable, and phantom.^372^, ^431^, ^432^ Approximately 12%‐17% of patients receive inappropriate ICD shocks.^425^, ^428^, ^429^, ^430^ Both appropriate and inappropriate shocks area associated with an increase in mortality and can significantly lower quality of life. Thus, identifying predictors of ICD therapies may improve quality of life and long‐term outcomes in patients with ICDs.

#### 6.1.1 Appropriate shock predictors

A previous episode of sustained VT correlates with high rate of appropriate shocks.^433^, ^434^, ^435^, ^436^ A higher risk of appropriate therapy was seen in a secondary prevention ICD group when compared with a primary prevention ICD group at 5‐year follow‐up, while the rate of inappropriate therapy was comparable.^437^ Several studies have shown male sex as an independent risk factor for appropriate ICD therapies.^438^ Women are 30%‐50% less likely to receive an appropriate shock,^439^, ^440^, ^441^, ^442^ and this difference is more pronounced among CRT‐D recipients.^443^, ^444^, ^445^ However, most of studies have shown similar mortality rates in both genders after ICD implantation.^438^, ^439^, ^440^, ^441^, ^442^, ^443^, ^444^, ^445^ AF is common in patients with left ventricular dysfunction; the prevalence can increase up to 50%. Worsening AF subtype increases the risk for both appropriate shocks and overall mortality.^446^, ^447^, ^448^, ^449^

Other risk factors implicated to increase the risk of appropriate shocks are diabetes,^446^, ^450^ elevated baseline NT‐proBNP and BNP,^451^ NSVT,^448^, ^452^ left atrial diameter,^446^, ^452^ and impaired renal function.^453^ Data from SCD‐HeFT and MADIT II trials have found a higher NYHA class, a lower LVEF, lack of use of beta‐blocker therapy and single‐chamber ICD as significant independent predictors for appropriate ICD shocks.^454^ Data from the Danish ICD Registry showed that LVEF <25% predicted an increased risks of both appropriate and inappropriate therapies.^455^

#### 6.1.2 Inappropriate shock predictors

The presence of supraventricular tachycardias, in particular AF, has been reported as the most common risk factor for inappropriate ICD shocks.^429^, ^447^, ^448^ Another risk factor associated with inappropriate shock is younger age.^451^, ^453^, ^454^ Inappropriate shocks secondary to AF/atrial flutter are associated with increased mortality while inappropriate shocks related to sinus tachycardia or non‐arrhythmic events like noise, artefact, and oversensing have shown similar survival as compared to those who do not receive a shock.^456^ Studies have failed to establish the superiority of dual‐chamber ICD over the single chamber in reducing inappropriate shocks.^457^, ^458^ The Danish ICD Registry showed a two‐fold increase in the risk of inappropriate shocks associated with a dual‐chamber ICD.^459^ Device technologies and programming, i.e. prolonged detection time, high rate programming, and better discrimination algorithms have markedly reduced the risk of inappropriate therapies.^372^, ^459^, ^460^

### 6.2 Risk for heart failure incidence and progression

**Table:** 

Risk for heart failure incidence and progression	Class	References
Periodic monitoring of PVC burden (every 6 months) and LVEF and dimensions are useful in patients with frequent, asymptomatic PVCs and a normal LVEF and dimensions	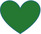	^461^
PVC burden exceeding 20% is associated with a higher risk of PVC‐related cardiomyopathy	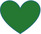	462, 463, 464
PVC burden lower than 10% is associated with a lower risk of PVC‐related cardiomyopathy	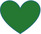	465, 466
In patients with PVC‐related cardiomyopathy, absence of LGE on cardiac MRI may be used to identify patients with a favourable prognosis of left ventricular systolic function recovery	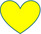	467, 468, 469

Tachycardia‐induced cardiomyopathy is a reversible cause of HF and impaired left ventricular function. Ventricular rhythms causing tachycardia‐induced cardiomyopathy include VT, fascicular tachycardia, PVCs, and even persistent rapid DDD pacing. Left ventricular systolic function improves or normalizes and symptoms resolve, when tachycardia is corrected or controlled with medication or pharmacologic or non‐pharmacologic rhythm control strategies.

Sustained monomorphic VT less commonly causes tachycardia‐induced cardiomyopathy as compared to supraventricular tachycardias, since sustained VT is most often associated with some form of structural heart disease. When VT does lead to tachycardia‐induced cardiomyopathy, it is by definition idiopathic and most commonly originates from the RVOT, left ventricular outflow tract, or coronary cusps. If these arrhythmias become persistent or high burden, they may cause reversible left ventricular dysfunction.^470^

A single centre series reported that 11% of patients who presented with frequent PVCs also had sustained monomorphic VT and 7% of those patients had tachycardia‐induced cardiomyopathy. The presence of repetitive monomorphic VT was a significant predictor of tachycardia‐induced cardiomyopathy development, particularly when it was the predominant arrhythmia on 24‐hours Holter monitoring.^464^

PVCs are very common and usually do not require treatment in the absence of symptoms. However, in the clinical setting of troublesome symptoms, or when PVCs trigger polymorphic VT or cause cardiomyopathy, proper treatment is critical. The concept of PVC‐induced cardiomyopathy was first proposed by Duffee et al,^463^ who observed a small group of patients with cardiomyopathy recover normal left ventricular function after pharmacological suppression of frequent PVCs.

Baman et al^462^ reported on 174 consecutive patients referred for PVC ablation, 54 of whom had depressed left ventricular function. The authors concluded that although PVC‐related cardiomyopathy may occur in patients with less PVCs, “in the presence of a PVC burden ≥24%, it may be helpful to suppress the PVCs by catheter ablation or drug therapy to avoid the development of cardiomyopathy.” However, Aki Lee *et al*., demonstrated a high rate of resolution of frequent PVCs among untreated patients with normal left ventricular function and minimal symptoms. A strategy of active surveillance is appropriate for the majority of patients with frequent idiopathic PVCs in association with preserved LVEF, owing to the low risk of developing left ventricular systolic dysfunction and the high rate of spontaneous resolution. Periodic monitoring of PVC burden and LVEF and dimensions can be useful in patients with frequent, asymptomatic PVCs and a normal LVEF and dimensions.^461^

It has become clear that comparative effectiveness trials are needed to understand what the best treatment approach is for patients with frequent PVCs and cardiomyopathy. A pilot multicentre study (PAPS: Prospective Assessment of PVC Suppression in Cardiomyopathy) is ongoing to better understand the prevalence of frequent PVCs and CM, and prove the feasibility of a large‐scale randomized clinical trial (not yet published).^471^

Several circumstances have been associated with PVC‐induced cardiomyopathy, including the PVC burden, asymptomatic status, duration of a high PVC burden, PVC QRS width >150 milliseconds, interpolated PVCs, epicardial origin, and male gender. However, no prospective longitudinal assessments have been conducted that definitively prove their causal relation to PVC‐induced cardiomyopathy.^472^

The diagnosis of tachycardia‐induced cardiomyopathy or PVC‐related cardiomyopathy can be challenging and the role of imaging modalities in the characterization of myocardial tissue as part of the diagnostic workup is limited.^467^ Cardiac MRI with LGE can accurately identify the presence and extent of myocardial scar and has become a first‐line non‐invasive imaging modality for the aetiologic assessment of primary cardiomyopathies and/or left ventricular systolic dysfunction, and could identify early stage of the structural heart disease.

### 6.3 Risk for death in ventricular tachyarrhythmia patients

**Table:** 

Risk for death in VT patients (including risk for SCD)	Class	References
Risk for SCD should be judged in each patient on a case‐by‐case basis and risk considered as a continuous variable rather than a dichotomized variable (high or low risk may change)	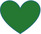	473, 474, 475
Individual risk assessment needs to be dynamic as the type and severity of risks can change over time (repeated measurements need to be made over time)	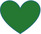	^476^
Risk assessment may include consideration of mode of death as the relative risk of non‐sudden, non‐cardiac death, sudden cardiac death, and non‐sudden cardiac death is influenced by aging and worsening cardiomyopathy and cardiovascular risk factors	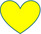	371, 477, 478

Risk prediction of death in VT patients has used numerous non‐invasive and invasive markers including: clinical markers, mode of initial clinical presentation (e.g. sustained stable monomorphic VT, ventricular flutter, or VF), biomarkers, ECG abnormalities (e.g. left bundle branch block), heart rate variability, signal‐averaged ECG, ambulatory ECG‐based frequency domain T wave, microvolt level‐T wave alternans, heart rate turbulence, heart rate deceleration, QT dispersion, cardiac autonomic function, echocardiographic evaluation of LVEF, left ventricular diameter, left ventricular mechanical dispersion by tissue Doppler, strain and velocity parameters to evaluate regional LV function, exercise testing to evaluate functional status, MRI to measure scar burden, and EPS to assess for inducibility of VT. Most of these tests and markers were applied to patients at risk of SCD and not patients who already have VT. Thus, their use for predicting death in a patient with VT is unknown.

The main sources of information about risk for SCD in patients with VT are from two studies from the era prior to widespread ICD use,^479^, ^480^ the control groups (patients who did not receive ICDs) in the primary prevention ICD studies (MUSTT, MADIT, MADIT II, SCD‐HeFT, DANISH, DEFINITE, CABage‐PATCH, IRIS, DINAMIT) as well as analysis of large data samples from registries since ICD approval from Europe, Canada, and the USA.^70^, ^481^, ^482^ These data have been extensively reviewed to better characterize which variables predict the development of SCD and death in high‐risk patients. Data from secondary prevention studies (AVID, CIDS, CASH) provide additional information about risk of death in patients who have had VT. Another source of information is the International VT Ablation Center Collaborative Study Group which analysed a large group of patients with VT (approximately 2000 patients from 12 international sites) undergoing catheter ablation.^483^ Finally, a third useful source of data is the Seattle Heart Failure model developed by Wayne Levy and his colleagues who analysed data from a large sample of heart failure patients to predict risk of death and SCD as well as create a model for predicting benefit from ICD therapy.^484^ This model has been prospectively validated among five additional study cohorts of almost 10 000 heart failure patients. It is important to recognize that the causes of death can change over time. For example, the risk of death in a patient with post‐MI VT may be largely due to mechanical problems (VSD, mitral regurgitation, heart failure) in the first several weeks to months after MI and then 3‐6 months later the risk of arrhythmic death may be much higher due to matured scar‐mediated substrate.

Based on these studies, the risk factors for death in VT patients include increasing NYHA class, old age, female gender, electrical storm, frailty, diabetes mellitus, AF, chronic kidney disease, chronic obstructive lung disease, peripheral arterial disease, advanced HF, non‐ischaemic cardiomyopathy, lower EF, multiple different VT morphologies, use of haemodynamic support devices during VT ablation, and poor functional status. These risk factors can be divided into risk factors related to non‐cardiac disease (e.g. renal function, diabetes, COPD, peripheral arterial disease) which are powerful and determine mortality, and cardiac risk factors (ischaemic vs. non‐ischaemic aetiology, multiple morphologies of VT, EF, and functional status). There was an interaction between variables, such as higher rates of both VT recurrence and mortality, which was observed in patients with lower EF and worse NYHA failure status.^483^, ^484^

### 6.4 Risk of adverse outcomes in patients treated with catheter ablation

**Table:** 

Risk of adverse outcomes in patients treated with catheter ablation	Class	References
The aetiology and severity of cardiomyopathy and inducibility of arrhythmias after VT ablation are useful in determining risk of recurrence of VT after catheter ablation	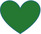	^485^
Risk scores in combination with procedural characteristics may be useful for assessing adverse outcomes associated with catheter ablation of VT	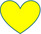	486, 487, 488

Risk of death or acute haemodynamic compromise in patients who undergo catheter ablation of ventricular arrhythmias is driven by patient‐specific factors (comorbidities), procedural factors, and presentation of the patient. In a large retrospective multicentre registry, factors such as low EF, chronic kidney disease, VT storm, and unmappable VTs were associated with early mortality.^489^ As mentioned above, male sex is associated with occurrence of VT/VF and ICD shocks.^490^ As procedural factors are often difficult to determine prior to the procedure, various risk scores have been developed to assess risk of acute haemodynamic compromise and/or death in patients undergoing catheter ablation of VT. Of these, a modified version of the Seattle HF Model and PAINESD score have been used in single centre and multicentre retrospective studies to evaluate risk of acute haemodynamic compromise or death post‐procedure.^486^, ^487^, ^489^ The Seattle HF Model incorporates, amongst other variables, age, EF, blood pressure, weight, gender, HF medications, blood electrolyte, and haemoglobin levels as well as NYHA to predict mortality. A modified version of this model which incorporates VT storm and ICD shocks was recently reported to be potentially more useful in predicting 6 months survival in patients who undergo VT ablation.^488^ The PAINESD score incorporates pulmonary disease, age, presence of ischaemic cardiomyopathy, NYHA, EF, VT storm, and diabetes and assigns a score between 3 and 6 to each of these patient characteristics. In retrospective studies, patients with a PAINESD score greater than 15 had a 24% risk of acute haemodynamic compromise and a significantly higher risk of mortality.^487^, ^489^ Use of these risk scores can be important in discussion of risks and benefits in patients undergoing catheter ablation and may help determine need for haemodynamic support during the procedure. However, larger multicentre prospective studies are required. It is important to note that patients with lower EF and NYHA Class IV HF may still benefit from successful catheter ablation of VT, and freedom from VT after successful ablation is associated with improved mortality.^483^, ^491^

With regard to VT recurrence, in addition to patient related comorbidities, large single centre and multicentre studies have shown that the risk of VT recurrence is driven by the underlying aetiology, particularly in patients with non‐ischaemic heart disease, even after adjusting for other patient comorbidities.^492^, ^493^, ^494^ In particular, patients with Lamin A/C cardiomyopathy, hypertrophic cardiomyopathy, cardiac sarcoidosis, and valvular cardiomyopathy appear to be at higher risk for VT recurrence after catheter ablation as compared to idiopathic dilated cardiomyopathy.^485^, ^492^ In addition, location of scar seems to determine risk of VT recurrence post‐catheter ablation.^495^ In this regard, endocardial ablation alone may be insufficient in many non‐ischaemic cardiomyopathies. In arrhythmogenic right ventricular cardiomyopathy, epicardial presence of scar can serve as the substrate for VT and combined endo‐epicardal mapping and ablation or adjuvant epicardial ablation after endocardial ablation is often required.^496^, ^497^, ^498^, ^499^ Cardiac MRI with LGE can be used in assessment of scar location and may be beneficial in diagnosis and peri‐procedural planning of VT ablation.^500^

Retrospective studies have shown that inducibility of VT at the end of ablation is associated with adverse outcomes, even after adjusting for other patient comorbidities. Non‐inducibility of VT in ischaemic cardiomyopathy patients was shown to be associated with improved arrhythmia‐free survival rates and all‐cause mortality,^501^, ^502^ even after adjusting for other comorbidities. In addition, inducible clinical VT during non‐invasive programmed electrical ventricular stimulation (PES) is associated with decreased 1‐year VT free survival as compared with those who are not inducible (<30% vs. >80%)^503^

Patients who were non‐inducible during non‐invasive PES after ablation had a VT recurrence rate of only 9% at 1 year of follow‐up when both acute (at the end of the procedure) and late (at 6 days post‐procedure) programmed stimulation were negative.^504^ Therefore, PES may be used to guide redo ablation and address ICD programming.

Finally, although catheter ablation is generally performed after the occurrence of ICD therapies, two clinical trials reported the value of catheter ablation prior to or in conjunction with ICD implantation. The Prophylactic Catheter Ablation for Prevention of Defibrillator Therapy clinical trial randomized patients with spontaneous ventricular tachycardia or fibrillation and history of myocardial infarction to ICD or ICD and catheter ablation. In this trial, 30‐day mortality was zero along with a significant reduction in ICD therapies from 31% to 9% between the control (ICD) and intervention arms (ICD + catheter ablation).^505^ The Catheter Ablation of Stable Ventricular Tachycardia before Defibrillator Implantation in Patients with Coronary Heart Disease (VTACH) trial randomized patients with history of myocardial infarction and stable VT to catheter ablation followed by ICD implantation vs. ICD implantation alone and showed that catheter ablation reduced occurrence of VT or VF by 18% at 2 years of follow‐up. These data imply that in patients who receive ICD for secondary prevention and have ischaemic heart disease, catheter ablation can be considered earlier, at the time of ICD implantation, to reduce future ICD therapies and prior to potential presentation with VT storm.^506^ The impact of early ablation (at the time of ICD implantation) on mortality was the subject of the BERLIN‐VT clinical trial, early results of which have indicated a lack of a difference in death or hospitalization for VT/VF in the deferred group (ablation after occurrence of third appropriate shock) vs. those who underwent prophylactic ablation at the time of ICD implantation.^507^ It is important to note that in these studies, patients had a history of VT or VF. In patients with ischaemic heart disease undergoing ICD implantation for primary prevention of sudden cardiac death, prophylactic substrate modification of scar by catheter ablation requires further investigation. In the Substrate Modification Study, patients randomized to ICD implantation plus VT ablation had similar time to VT recurrence as those who underwent ICD implantation only. However, catheter ablation at the time of ICD implantation was associated with a greater than 50% reduction in total number of ICD therapies throughout the follow‐up period.^508^

## 7 HOW TO ASSESS RISK FOR ADVERSE OUTCOME IN PATIENTS WITH OTHER SPECIFIC CARDIAC CONDITIONS

### 7.1 Patients with ventricular premature contractions

**Table:** 

Patients with ventricular premature contractions	Class	References
An evaluation of cardiac function and screening for heart failure symptoms should be considered in patients with frequent ventricular ectopy (>10 000 PVCs within 24 h or >10% over a more extended timeframe)	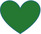	^509^
An evaluation of cardiac function and screening for heart failure symptoms may be considered in patients with frequent multiform PVCs, PVCs with a QRS duration > 150 ms or PVCs with a coupling interval of <450 ms	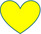	510, 511

Frequent PVCs can lead to cardiomyopathy and HF, and are associated with increased mortality.^509^ In addition, in some patients with an inherited arrhythmogenic cardiomyopathy, PVCs may be the initial clinical manifestation that leads to this diagnosis. An initial case series describing four patients who had reversal of cardiomyopathy after amiodarone successfully suppressed a high PVC burden has resulted in the recognition for the potential reversibility of this condition.^463^ However, only a minority of patients with PVCs will develop symptoms or adverse sequelae. The factors that can potentially predict development of HF and increased risk of adverse outcomes include PVC frequency as well as characteristics of the PVC morphology and timing of the PVC coupling interval.

#### 7.1.1 Premature ventricular complex frequency

In a large cohort of patients, increased PVC frequency was associated with reduced LV function, a higher incidence of heart failure, and a higher risk of death. Specifically, compared to the lowest quartile of PVC frequency (<0.002%), the highest quartile (0.123% to 17.7%) in this cohort of patients with a structurally normal heart at baseline had a 31% increased risk of death over a follow‐up of >13 years.^509^ Other studies correlating frequency with PVC‐induced cardiomyopathy suggested a threshold effect observed at >20%, though there is no accepted cut‐off that appears to be protective.^462^, ^510^ In a study of 239 consecutive patients with apparently normal hearts, a PVC burden of >20 000 in 24 hours was associated with a reduced LVEF, whereas >10 000 but <20 000 showed LV dilation with preserved LVEF.^512^

#### 7.1.2 Premature ventricular complex morphology

In addition to PVC burden, the morphological features of the PVC have been evaluated. The width of the PVC QRS complex, perhaps reflective of dyssynchrony, has been associated with increased risk of developing PVC‐induced cardiomyopathy.^510^, ^511^ In these retrospective studies, patients with a PVC duration of >150 milliseconds appeared to require a lower burden for development of a cardiomyopathy. A PVC duration of >153 milliseconds in patients with a >10% burden, was associated with an 82% sensitivity and 75% specificity for subsequent development of a cardiomyopathy. The presence of multiform PVCs has also been associated with the development of new onset heart failure.^513^

#### 7.1.3 Premature ventricular complex coupling interval

One mechanism of PVC‐induced cardiomyopathy may be due to ineffective mechanical contraction leading to adverse remodelling, possibly related to the timing of the PVC. However, there are only a few small studies evaluating this. In a retrospective cohort study of 510 patients, a PVC coupling interval of <450 milliseconds was associated with a reduced LVEF.^514^ Another smaller study of 70 patients did not show any association, though its power was limited.^515^ Another study specifically identified the presence of interpolated PVCs regardless of coupling interval as associated with reduced LVEF.^516^ A short PVC coupling interval may also be an important determinant of VF, especially in patients with genetic or acquired early or abnormal repolarization.^42^, ^517^, ^518^

While the promise of effective treatment for reversing the potential adverse cardiac effects of frequent PVCs remains a possibility, it remains unclear whether such patients can easily be identified. Most cardiologists accept the dose–response relationship of PVC burden and reduced cardiac function, although the precise threshold for this effect remains unknown. There also is the potential for other factors aside from frequency alone, such as PVC QRS duration and coupling intervals, to influence adverse events associated with frequent PVCs.

### 7.2 Patients with supraventricular tachyarrhythmia such as Wolff–Parkinson–White syndrome and focal atrial tachycardia

**Table:** 

Patients with supraventricular tachyarrhythmia such as WPW syndrome and focal atrial tachycardia	Class	References
EPS, with the use of isoprenaline, is recommended to risk stratify individuals with asymptomatic pre‐excitation who have high‐risk occupations/hobbies, and those who participate in competitive athletics	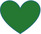	519, 520, 521
EPS should be considered for risk stratification in asymptomatic pre‐excitation patients without high‐risk occupations or those who are not competitive athletes	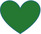	519, 521, 522
Non‐invasive screening with exercise testing, drug testing, and ambulatory monitoring may be considered for risk stratification in asymptomatic pre‐excitation patients without high‐risk occupations or those who are not competitive athletes	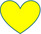	519, 521, 522
High‐risk features to consider at EPS with or without catecholamine challenge are accessory pathways with an antegrade refractory period ≤250 ms, shortest pre‐excited RR interval during AF ≤250 ms, inducible atrioventricular re‐entrant tachycardia, and multiple accessory pathways	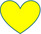	519, 523, 524
Observation without treatment may be reasonable in asymptomatic WPW patients who are considered to be at low risk following EPS, abrupt loss of pre‐excitation during exercise testing, or due to intermittent pre‐excitation on a resting ECG or during ambulatory monitoring	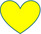	519, 521

Patients with WPW may experience dramatic adverse events including SCD due to VF.^521^ The estimate for the frequency of SCD ranges up to 4% with more recent studies reporting a rate of 2%.^519^ Alarmingly, in approximately half of the patients SCD is the first clinical manifestation of the syndrome rendering appropriate risk stratification essential.^520^

Risk assessment strategies have been recently reviewed in the 2019 ESC Guidelines for the management of patients with supraventricular tachycardia.^525^ Main risk factors for the development of malignant arrhythmias and SCD in patients with pre‐excitation are: (a) a short anterograde refractory period of the accessory pathway with the optimal cut‐off reported to be at 250 milliseconds and (b) inducible atrioventricular reentrant tachycardia triggering pre‐excited AF. A short pre‐excited RR interval during AF ≤250 milliseconds and the presence of multiple accessory pathways have been also reported as risk markers. For these reasons, EPS is recommended for risk stratification in subjects with asymptomatic ventricular pre‐excitation who either have high‐risk occupations or are competitive athletes. In patients without high‐risk occupations or those who are not competitive athletes, EPS should be considered for risk stratification of patients with asymptomatic pre‐excitation that can derive a prognostic benefit from prophylactic catheter ablation of the accessory pathway.^525^ Permanent Junctional Reciprocating Tachycardia (PJRT) re‐presents a rare form of atrioventricular reciprocating tachycardia using a concealed accessory pathway. The incessant behaviour of PJRT may result in tachycardia‐induced cardiomyopathy that usually resolves after successful treatment by RF catheter ablation.

Non‐invasive testing may also be helpful. Non‐invasive findings that identify a pathway not capable of maintaining rapid conduction during AF include intermittent loss of conduction over the accessory pathway on the resting ECG or during ambulatory monitoring, and abrupt loss of pre‐excitation during exercise testing.^523^, ^524^

Focal atrial tachycardias are characterized by regular atrial activation from atrial areas with centrifugal spread and can be classified as sustained or non‐sustained. Sustained focal atria tachycardia in the adult population is usually associated with a benign prognosis, although tachycardia‐mediated cardiomyopathy has been reported in up to 10% of patients referred for ablation of incessant SVT.^526^ Non‐sustained atrial tachycardia is frequently found on Holter recordings and often does not require treatment; however, we should consider that patients with a high premature atrial contractions (PAC) burden (>500/24 h) are at increased risk for developing of AF and be educated on the symptoms of AF.^527^

## SUMMARY

In clinical practice and for scientific purposes, cardiologists and primary care physicians perform risk assessment in patients with cardiac diseases or conditions with high risk of developing such.

The European Heart Rhythm Association (EHRA), Heart Rhythm Society (HRS), Asia Pacific Heart Rhythm Society (APHRS), and the Latin American Heart Rhythm Society (LAHRS) set down this expert consensus statement task force to summarize the consensus regarding risk assessment in cardiac arrhythmias. Objectives were to raise awareness of using the right risk assessment tool for a given outcome in a given population, and to provide physicians with practical proposals that may lead to rational and evidence‐based risk assessment and improvement of patient care in this regard. A large variety of methods are used for risk assessment and choosing the best methods and tools hereof in a given situation is not simple. Even though parameters and test results found associated with increased risk of one outcome (e.g. death) may also be associated with higher risk of other adverse outcomes, specific risk assessment strategies should be used only for the purposes for which they are validated.

The work of this task force is summarized in a row of consensus statement tables.

## CONFLICT OF INTEREST

None declared.

## SUPPORTING INFORMATION

Supplementary material is available at *Europace* online.

## APPENDIX 1

ESC Scientific Document Group: Alfred E. Buxton: Department of Medicine, The Richard A. and Susan F. Smith Center for Outcomes Research in Cardiology, Beth Israel Deaconess Medical Center, Boston, MA, USA; Gonzalo Calvimontes: Asociacion Guatemalteca de Cardiologia, Guatemala; Tze‐Fan Chao: Division of Cardiology, Department of Medicine, Taipei Veterans General Hospital, Taiwan; Lars Eckardt: Department for Cardiology, Electrophysiology, University Hospital Münster, Münster, Germany; Heidi Estner: Department of Medicine, I, University Hospital Munich, Ludwig‐Maximilians University, Munich, Germany; Anne M. Gillis: University of Calgary ‐ Libin Cardiovascular Institute of Alberta, Calgary, AB, Canada; Rodrigo Isa: Clínica RedSalud Vitacura and Hospital el Carmen de Maipú, Santiago, Chile; Josef Kautzner: Institute for Clinical and Experimental Medicine, Prague, Czech Republic; Philippe Maury: Rangueil University Hospital, Toulouse, France; Joshua D. Moss: Department of Cardiac Electrophysiology, University of California San Francisco, San Francisco, USA; Gi‐Byung Nam: Division of Cardiology, Asan Medical Center, University of Ulsan, College of Medicine, Seoul, Republic of Korea; Brian Olshansky: University of lowa Carver College of Medicine, Iowa City, USA; Luis Fernando Pava Molano: Fundación Valle del Lili, Cali, Colombia; Mauricio Pimentel: Cardiology Division, Hospital de Clínicas de Porto Alegre, Porto Alegre, RS, Brazil; Mukund Prabhu: Department of Cardiology, Sree Chitra Tirunal Institute for Medical Sciences and Technology, Trivandrum, India; Wendy S. Tzou: Department of Cardiology/Cardiac Electrophysiology, University of Colorado Anschutz Medical Campus, Aurora, USA; Philipp Sommer: Clinic for Electrophysiology, Herz‐ und Diabeteszentrum, Clinic for Electrophysiology, Ruhr‐Universität Bochum, Bad Oeynhausen, Germany; Janice Swampillai: Waikato Hospital, Hamilton, New Zealand; Alejandro Vidal: Division of Cardiology, McGill University Health Center, Montreal, Canada; Thomas Deneke (Reviewer Coordinator): Clinic for Cardiology II (Interventional Electrophysiology), Heart Center Bad Neustadt, Bad Neustadt a.d. Saale, Germany; Gerhard Hindricks, Department of Electrophysiology, Leipzig Heart Center at University of Leipzig, Leipzig, Germany; Christophe Leclercq (ESC‐CPG representative): Univ Rennes, CHU Rennes, INSERM, Rennes, France.

## References

[1] Benhorin J , Bodenheimer M , Brown M , Case R , Dwyer EM Jr , Eberly S , et al. Improving clinical practice guidelines for practicing cardiologists. Am J Cardiol. 2015;115:1773–6. 10.1016/j.amjcard.2015.03.026 25918027

[2] Lip G . The ABC pathway: an integrated approach to improve AF management. Nat Rev Cardiol. 2017;14:627–8. 10.1038/nrcardio.2017.153 28960189

[3] Proietti M , Romiti GF , Olshansky B , Lane DA , Lip G . Improved outcomes by integrated care of anticoagulated patients with atrial fibrillation using the simple ABC (Atrial Fibrillation Better Care) pathway. Am J Med. 2018;131:1359–66.e6. 10.1016/j.amjmed.2018.06.012 30153428

[4] Pastori D , Pignatelli P , Menichelli D , Violi F , Lip G . Integrated care management of patients with atrial fibrillation and risk of cardiovascular events: the ABC (Atrial fibrillation Better Care) pathway in the ATHERO‐AF study cohort. Mayo Clin Proc. 2019;94:1261–7. 30551910 10.1016/j.mayocp.2018.10.022

[5] Pastori D , Farcomeni A , Pignatelli P , Violi F , Lip GY . ABC (atrial fibrillation better care) pathway and healthcare costs in atrial fibrillation: the ATHERO‐AF study. Am J Med. 2019;132:856–61. 30659810 10.1016/j.amjmed.2019.01.003

[6] Yoon M , Yang PS , Jang E , Yu HT , Kim TH , Uhm JS , et al. Improved population‐based clinical outcomes of patients with atrial fibrillation by compliance with the simple ABC (atrial fibrillation better care) pathway for integrated care management: a nationwide cohort study. Thromb Haemost. 2019;19:1695–703. 10.1055/s-0039-1693516 31266082

[7] Lip GYH , Freedman B , De Caterina R , Potpara TS . Stroke prevention in atrial fibrillation: past, present and future. Comparing the guidelines and practical decision‐making. Thromb Haemost. 2017;117:1230–9. 10.1160/TH16-11-0876 28597905

[8] Borre ED , Goode A , Raitz G , Shah B , Lowenstern A , Chatterjee R , et al. Predicting thromboembolic and bleeding event risk in patients with non‐valvular atrial fibrillation: a systematic review. Thromb Haemost. 2018;118:2171–87. 30376678 10.1055/s-0038-1675400 PMC6754740

[9] Lip GYH , Banerjee A , Boriani G , Chiang CE , Fargo R , Freedman B , et al. Antithrombotic therapy for atrial fibrillation: CHEST guideline and expert panel report. Chest. 2018;154:1121–201. 10.1016/j.chest.2018.07.040 30144419

[10] Rivera‐Caravaca JM , Marín F , Vilchez JA , Gálvez J , Esteve‐Pastor MA , Vicente V , et al. Refining stroke and bleeding prediction in atrial fibrillation by adding consecutive biomarkers to clinical risk scores. Stroke. 2019;50:1372–9. 31084333 10.1161/STROKEAHA.118.024305

[11] Yoon M , Yang PS , Jang E , Yu HT , Kim TH , Uhm JS , et al. Dynamic changes of CHA2DS2VASc score and the risk of ischaemic stroke in Asian patients with atrial fibrillation: a nationwide cohort study. Thromb Haemost. 2018;118:1296–304. 10.1055/s-0038-1651482 29723875

[12] Chao TF , Liao JN , Tuan TC , Lin YJ , Chang SL , Lo LW , et al. Incident co‐morbidities in patients with atrial fibrillation initially with a CHA2DS2VASc score of 0 (males) or 1 (females): implications for reassessment of stroke risk in initially 'low‐risk' patients. Thromb Haemost. 2019;119:1162–70. 10.1055/s-0039-1683933 30900222

[13] Chao TF , Lip GYH , Liu CJ , Lin YJ , Chang SL , Lo LW , et al. Relationship of aging and incident comorbidities to stroke risk in patients with atrial fibrillation. J Am Coll Cardiol. 2018;71:122–32. 10.1016/j.jacc.2017.10.085 29325634

[14] Apostolakis S , Lane DA , Buller H , Lip GY . Comparison of the CHADS2, CHA2DS2VASc and HAS‐BLED scores for the prediction of clinically relevant bleeding in anticoagulated patients with atrial fibrillation: the AMADEUS trial. Thromb Haemost. 2013;110:1074–9. 10.1160/TH13-07-0552 24048467

[15] Chao TF , Lip GYH , Lin YJ , Chang SL , Lo LW , Hu YF , et al. Incident risk factors and major bleeding in patients with atrial fibrillation treated with oral anticoagulants: a comparison of baseline, follow‐up and delta HAS‐BLED scores with an approach focused on modifiable bleeding risk factors. Thromb Haemost. 2018;118:768–77. 10.1055/s-0038-1636534 29510426

[16] Ferrante di Ruffano L , Hyde CJ , McCaffery KJ , Bossuyt PM , Deeks JJ . Assessing the value of diagnostic tests: a framework for designing and evaluating trials. BMJ. 2012;344:e686 10.1136/bmj.e686 22354600

[17] Goldberger JJ , Cain ME , Hohnloser SH , Kadish AH , Knight BP , American LMS , et al. Heart Association/American College of Cardiology Foundation/Heart Rhythm Society scientific statement on noninvasive risk stratification techniques for identifying patients at risk for sudden cardiac death: a scientific statement from the American Heart Association Council on Clinical Cardiology Committee on Electrocardiography and Arrhythmias and Council on Epidemiology and Prevention. Circulation. 2008;118:1497–518. 10.1161/CIRCULATIONAHA.107.189375 18833586

[18] Verrier RL , Klingenheben T , Malik M , El‐Sherif N , Exner DV , Hohnloser SH , et al. Microvolt T‐wave alternans physiological basis, methods of measurement, and clinical utility—consensus guideline by International Society for Holter and Noninvasive Electrocardiology. J Am Coll Cardiol. 2011;58:1309–24. 10.1016/j.jacc.2011.06.029 21920259 PMC4111570

[19] Heart rate variability: standards of measurement, physiological interpretation and clinical use. Task Force of the European Society of Cardiology and the North American Society of Pacing and Electrophysiology. Circulation. 1996;93:1043–65. 8598068

[20] De Bacquer D , Willekens J , De Backer G . Long‐term prognostic value of p‐wave characteristics for the development of atrial fibrillation in subjects aged 55 to 74 years at baseline. Am J Cardiol. 2007;100:850–4. 10.1016/j.amjcard.2007.04.017 17719332

[21] Cheng M , Lu X , Huang J , Zhang S , Gu D . Electrocardiographic PR prolongation and atrial fibrillation risk: a meta‐analysis of prospective cohort studies. J Cardiovasc Electrophysiol. 2015;26:36–41. 10.1111/jce.12539 25199533

[22] Magnani JW , Johnson VM , Sullivan LM , Gorodeski EZ , Schnabel RB , Lubitz SAP , et al. wave duration and risk of longitudinal atrial fibrillation in persons >/= 60 years old (from the Framingham Heart Study). Am J Cardiol. 2011;107:917–21). e1. 10.1016/j.amjcard.2010.10.075 21255761 PMC3049849

[23] Nielsen JB , Kuhl JT , Pietersen A , Graff C , Lind B , Struijk JJ , et al. P‐wave duration and the risk of atrial fibrillation: results from the Copenhagen ECG Study. Heart Rhythm. 2015;12:1887–95. 10.1016/j.hrthm.2015.04.026 25916567

[24] Nikolaidou T , Ghosh JM , Clark AL . Outcomes related to first‐degree atrioventricular block and therapeutic implications in patients with heart failure. JACC Clin Electrophysiol. 2016;2:181–92. 10.1016/j.jacep.2016.02.012 29766868

[25] Buxton AE , Josephson ME . The role of P wave duration as a predictor of postoperative atrial arrhythmias. Chest. 1981;80:68–73. 10.1378/chest.80.1.68 6972856

[26] Kamel H , Soliman EZ , Heckbert SR , Kronmal RA , Longstreth WT Jr , Nazarian S , et al. P‐wave morphology and the risk of incident ischemic stroke in the Multi‐Ethnic Study of Atherosclerosis. Stroke. 2014;45::2786–8. 10.1161/STROKEAHA.114.006364 25052322 PMC4146624

[27] Nikolaidou T , Pellicori P , Zhang J , Kazmi S , Goode KM , Prevalence CJG , et al. and prognostic implications of PR interval prolongation in patients with heart failure. Clin Res Cardiol. 2018;107:108–19. 10.1007/s00392-017-1162-6 28917011 PMC5790844

[28] Aro AL , Anttonen O , Kerola T , Junttila MJ , Tikkanen JT , Rissanen HA , et al. Prognostic significance of prolonged PR interval in the general population. Eur Heart J. 2014;35:123–9. 10.1093/eurheartj/eht176 23677846

[29] Andrade JG , Roy D , Wyse DG , Dorian P , Talajic M , Leduc H , et al. ECG features associated with adverse cardiovascular outcomes in patients with atrial fibrillation: a combined AFFIRM and AF‐CHF analysis. J Cardiovasc Electrophysiol. 2016;27:404–13. 10.1111/jce.12934 27074775

[30] Januszkiewicz Ł , Vegh E , Borgquist R , Bose A , Sharma A , Orencole M , et al. Prognostic implication of baseline PR interval in cardiac resynchronization therapy recipients. Heart Rhythm. 2015;12:2256–62. 10.1016/j.hrthm.2015.06.016 26066291

[31] Rickard J , Karim M , Baranowski B , Cantillon D , Spragg D , Tang WHW , et al. Effect of PR interval prolongation on long‐term outcomes in patients with left bundle branch block vs non‐left bundle branch block morphologies undergoing cardiac resynchronization therapy. Heart Rhythm. 2017;14:1523–8. 10.1016/j.hrthm.2017.05.028 28549996

[32] Das MK , Zipes DP . Fragmented QRS: a predictor of mortality and sudden cardiac death. Heart Rhythm. 2009;6(3 Suppl):S8–14. 10.1016/j.hrthm.2008.10.019 19251229

[33] Rautaharju PM , Surawicz B , Gettes LS , Bailey JJ , Childers R , Deal BJ , et al.;Heart Rhythm Society . AHA/ACCF/HRS recommendations for the standardization and interpretation of the electrocardiogram: Part IV: the ST segment, T and U waves, and the QT interval: a scientific statement from the American Heart Association Electrocardiography and Arrhythmias Committee, Council on Clinical Cardiology; the American College of Cardiology Foundation; and the Heart Rhythm Society: endorsed by the International Society for Computerized Electrocardiology. Circulation. 2009;119:e241–e250. 10.1161/CIRCULATIONAHA.108.191096 19228821

[34] Elming H , Holm E , Jun L , Torp‐Pedersen C , Kober L , Kircshoff M , et al. The prognostic value of the QT interval and QT interval dispersion in all‐cause and cardiac mortality and morbidity in a population of Danish citizens. Eur Heart J. 1998;19:1391–400. 10.1053/euhj.1998.1094 9792266

[35] Dekker JM , Crow RS , Hannan PJ , Schouten EG , Folsom AR , Study A . Heart rate‐corrected QT interval prolongation predicts risk of coronary heart disease in black and white middle‐aged men and women: the ARIC study. J Am Coll Cardiol. 2004;43:565–71. 10.1016/j.jacc.2003.09.040 14975464

[36] Chieng TM , Hau YW , Lim CW . Ventricular tachyarrhythmias prediction methods and its prognostic features: a review. Int J Comput Digital Syst. 2019;8:351–65.

[37] Prenner SB , Shah SJ , Goldberger JJ , Sauer AJ . Repolarization heterogeneity: beyond the QT interval. J Am Heart Assoc. 2016;5(5):1–10. e003607. 10.1161/JAHA.116.003607 PMC4889211 27130347

[38] Porta‐Sanchez A , Spillane DR , Harris L , Xue J , Dorsey P , Care M , et al. T‐wave morphology analysis in congenital long QT syndrome discriminates patients from healthy individuals. JACC Clin Electrophysiol. 2017;3:374–81. 10.1016/j.jacep.2016.10.013 29759450

[39] Hermans BJM , Bennis FC , Vink AS , Koopsen T , Lyon A , Wilde AAM , et al. Improving long‐QT syndrome diagnosis by a polynomial‐based T‐wave morphology characterization. Heart Rhythm. 2020 pii: S1547‐5271(20)30001‐1. 10.1016/j.hrthm.2019.12.020 31917370

[40] Panikkath R , Reinier K , Uy‐Evanado A , Teodorescu C , Hattenhauer J , Mariani R , et al. Prolonged Tpeak‐to‐tend interval on the resting ECG is associated with increased risk of sudden cardiac death. Circ Arrhythm Electrophysiol. 2011;4:441–7. 10.1161/CIRCEP.110.960658 21593198 PMC3157547

[41] Malik M , Huikuri H , Lombardi F , Schmidt G , Zabel M ; On Behalf of e‐Rhythm Study Group of EHRA . Conundrum of the Tpeak‐Tend interval. J Cardiovasc Electrophysiol. 2018;29(5):767–70. 10.1111/jce.13474 29512226

[42] Haissaguerre M , Derval N , Sacher F , Jesel L , Deisenhofer I , de Roy L , et al. Sudden cardiac arrest associated with early repolarization. N Engl J Med. 2008;358:2016–23. 10.1056/NEJMoa071968 18463377

[43] Wu SH , Lin XX , Cheng YJ , Qiang CC , Zhang J . Early repolarization pattern and risk for arrhythmia death: a meta‐analysis. J Am Coll Cardiol. 2013;61:645–50. 10.1016/j.jacc.2012.11.023 23290543

[44] Aagaard P , Baranowski B , Aziz P , Phelan D . Early repolarization in athletes: a review. Circ Arrhythm Electrophysiol. 2016;9:e003577 10.1161/CIRCEP.115.003577 26888446

[45] Bigger JT Jr , Fleiss JL , Kleiger R , Miller JP , Rolnitzky LM . Rolnitzky LM. The relationships among ventricular arrhythmias, left ventricular dysfunction, and mortality in the 2 years after myocardial infarction. Circulation. 1984;69:250–8. 10.1161/01.CIR.69.2.250 6690098

[46] Schmidt G , Malik M , Barthel P , Schneider R , Ulm K , Rolnitzky L , et al. Heart‐rate turbulence after ventricular premature beats as a predictor of mortality after acute myocardial infarction. Lancet. 1999;353(9162):1390–6. 10.1016/S0140-6736(98)08428-1 10227219

[47] Solomon SD , Zelenkofske S , McMurray JJV , Finn PV , Velazquez E , Ertl G , et al. Sudden death in patients with myocardial infarction and left ventricular dysfunction, heart failure, or both. N Engl J Med. 2005;352:2581–8. 10.1056/NEJMoa043938 15972864

[48] Gula LJ , Klein GJ , Hellkamp AS , Massel D , Krahn AD , Skanes AC , et al. Ejection fraction assessment and survival: an analysis of the Sudden Cardiac Death in Heart Failure Trial (SCD‐HeFT). Am Heart J. 2008;156:1196–200. 10.1016/j.ahj.2008.08.007 19033019 PMC2644051

[49] Di Marco A , Anguera I , Schmitt M , Klem I , Neilan TG , White JA , et al. Late gadolinium enhancement and the risk for ventricular arrhythmias or sudden death in dilated cardiomyopathy: systematic review and meta‐analysis. JACC: Heart Fail. 2016;5:28–38. 10.1016/j.jchf.2016.09.017 28017348

[50] Coleman GC , Shaw PW , Balfour PC , Gonzalez JA , Kramer CM , Patel AR , et al. Prognostic value of myocardial scarring on CMR in patients with cardiac sarcoidosis. JACC: Cardiovasc Imaging. 2017;10(4):411–20. 10.1016/j.jcmg.2016.05.009 27450877 PMC5237422

[51] Greulich S , Deluigi CC , Gloekler S , Wahl A , Zürn C , Kramer U , et al. CMR imaging predicts death and other adverse events in suspected cardiac sarcoidosis. JACC: Cardiovasc Imaging. 2013;6(4):501–11. 10.1016/j.jcmg.2012.10.021 23498675

[52] Naruse Y , Sekiguchi Y , Nogami A , Okada H , Yamauchi Y , Machino T , et al. Systematic treatment approach to ventricular tachycardia in cardiac sarcoidosis. Circ Arrhythm Electrophysiol. 2014;7:407–13. 10.1161/CIRCEP.113.000734 24837644

[53] Blankstein R , Osborne M , Naya M , Waller A , Kim CK , Murthy VL , et al. Cardiac positron emission tomography enhances prognostic assessments of patients with suspected cardiac sarcoidosis. J Am Coll Cardiol. 2014;63:329–36. 10.1016/j.jacc.2013.09.022 24140661 PMC3955730

[54] Moss AJ , Hall WJ , Cannom DS , Daubert JP , Higgins SL , Klein H , et al. Improved survival with an implanted defibrillator in patients with coronary disease at high risk for ventricular arrhythmia. N Engl J Med. 1996;335:1933–40. 10.1056/NEJM199612263352601 8960472

[55] Moss AJ , Zareba W , Hall WJ , Klein H , Wilber DJ , Cannom DS , et al. Prophylactic implantation of a defibrillator in patients with myocardial infarction and reduced ejection fraction. N Engl J Med. 2002;346:877–83. 10.1056/NEJMoa013474 11907286

[56] Bardy GH , Lee KL , Mark DB , Poole JE , Packer DL , Boineau R , et al. Amiodarone or an implantable cardioverter‐defibrillator for congestive heart failure. N Engl J Med. 2005;352:225–37. 10.1056/NEJMoa043399 15659722

[57] Moss AJ , Greenberg H , Case RB , Zareba W , Hall WJ , Brown MW , et al. Long‐term clinical course of patients after termination of ventricular tachyarrhythmia by an implanted defibrillator. Circulation. 2004;110:3760–5. 10.1161/01.CIR.0000150390.04704.B7 15583079

[58] Stecker EC , Vickers C , Waltz J , Socoteanu C , John BT , Mariani R , et al. Population‐based analysis of sudden cardiac death with and without left ventricular systolic dysfunction: two‐year findings from the Oregon Sudden Unexpected Death Study. J Am Coll Cardiol. 2006;47:1161–6. 10.1016/j.jacc.2005.11.045 16545646

[59] Gorgels AP , Gijsbers C , de Vreede‐Swagemakers J , Lousberg A . Wellens HJ. Out‐of‐hospital cardiac arrest‐the relevance of heart failure. The Maastricht Circulatory Arrest Registry. Eur Heart J. 2003;24:1204–9. 10.1016/S0195-668X(03)00191-X 12831814

[60] Stevens SM , Reinier K , Chugh SS . Increased left ventricular mass as a predictor of sudden cardiac death: is it time to put it to the test? Circ Arrhythm Electrophysiol. 2013;6:212–7. 10.1161/CIRCEP.112.974931 23424223 PMC3596001

[61] Køber L , Thune JJ , Nielsen JC , Haarbo J , Videbæk L , Korup E , et al. Defibrillator implantation in patients with nonischemic systolic heart failure. N Engl J Med. 2016;375:1221–30. 10.1056/NEJMoa1608029 27571011

[62] Piers SRD , Tao Q , van Huls van Taxis CFB , Schalij MJ , van der Geest RJ , Zeppenfeld K . Contrast‐enhanced MRI‐derived scar patterns and associated ventricular tachycardias in nonischemic cardiomyopathy: implications for the ablation strategy. Circ Arrhythm Electrophysiol. 2013;6:875–3. 10.1161/CIRCEP.113.000537 24036134

[63] White JA , Fine NM , Gula L , Yee R , Skanes A , Klein G , et al. Utility of cardiovascular magnetic resonance in identifying substrate for malignant ventricular arrhythmias. Circ Cardiovasc Imaging. 2012;5:12–20. 10.1161/CIRCIMAGING.111.966085 22038987

[64] Gutman SJ , Costello BT , Papapostolou S , Voskoboinik A , Iles L , Ja J , et al. Reduction in mortality from implantable cardioverter‐defibrillators in non‐ischaemic cardiomyopathy patients is dependent on the presence of left ventricular scar. Eur Heart J. 2019;40:542–50. 10.1093/eurheartj/ehy437 30107489

[65] Fallavollita JA , Heavey BM , Luisi AJ , Michalek SM , Baldwa S , Mashtare TL , et al. Regional myocardial sympathetic denervation predicts the risk of sudden cardiac arrest in ischemic cardiomyopathy. J Am Coll Cardiol. 2014;63:141–9. 10.1016/j.jacc.2013.07.096 24076296 PMC3954563

[66] Buxton AE , Lee KL , Fisher JD , Josephson ME , Prystowsky EN , Hafley G . A randomized study of the prevention of sudden death in patients with coronary artery disease. Multicenter Unsustained Tachycardia Trial Investigators. N Engl J Med. 1999;341:1882–90. 10.1056/NEJM199912163412503 10601507

[67] Brembilla‐Perrot B , Suty‐Selton C , Beurrier D , Houriez P , Nippert M , Terrier de la Chaise A , et al. Differences in mechanisms and outcomes of syncope in patients with coronary disease or idiopathic left ventricular dysfunction as assessed by electrophysiologic testing. J Am Coll Cardiol. 2004;44:594–601. 10.1016/j.jacc.2004.03.075 15358027

[68] Panza JA , Ellis AM , Al‐Khalidi HR , Holly TA , Berman DS , Oh JK , et al. Myocardial viability and long‐term outcomes in ischemic cardiomyopathy. N Engl J Med. 2019;381:739–48. 10.1056/NEJMoa1807365 31433921 PMC6814246

[69] Olshansky B , Hahn EA , Hartz VL , Prater SP , Mason JW . Clinical significance of syncope in the electrophysiologic study versus electrocardiographic monitoring (ESVEM) trial. The ESVEM Investigators. Am Heart J. 1999;137:878–86. 10.1016/S0002-8703(99)70412-6 10220637

[70] Priori SG , Blomstrom‐Lundqvist C , Mazzanti A , Blom N , Borggrefe M , Camm J , et al. ESC Guidelines for the management of patients with ventricular arrhythmias and the prevention of sudden cardiac death: the Task Force for the Management of Patients with Ventricular Arrhythmias and the Prevention of Sudden Cardiac Death of the European Society of Cardiology (ESC). Endorsed by: Association for European Paediatric and Congenital Cardiology (AEPC). Eur Heart J. 2015;36:2793–867. 10.1093/eurheartj/ehv316 26320108

[71] Al‐Khatib SM , Stevenson WG , Ackerman MJ , Gillis AM , Bryant WJ , Hlatky MA , et al. 2017 AHA/ACC/HRS Guideline for management of patients with ventricular arrhythmias and the prevention of sudden cardiac death: executive summary: a report of the American College of Cardiology/American Heart Association Task Force on Clinical Practice Guidelines and the Heart Rhythm Society. Heart Rhythm. 2018;15:e190–e252. 29097320 10.1016/j.hrthm.2017.10.035

[72] Gann D , Tolentino A , Samet P . Electrophysiologic evaluation of elderly patients with sinus bradycardia: a long‐term follow‐up study. Ann Intern Med. 1979;90:24–9. 10.7326/0003-4819-90-1-24 420459

[73] Buxton AE , Fisher JD , Josephson ME , Lee KL , Pryor DB , Prystowsky EN , et al. Prevention of sudden death in patients with coronary artery disease: the Multicenter Unsustained Tachycardia Trial (MUSTT). Prog Cardiovasc Dis. 1993;36:215–26. 10.1016/0033-0620(93)90015-6 8234775

[74] Hilfiker G , Schoenenberger AW , Erne P , Kobza R . Utility of electrophysiological studies to predict arrhythmic events. *World* . J Cardiol. 2015;7:344–50. 10.4330/wjc.v7.i6.344 PMC4478569 26131339

[75] Mankbadi M , Hassan S , McGee M , Jan B , Mangal S , Altier J , et al. Brugada syndrome: the role of risk stratification in selecting patients for implantable cardioverter‐defibrillator placement. Cureus. 2018;10:e2799 10.7759/cureus.2799 30116678 PMC6089703

[76] Brugada J , Campuzano O , Arbelo E , Sarquella‐Brugada G , Brugada R . Present status of Brugada syndrome: JACC state‐of‐the‐art review. J Am Coll Cardiol. 2018;72:1046–59. 10.1016/j.jacc.2018.06.037 30139433

[77] Sroubek J , Probst V , Mazzanti A , Delise P , Hevia JC , Ohkubo K , et al. Programmed ventricular stimulation for risk stratification in the Brugada syndrome: a pooled analysis. Circulation. 2016;133:622–30 10.1161/CIRCULATIONAHA.115.017885 26797467 PMC4758872

[78] Solbiati M , Casazza G , Dipaola F , Barbic F , Caldato M , Montano N , et al. The diagnostic yield of implantable loop recorders in unexplained syncope: a systematic review and meta‐analysis. Int J Cardiol. 2017;231:170–6. 10.1016/j.ijcard.2016.12.128 28052814

[79] Ibrahim OA , Drew D , Hayes CJ , McIntyre W , Seifer CM , Hopman W , et al. Implantable loop recorders in the real world: a study of two Canadian centers. J Interv Card Electrophysiol. 2017;50:179–85. 10.1007/s10840-017-0294-y 29098486

[80] Sanna T , Diener H‐C , Passman RS , Di Lazzaro V , Bernstein RA , Morillo CA , et al. Cryptogenic stroke and underlying atrial fibrillation. N Engl J Med. 2014;370:2478–86. 10.1056/NEJMoa1313600 24963567

[81] Edvardsson N , Frykman V , van Mechelen R , Mitro P , Mohii‐Oskarsson A , Pasquie JL , et al.;PICTURE Study Investigators . Use of an implantable loop recorder to increase the diagnostic yield in unexplained syncope: results from the PICTURE registry. Europace. 2011;13:262–9. 10.1093/europace/euq418 21097478 PMC3024039

[82] Brignole M , Moya A , de Lange FJ , Deharo JC , Elliott PM , Fanciulli A , et al.; ESC Scientific Document Group . 2018 ESC Guidelines for the diagnosis and management of syncope. Eur Heart J. 2018;39:1883–948. 10.5603/KP.2018.0161 29562304

[83] Shen WK , Sheldon RS , Benditt DG , Cohen MI , Forman DE , Goldberger ZD , et al. 2017 ACC/AHA/HRS Guideline for the evaluation and management of patients with syncope: a report of the American College of Cardiology/American Heart Association Task Force on Clinical Practice Guidelines and the Heart Rhythm Society. Circulation. 2017;136:e60–e122. 10.1161/CIR.0000000000000499] 28280231

[84] Roberts PR , Zachariah D , Morgan JM , Yue AM , Greenwood EF , Phillips PC , et al. Monitoring of arrhythmia and sudden death in a hemodialysis population: the CRASH‐ILR Study. PLoS ONE. 2017;12:e0188713 10.1371/journal.pone.0188713 29240772 PMC5730159

[85] Cheung CC , Krahn AD , Andrade JG . The emerging role of wearable technologies in detection of arrhythmia. Can J Cardiol. 2018;34:1083–7. 10.1016/j.cjca.2018.05.003 30049358

[86] Bumgarner JM , Lambert CT , Hussein AA , Cantillon DJ , Baranowski B , Wolski K , et al. Smartwatch algorithm for automated detection of atrial fibrillation. J Am Coll Cardiol. 2018;71:2381–8. 10.1016/j.jacc.2018.03.003 29535065

[87] Liu S . Number of Connected Wearable Devices Worldwide from 2016 to 2022; 2019 [cited 2020 Feb 1]. https://www.statista.com/statistics/487291/global‐connected‐wearable‐devices/

[88] Valiaho E‐S , Kuoppa P , Lipponen JA , Martikainen TJ , Jantti H , Rissanen TT , et al. Wrist band photoplethysmography in detection of individual pulses in atrial fibrillation and algorithm‐based detection of atrial fibrillation. Europace. 2019;21:1031–8. 10.1093/europace/euz060 31505594

[89] Brasier N , Raichle CJ , Dörr M , Becke A , Nohturfft V , Weber S , et al. Detection of atrial fibrillation with a smartphone camera: first prospective, international, two‐centre, clinical validation study (DETECT AF PRO). Europace. 2019;21:41–7. 10.1093/europace/euy176 30085018 PMC6321964

[90] Verbrugge FH , Proesmans T , Vijgen J , Mullens W , Rivero‐Ayerza M , Van Herendael H , et al. Atrial fibrillation screening with photo‐plethysmography through a smartphone camera. Europace. 2019;21:1167–75 10.1093/europace/euz119 31056678

[91] Gillinov S , Etiwy M , Wang R , Blackburn G , Phelan D , Am G , et al. Variable accuracy of wearable heart rate monitors during aerobic exercise. Med Sci Sports Exerc. 2017;49:1697–703. 10.1249/MSS.0000000000001284 28709155

[92] Wang R , Blackburn G , Desai M , Phelan D , Gillinov L , Houghtaling P , et al. Accuracy of wrist‐worn heart rate monitors. JAMA Cardiol. 2017;2:104–6. 10.1001/jamacardio.2016.3340 27732703

[93] Perez MV , Mahaffey KW , Hedlin H , Rumsfeld JS , Garcia A , Ferris T , et al. Large‐scale assessment of a smartwatch to identify atrial fibrillation. N Engl J Med. 2019;381:1909–17. 10.1056/NEJMoa1901183 31722151 PMC8112605

[94] Guo Y , Wang H , Zhang H , Liu T , Liang Z , Xia Y , et al. Mobile photoplethysmographic technology to detect atrial fibrillation. J Am Coll Cardiol. 2019;74:2365–75. 10.1016/j.jacc.2019.08.019 31487545

[95] Ackerman MJ , Priori SG , Willems S , Berul C , Brugada R , Calkins H , et al. HRS/EHRA expert consensus statement on the state of genetic testing for the channelopathies and cardiomyopathies: this document was developed as a partnership between the Heart Rhythm Society (HRS) and the European Heart Rhythm Association (EHRA). Europace. 2011;13:1077–109. 10.1093/europace/eur245 21810866

[96] Haugaa KH , Dan GA , Iliodromitis K , Lenarczyk R , Marinskis G , Osca J , et al. Management of patients with ventricular arrhythmias and prevention of sudden cardiac death‐translating guidelines into practice: results of the European Heart Rhythm Association survey. Europace: european pacing, arrhythmias, and cardiac electrophysiology: journal of the working groups on cardiac pacing, arrhythmias, and cardiac cellular electrophysiology of the. Eur Soc Cardiol. 2018;20:f249–f253. 10.1093/europace/euy112 29878156

[97] Anastasakis A , Papatheodorou E , Ritsatos K , Protonotarios N , Rentoumi V , Gatzoulis K , et al. Sudden unexplained death in the young: epidemiology, aetiology and value of the clinically guided genetic screening. Europace. 2018;20:472–80. 10.1093/europace/euw362 28177452

[98] Pontecorboli G , Figueras I , Carlosena A , Benito E , Prat‐Gonzales S , Padeletti L , et al. Use of delayed‐enhancement magnetic resonance imaging for fibrosis detection in the atria: a review. Europace. 2017;19:180–9. 28172967 10.1093/europace/euw053

[99] Halliday BP , Cleland JGF , Goldberger JJ , Prasad SK . Personalizing risk stratification for sudden death in dilated cardiomyopathy: the past, present, and future. Circulation. 2017;136:215–31. 10.1161/CIRCULATIONAHA.116.027134 28696268 PMC5516909

[100] Margulescu AD , Nunez‐Garcia M , Alarcon F , Benito EM , Enomoto N , Cozzari J , et al. Reproducibility and accuracy of late gadolinium enhancement cardiac magnetic resonance measurements for the detection of left atrial fibrosis in patients undergoing atrial fibrillation ablation procedures. Europace. 2019;21:724–31. 10.1093/europace/euy314 30649273

[101] Goetze JP , Mogelvang R , Maage L , Scharling H , Schnohr P , Sogaard P , et al. Plasma pro‐B‐type natriuretic peptide in the general population: screening for left ventricular hypertrophy and systolic dysfunction. Eur Heart J. 2006;27:3004–10. 10.1093/eurheartj/ehl406 17118955

[102] Darkner S , Goetze JP , Chen X , Henningsen K , Pehrson S , Svendsen JH . Natriuretic propeptides as markers of atrial fibrillation burden and recurrence (from the AMIO‐CAT Trial). Am J Cardiol. 2017;120:1309–15. 10.1016/j.amjcard.2017.07.018 28865890

[103] Sinner MF , Stepas KA , Moser CB , Krijthe BP , Aspelund T , Sotoodehnia N , et al. B‐type natriuretic peptide and C‐reactive protein in the prediction of atrial fibrillation risk: the CHARGE‐AF Consortium of community‐based cohort studies. Europace. 2014;16:1426–33. 10.1093/europace/euu175 25037055 PMC4197895

[104] Kemp Gudmundsdottir K , Fredriksson T , Svennberg E , Al‐Khalili F , Friberg L , Frykman V , et al. Stepwise mass screening for atrial fibrillation using N‐terminal B‐type natriuretic peptide: the STROKESTOP II study. Europace. 2020;22:24–32. 31790147 10.1093/europace/euz255 PMC6945054

[105] Sepehri Shamloo A , Bollmann A , Dagres N , Hindricks G , Arya A . Natriuretic peptides: biomarkers for atrial fibrillation management. Clin Res Cardiol. 2020 10.1007/s00392-020-01608-x. [Epub ahead of print]. 32002634

[106] Engelmann MD , Svendsen JH . Inflammation in the genesis and perpetuation of atrial fibrillation. Eur Heart J. 2005;26:2083–92. 10.1093/eurheartj/ehi350 15975993

[107] Nyrnes A , Njolstad I , Mathiesen EB , Wilsgaard T , Hansen JB , Skjelbakken T , et al. Inflammatory biomarkers as risk factors for future atrial fibrillation. An eleven‐year follow‐up of 6315 men and women: the Tromso study. Gend Med. 2012;9:536–47. e2. 10.1016/j.genm.2012.09.001 23046763

[108] Henningsen KM , Therkelsen SK , Johansen JS , Bruunsgaard H , Svendsen JH . Plasma YKL‐40, a new biomarker for atrial fibrillation? Europace. 2009;11:1032–6. 19411674 10.1093/europace/eup103

[109] Nattel S . Molecular and cellular mechanisms of atrial fibrosis in atrial fibrillation. JACC Clin Electrophysiol. 2017;3:425–35. 10.1016/j.jacep.2017.03.002 29759598

[110] Watanabe T , Takeishi Y , Hirono O , Itoh M , Matsui M , Nakamura K , et al. C‐reactive protein elevation predicts the occurrence of atrial structural remodeling in patients with paroxysmal atrial fibrillation. Heart Vessels. 2005;20:45–9. 10.1007/s00380-004-0800-x 15772777

[111] Henningsen KM , Nilsson B , Bruunsgaard H , Chen X , Pedersen BK , Svendsen JH . Prognostic impact of hs‐CRP and IL‐6 in patients undergoing radiofrequency catheter ablation for atrial fibrillation. Scand Cardiovasc J. 2009;43:285–91. 10.1080/14017430802653676 19117239

[112] Henningsen KM , Therkelsen SK , Bruunsgaard H , Krabbe KS , Pedersen BK , Svendsen JH . Prognostic impact of hs‐CRP and IL‐6 in patients with persistent atrial fibrillation treated with electrical cardioversion. Scand J Clin Lab Invest. 2009;69:425–32. 10.1080/00365510802676848 19204850

[113] Aronson D , Boulos M , Suleiman A , Bidoosi S , Agmon Y , Kapeliovich M , et al. Relation of C‐reactive protein and new‐onset atrial fibrillation in patients with acute myocardial infarction. Am J Cardiol. 2007;100:753–7. 10.1016/j.amjcard.2007.04.014 17719315

[114] Canpolat U , Aytemir K , Hazirolan T , Ozer N , Oto A . Serum YKL‐40 as a marker of left atrial fibrosis assessed by delayed enhancement MRI in lone atrial fibrillation. Pacing Clin Electrophysiol. 2015;38:1386–95. 10.1111/pace.12729 26256257

[115] Henningsen KM , Nilsson B , Johansen JS , Chen X , Pehrson S , Svendsen JH . Plasma YKL‐40 is elevated in patients with recurrent atrial fibrillation after catheter ablation. Inflamm Res. 2010;59:463–9. 10.1007/s00011-009-0146-z 20012147

[116] Marott SCW , Benn M , Johansen JS , Jensen GB , Tybjaerg‐Hansen A , Nordestgaard BG . YKL‐40 levels and atrial fibrillation in the general population. Int J Cardiol. 2013;167(4):1354–9. 10.1016/j.ijcard.2012.04.006 22525348

[117] Van Wagoner DR , Piccini JP , Albert CM , Anderson ME , Benjamin EJ , Brundel B , et al. Progress toward the prevention and treatment of atrial fibrillation: A summary of the Heart Rhythm Society Research Forum on the Treatment and Prevention of Atrial Fibrillation, Washington, DC, December 9–10, 2013. Heart Rhythm. 2015;12(1):e5–e29. 10.1016/j.hrthm.2014.11.011 25460864 PMC4425127

[118] Fenger‐Gron M , Overvad K , Tjonneland A , Frost L . Lean body mass is the predominant anthropometric risk factor for atrial fibrillation. J Am Coll Cardiol. 2017;69:2488–97. 10.1016/j.jacc.2017.03.558 28521886

[119] Sandhu RK , Conen D , Tedrow UB , Fitzgerald KC , Pradhan AD , Ridker PM , et al. Predisposing factors associated with development of persistent compared with paroxysmal atrial fibrillation. J Am Heart Assoc. 2014;3(3):e000916 10.1161/JAHA.114.000916 24786144 PMC4309092

[120] Frost L , Benjamin EJ , Fenger‐Grøn M , Pedersen A , Tjønneland A , Overvad K . Body fat, body fat distribution, lean body mass and atrial fibrillation and flutter. A Danish cohort study. Obesity. 2014;22(6):1546–52. 10.1002/oby.20706 24436019 PMC4169701

[121] Zhuang J , Lu Y , Tang K , Peng W , Xu Y . Influence of body mass index on recurrence and quality of life in atrial fibrillation patients after catheter ablation: a meta‐analysis and systematic review. Clin Cardiol. 2013;36:269–75. 10.1002/clc.22108 23494488 PMC6649556

[122] Abed HS , Samuel CS , Lau DH , Kelly DJ , Royce SG , Alasady M , et al. Obesity results in progressive atrial structural and electrical remodeling: Implications for atrial fibrillation. Heart Rhythm. 2013;10(1):90–100. 10.1016/j.hrthm.2012.08.043 23063864

[123] Priori SG , Wilde AA , Horie M , Cho Y , Behr ER , Berul C , et al. Executive summary: HRS/EHRA/APHRS expert consensus statement on the diagnosis and management of patients with inherited primary arrhythmia syndromes. Europace. 2013;15(10):1389–406. 23994779 10.1093/europace/eut272

[124] Schwartz PJ , Spazzolini C , Crotti L , Bathen J , Amlie JP , Timothy K , et al. The Jervell and Lange‐Nielsen syndrome: natural history, molecular basis, and clinical outcome. Circulation. 2006;113:783–90. 10.1161/CIRCULATIONAHA.105.592899 16461811

[125] Splawski I , Timothy KW , Sharpe LM , Decher N , Kumar P , Bloise R , et al. Ca(V)1.2 calcium channel dysfunction causes a multisystem disorder including arrhythmia and autism. Cell. 2004;119:19–31. 10.1016/j.cell.2004.09.011 15454078

[126] Barsheshet A , Goldenberg I , O‐Uchi J , Moss AJ , Jons C , Shimizu W ,, et al. Mutations in cytoplasmic loops of the KCNQ1 channel and the risk of life‐threatening events: implications for mutation‐specific response to beta‐blocker therapy in type 1 long‐QT syndrome. Circulation. 2012;125:1988–96. 10.1161/CIRCULATIONAHA.111.048041 22456477 PMC3690492

[127] Leenhardt A , Lucet V , Denjoy I , Grau F , Ngoc D , Coumel P . Catecholaminergic polymorphic ventricular tachycardia in children: a 7‐year follow up of 21 patients. Circulation. 1995;91:1512–9. 10.1161/01.CIR.91.5.1512 7867192

[128] Sumitomo N , Harada K , Nagashima M , Yasuda T , Nakamura Y , Aragaki Y , et al. Catecholaminergic polymorphic ventricular tachycardia: electrocardiographic characteristics and optimal therapeutic strategies to prevent sudden death. Heart. 2003;89:66–70. 10.1136/heart.89.1.66 12482795 PMC1767500

[129] van der Werf C , Nederend I , Hofman N , van Geloven N , Ebink C , Frohn‐Mulder IME , et al. Familial evaluation in catecholaminergic polymorphic ventricular tachycardia: disease penetrance and expression in cardiac ryanodine receptor mutation‐carrying relatives. Circ Arrhythm Electrophysiol. 2012;5:748–56. 10.1161/CIRCEP.112.970517 22787013

[130] Kapplinger JD , Tester DJ , Alders M , Benito B , Berthet M , Brugada J , et al. An international compendium of mutations in the SCN5A‐encoded cardiac sodium channel in patients referred for Brugada syndrome genetic testing. Heart Rhythm. 2010;7:33–46. 10.1016/j.hrthm.2009.09.069 20129283 PMC2822446

[131] Liu M , Yang KC , Dudley SC Jr . Cardiac sodium channel mutations: why so many phenotypes? Nat Rev Cardiol. 2014;11:607–15. 10.1038/nrcardio.2014.85 24958080 PMC4878851

[132] Phenotype ELL . genotype, and cellular physiology: need for clarity in characterization. Heart Rhythm. 2012;9:1993–4. 10.1016/j.hrthm.2012.08.033 23085311

[133] Weng L‐C , Lunetta KL , Müller‐Nurasyid M , Smith AV , Thériault S , Weeke PE , et al. Genetic interactions with age, sex, body mass index, and hypertension in relation to atrial fibrillation: the AFGen consortium. Sci Rep. 2017;7:11303 10.1038/s41598-017-09396-7 28900195 PMC5595875

[134] Weng L‐C , Preis SR , Hulme OL , Larson MG , Choi SH , Wang B , et al. Genetic predisposition, clinical risk factor burden, and lifetime risk of atrial fibrillation. Circulation. 2018;137:1027–38. 10.1161/CIRCULATIONAHA.117.031431 29129827 PMC5840011

[135] Ahlberg G , Refsgaard L , Lundegaard PR , Andreasen L , Ranthe MF , Linscheid N , et al. Rare truncating variants in the sarcomeric protein titin associate with familial and early‐onset atrial fibrillation. Nat Commun. 2018;9:4316 10.1038/s41467-018-06618-y 30333491 PMC6193003

[136] Roselli C , Chaffin MD , Weng LC , Aeschbacher S , Ahlberg G , Albert CM , et al. Multi‐ethnic genome‐wide association study for atrial fibrillation. Nat Genet. 2018;50:1225–33. 10.1038/s41588-018-0133-9 29892015 PMC6136836

[137] Hannun AY , Rajpurkar P , Haghpanahi M , Tison GH , Bourn C , Turakhia MP , et al. Cardiologist‐level arrhythmia detection and classification in ambulatory electrocardiograms using a deep neural network. Nat Med. 2019;25:65–9. 10.1038/s41591-018-0268-3 30617320 PMC6784839

[138] Attia ZI , Noseworthy PA , Lopez‐Jimenez F , Asirvatham SJ , Deshmukh AJ , Gersh BJ , et al. An artificial intelligence‐enabled ECG algorithm for the identification of patients with atrial fibrillation during sinus rhythm: a retrospective analysis of outcome prediction. Lancet. 2019;394(10201):861–7. 31378392 10.1016/S0140-6736(19)31721-0

[139] Ebrahimzadeh E , Kalantari M , Joulani M , Shahraki RS , Fayaz F , Ahmadi F . Prediction of paroxysmal Atrial Fibrillation: a machine learning based approach using combined feature vector and mixture of expert classification on HRV signal. Comput Methods Programs Biomed. 2018;165:53–67. 10.1016/j.cmpb.2018.07.014 30337081

[140] Attia ZI , Kapa S , Lopez‐Jimenez F , McKie PM , Ladewig DJ , Satam G , et al. Screening for cardiac contractile dysfunction using an artificial intelligence‐enabled electrocardiogram. Nat Med. 2019;25:70–4. 10.1038/s41591-018-0240-2 30617318

[141] Motwani M , Dey D , Berman DS , Germano G , Achenbach S , Al‐Mallah MH , et al. Machine learning for prediction of all‐cause mortality in patients with suspected coronary artery disease: a 5‐year multicentre prospective registry analysis. Eur Heart J. 2017;38:500–7. 10.1093/eurheartj/ehw188 27252451 PMC5897836

[142] Narula S , Shameer K , Salem Omar AM , Dudley JT , Sengupta PP . Machine‐learning algorithms to automate morphological and functional assessments in 2D echocardiography. J Am Coll Cardiol. 2016;68:2287–95. 10.1016/j.jacc.2016.08.062 27884247

[143] Schnabel RB , Yin X , Gona P , Larson MG , Beiser AS , McManus DD , et al. 50 year trends in atrial fibrillation prevalence, incidence, risk factors, and mortality in the Framingham Heart Study: a cohort study. Lancet 2015;386:154–62. 10.1016/S0140-6736(14)61774-8 25960110 PMC4553037

[144] Andrade JG , Deyell MW , Lee AYK , Macle L . Macle L. Sex differences in atrial fibrillation. Can J Cardiol. 2018;34:429–36. 10.1016/j.cjca.2017.11.022 29455950

[145] Staerk L , Sherer JA , Ko D , Benjamin EJ , Helm RH . Atrial fibrillation: epidemiology, pathophysiology, and clinical outcomes. Circ Res. 2017;120:1501–17. 10.1161/CIRCRESAHA.117.309732 28450367 PMC5500874

[146] Chugh SS , Havmoeller R , Narayanan K , Singh D , Rienstra M , Benjamin EJ , et al. Worldwide epidemiology of atrial fibrillation: a Global Burden of Disease 2010 Study. Circulation. 2014;129:837–47. 10.1161/CIRCULATIONAHA.113.005119 24345399 PMC4151302

[147] Stefansdottir H , Aspelund T , Gudnason V , Arnar DO . Arnar DO. Trends in the incidence and prevalence of atrial fibrillation in Iceland and future projections. Europace. 2011;13:1110–7. 10.1093/europace/eur132 21551478

[148] Miyasaka Y , Barnes ME , Gersh BJ , Cha SS , Bailey KR , Abhayaratna WP , et al. Secular trends in incidence of atrial fibrillation in Olmsted County, Minnesota, 1980 to 2000, and implications on the projections for future prevalence. Circulation. 2006;114:119–25. 10.1161/CIRCULATIONAHA.105.595140 16818816

[149] Kloosterman M , Crijns HJGM , Mulder BA , Groenveld HF , Van Veldhuisen DJ , Rienstra M , et al. Sex‐related differences in risk factors, outcome, and quality of life in patients with permanent atrial fibrillation: results from the RACE II study. Europace. 2019 10.1093/europace/euz300. [Epub ahead of print]. PMC7657385 31747018

[150] Cheng X , Hu Q , Gao L , Liu J , Qin S , Zhang D . Sex‐related differences in catheter ablation of atrial fibrillation: a systematic review and meta‐analysis. Europace. 2019;21:1509–18. 10.1093/europace/euz179 31281922

[151] Andrade J , Khairy P , Dobrev D , Nattel S . The clinical profile and pathophysiology of atrial fibrillation: relationships among clinical features, epidemiology, and mechanisms. Circ Res. 2014;114:1453–68. 10.1161/CIRCRESAHA.114.303211 24763464

[152] Murphy NF , Simpson CR , Jhund PS , Stewart S , Kirkpatrick M , Chalmers J , et al. A national survey of the prevalence, incidence, primary care burden and treatment of atrial fibrillation in Scotland. Heart. 2007;93:606–12. 10.1136/hrt.2006.107573 17277353 PMC1955558

[153] Rodriguez CJ , Soliman EZ , Alonso A , Swett K , Okin PM , Goff DC Jr , et al. Atrial fibrillation incidence and risk factors in relation to race‐ethnicity and the population attributable fraction of atrial fibrillation risk factors: the Multi‐Ethnic Study of Atherosclerosis. Ann Epidemiol. 2015;25(2):71–6. e1. 10.1016/j.annepidem.2014.11.024 25523897 PMC4559265

[154] Naderi S , Wang Y , Miller AL , Rodriguez F , Chung MK , Radford MJ , et al. The impact of age on the epidemiology of atrial fibrillation hospitalizations. Am J Med. 2014;127:158.e1–7. 24332722 10.1016/j.amjmed.2013.10.005 PMC4436031

[155] Heeringa J , van der Kuip DAM , Hofman A , Kors JA , van Herpen G , Stricker BH , et al. Prevalence, incidence and lifetime risk of atrial fibrillation: the Rotterdam study. Eur Heart J. 2006;27:949–53. 10.1093/eurheartj/ehi825 16527828

[156] Chao T‐F , Liu C‐J , Tuan T‐C , Chen T‐J , Hsieh M‐H , Lip GYH , et al. Lifetime risks, projected numbers, and adverse outcomes in Asian patients with atrial fibrillation: a report from the Taiwan nationwide AF cohort study. Chest. 2018;153:453–66. 10.1016/j.chest.2017.10.001 29017957

[157] Ko D , Rahman F , Schnabel RB , Yin X , Benjamin EJ , Christophersen IE . Christophersen IE. Atrial fibrillation in women: epidemiology, pathophysiology, presentation, and prognosis. Nat Rev Cardiol. 2016;13:321–32. 10.1038/nrcardio.2016.45 27053455 PMC5579870

[158] Friberg J , Scharling H , Gadsboll N , Truelsen T , Jensen GB . Copenhagen City Heart Study. Comparison of the impact of atrial fibrillation on the risk of stroke and cardiovascular death in women versus men (The Copenhagen City Heart Study). Am J Cardiol. 2004;94:889–94. 10.1016/j.amjcard.2004.06.023 15464671

[159] Miyasaka Y , Barnes ME , Bailey KR , Cha SS , Gersh BJ , Seward JB , et al. Mortality trends in patients diagnosed with first atrial fibrillation: a 21‐year community‐based study. J Am Coll Cardiol. 2007;49:986–92. 10.1016/j.jacc.2006.10.062 17336723

[160] Kannel WB , Wolf PA , Benjamin EJ , Levy D . Prevalence, incidence, prognosis, and predisposing conditions for atrial fibrillation: population‐based estimates. Am J Cardiol. 1998;82:2n–9n. 10.1016/S0002-9149(98)00583-9 9809895

[161] Boriani G , Glotzer TV , Santini M , West TM , De Melis M , Sepsi M , et al. Device‐detected atrial fibrillation and risk for stroke: an analysis of >10,000 patients from the SOS AF project (Stroke preventiOn Strategies based on Atrial Fibrillation information from implanted devices. Eur Heart J. 2014;35:508–16. 10.1093/eurheartj/eht491 24334432 PMC3930873

[162] Shaikh AY , Maan A , Khan UA , Aurigemma GP , Hill JC , Kane JL , et al. Speckle echocardiographic left atrial strain and stiffness index as predictors of maintenance of sinus rhythm after cardioversion for atrial fibrillation: a prospective study. Cardiovasc Ultrasound. 2012;10:48 10.1186/1476-7120-10-48 23199055 PMC3583741

[163] Marrouche NF , Wilber D , Hindricks G , Jais P , Akoum N , Marchlinski F , et al. Association of atrial tissue fibrosis identified by delayed enhancement MRI and atrial fibrillation catheter ablation: the DECAAF study. JAMA. 2014;311:498–506. 10.1001/jama.2014.3 24496537

[164] Skanes AC , Tang A . Atrial fibrillation and heart failure: untangling a modern Gordian knot. Can J Cardiol. 2018;34:1437–48. 10.1016/j.cjca.2018.07.483 30404749

[165] Smith JG , Newton‐Cheh C , Almgren P , Struck J , Morgenthaler NG , Bergmann A , et al. Assessment of conventional cardiovascular risk factors and multiple biomarkers for the prediction of incident heart failure and atrial fibrillation. J Am Coll Cardiol. 2010;56:1712–9. 10.1016/j.jacc.2010.05.049 21070922 PMC3005324

[166] Ellinor PT , Lunetta KL , Albert CM , Glazer NL , Ritchie MD , Smith AV , et al. Meta‐analysis identifies six new susceptibility loci for atrial fibrillation. Nat Genet. 2012;44:670–5. 10.1038/ng.2261 22544366 PMC3366038

[167] Santhanakrishnan R , Wang N , Larson MG , Magnani JW , McManus DD , Lubitz SA , et al. Atrial fibrillation begets heart failure and vice versa: temporal associations and differences in preserved versus reduced ejection fraction. Circulation. 2016;133:484–92. 10.1161/CIRCULATIONAHA.115.018614 26746177 PMC4738087

[168] Buza V , Rajagopalan B , Curtis AB . Cancer treatment‐induced arrhythmias: focus on chemotherapy and targeted therapies. Circ Arrhythm Electrophysiol. 2017;10 10.1161/CIRCEP.117.005443 28798022

[169] Gami AS , Hodge DO , Herges RM , Olson EJ , Nykodym J , Kara T , et al. Obstructive sleep apnea, obesity, and the risk of incident atrial fibrillation. J Am Coll Cardiol. 2007;49:565–71. 10.1016/j.jacc.2006.08.060 17276180

[170] Djoussé L , Levy D , Benjamin EJ , Blease SJ , Russ A , Larson MG , et al. Long‐term alcohol consumption and the risk of atrial fibrillation in the Framingham Study. Am J Cardiol. 2004;93:710–3. 10.1016/j.amjcard.2003.12.004 15019874

[171] Selmer C , Olesen JB , Hansen ML , Lindhardsen J , Olsen AM , Madsen JC , et al. The spectrum of thyroid disease and risk of new onset atrial fibrillation: a large population cohort study. BMJ. 2012;345:e7895 10.1136/bmj.e7895 23186910 PMC3508199

[172] Robinson K , Frenneaux MP , Stockins B , Karatasakis G , Poloniecki JD , McKenna WJ . Atrial fibrillation in hypertrophic cardiomyopathy: a longitudinal study. J Am Coll Cardiol. 1990;15:1279–85. 10.1016/S0735-1097(10)80014-2 2329232

[173] Go AS , Hylek EM , Phillips KA , Chang YuChiao , Henault LE , Selby JV , et al. Prevalence of diagnosed atrial fibrillation in adults: national implications for rhythm management and stroke prevention: the AnTicoagulation and Risk Factors in Atrial Fibrillation (ATRIA) Study. JAMA. 2001;285:2370–5. 10.1001/jama.285.18.2370 11343485

[174] Magnani JW , Zhu L , Lopez F , Pencina MJ , Agarwal SK , Soliman EZ , et al. P‐wave indices and atrial fibrillation: cross‐cohort assessments from the Framingham Heart Study (FHS) and Atherosclerosis Risk in Communities (ARIC) study. Am Heart J. 2015;169:53–61. e1. 10.1016/j.ahj.2014.10.009 25497248 PMC4269236

[175] Fox CS , Parise H , D'Agostino RB Sr , Lloyd‐Jones DM , Vasan RS , Wang TJ , et al. Parental atrial fibrillation as a risk factor for atrial fibrillation in offspring. JAMA. 2004;291:2851–5. 10.1001/jama.291.23.2851 15199036

[176] Allan V , Honarbakhsh S , Casas J‐P , Wallace J , Hunter R , Schilling R , et al. Are cardiovascular risk factors also associated with the incidence of atrial fibrillation? A systematic review and field synopsis of 23 factors in 32 population‐based cohorts of 20 million participants. Thromb Haemost. 2017;117:837–50. 10.1160/TH16-11-0825 28229164 PMC5442605

[177] Li YG , Pastori D , Farcomeni A , Yang PS , Jang E , Joung B , et al. A simple clinical risk score (C2HEST) for predicting incident atrial fibrillation in Asian subjects: derivation in 471,446 Chinese subjects, with internal validation and external application in 451,199 Korean subjects. Chest. 2019;155(3):510–8. 10.1016/j.chest.2018.09.011 30292759 PMC6437029

[178] Yan‐Guang L , Bisson A , Bodin A , et al. The C2HEST score and the prediction of incident atrial fibrillation in post‐stroke subjects: a French nationwide study. J Am Heart Assoc. 2019;8(13):e012546. 31234697 10.1161/JAHA.119.012546 PMC6662366

[179] Hu WS , Hsieh MH , Lin CL . A novel atrial fibrillation prediction model for Chinese subjects: a nationwide cohort investigation of 682 237 study participants with random forest model. Europace. 2019;21:1307–12. 10.1093/europace/euz036 31067312

[180] Li Y , Pastori D , Guo Y , Wang Y , Lip G . Risk factors for new‐onset atrial fibrillation: a focus on Asian populations. Int J Cardiol. 2018;261:92–8. 10.1016/j.ijcard.2018.02.051 29657061

[181] Hobbs FD , Fitzmaurice DA , Mant J , Murray E , Jowett S , Bryan S , et al. A randomised controlled trial and cost‐effectiveness study of systematic screening (targeted and total population screening) versus routine practice for the detection of atrial fibrillation in people aged 65 and over. The SAFE study. Health Technol Assess. 2005;9:iii–iv, ix–x, 1–74. 10.3310/hta9400 16202350

[182] Long MJ , Jiang CQ , Lam TH , Xu L , Zhang WS , Lin JM , et al. Atrial fibrillation and obesity among older Chinese: the Guangzhou Biobank Cohort Study. Int J Cardiol. 2011;148:48–52. 10.1016/j.ijcard.2009.10.022 19944468

[183] Boriani G , Laroche C , Diemberger I , Fantecchi E , Meeder J , Kurpesa M , et al. Overweight and obesity in patients with atrial fibrillation: sex differences in 1‐year outcomes in the EORP‐AF General Pilot Registry. J Cardiovasc Electrophysiol. 2018;29:566–72. 10.1111/jce.13428 29345382

[184] Proietti M , Guiducci E , Cheli P , Lip GY . Is there an obesity paradox for outcomes in atrial fibrillation? A systematic review and meta‐analysis of non‐vitamin K antagonist oral anticoagulant trials. Stroke. 2017;48:857–66. 10.1161/STROKEAHA.116.015984 28265017

[185] Middeldorp ME , Pathak RK , Meredith M , Mehta AB , Elliott AD , Mahajan R , et al. PREVEntion and regReSsive Effect of weight‐loss and risk factor modification on Atrial Fibrillation: the REVERSE‐AF study. Europace. 2018;20:1929–35. 10.1093/europace/euy117 29912366

[186] Kim D , Yang P‐S , Kim T‐H , Jang E , Shin H , Kim HY , et al. Ideal blood pressure in patients with atrial fibrillation. J Am Coll Cardiol. 2018;72:1233–45. 10.1016/j.jacc.2018.05.076 30190001

[187] Kim T‐H , Yang P‐S , Yu HT , Jang E , Shin H , Kim HY , et al. Effect of hypertension duration and blood pressure level on ischaemic stroke risk in atrial fibrillation: nationwide data covering the entire Korean population. Eur Heart J. 2019;40(10):809–19. 30608537 10.1093/eurheartj/ehy877

[188] Overvad TF , Skjøth F , Lip GYH , Lane DA , Albertsen IE , Rasmussen LH , et al. Duration of diabetes mellitus and risk of thromboembolism and bleeding in atrial fibrillation: nationwide cohort study. Stroke. 2015;46:2168–74. 10.1161/STROKEAHA.115.009371 26152296

[189] Fangel MV , Nielsen PB , Larsen TB , Christensen BO , Overvad TF , Lip GYH , et al. versus type 2 diabetes and thromboembolic risk in patients with atrial fibrillation: a Danish nationwide cohort study. Int J Cardiol. 2018;268:137–42. 10.1016/j.ijcard.2018.05.037 30041778

[190] Lip GY , Clementy N , Pierre B , Boyer M , Fauchier L . The impact of associated diabetic retinopathy on stroke and severe bleeding risk in diabetic patients with atrial fibrillation: the Loire Valley Atrial Fibrillation Project. Chest. 2015;147:1103–10. 10.1378/chest.14-2096 25412290

[191] Ashburner JM , Go AS , Chang Y , Fang MC , Fredman L , Applebaum KM , et al. Effect of diabetes and glycemic control on ischemic stroke risk in AF patients: ATRIA study. J Am Coll Cardiol. 2016;67:239–47. 10.1016/j.jacc.2015.10.080 26796386 PMC4724056

[192] Pisters R , Lane DA , Marin F , Camm AJ , Lip GY . Stroke and thromboembolism in atrial fibrillation. Circ J. 2012;76:2289–304. 10.1253/circj.CJ-12-1036 23001018

[193] Lip GYH , Collet JP , de Caterina R , Fauchier L , Lane DA , Larsen TB , et al. Antithrombotic therapy in atrial fibrillation associated with valvular heart disease: executive summary of a Joint Consensus Document from the European Heart Rhythm Association (EHRA) and European Society of Cardiology Working Group on Thrombosis, Endorsed by the ESC Working Group on Valvular Heart Disease, Cardiac Arrhythmia Society of Southern Africa (CASSA), Heart Rhythm Society (HRS), Asia Pacific Heart Rhythm Society (APHRS), South African Heart (SA Heart) Association and Sociedad Latinoamericana de Estimulacion Cardiaca y Electrofisiologia (SOLEACE). Thromb Haemost. 2017;117:2215–36. 10.1160/TH-17-10-0709 29212110

[194] Banerjee A , Taillandier S , Olesen JB , Lane DA , Lallemand B , Lip GY , et al. Ejection fraction and outcomes in patients with atrial fibrillation and heart failure: the Loire Valley Atrial Fibrillation Project. Eur J Heart Fail. 2012;14:295–301. 10.1093/eurjhf/hfs005 22294759

[195] Melgaard L , Gorst‐Rasmussen A , Lane DA , Rasmussen LH , Larsen TB . Lip GY. Assessment of the CHA2DS2VASc score in predicting ischemic stroke, thromboembolism, and death in patients with heart failure with and without atrial fibrillation. JAMA. 2015;314:1030–8. 10.1001/jama.2015.10725 26318604

[196] Gillinov AM , Bagiella E , Moskowitz AJ , Raiten JM , Groh MA , Bowdish ME , et al. Rate control versus rhythm control for atrial fibrillation after cardiac surgery. N Engl J Med. 2016;374:1911–21. 10.1056/NEJMoa1602002 27043047 PMC4908812

[197] Mathew J . Investigators of the Ischemia Research and Education Foundation; Multicenter Study of Perioperative Ischemia Research Group. A multicenter risk index for atrial fibrillation after cardiac surgery. JAMA. 2004;291:1720–9. 10.1001/jama.291.14.1720 15082699

[198] Aranki SF , Shaw DP , Adams DH , Rizzo RJ , Couper GS , VanderVliet M , et al. Predictors of atrial fibrillation after coronary artery surgery: current trends and impact on hospital resources. Circulation. 1996;94:390–7. 10.1161/01.CIR.94.3.390 8759081

[199] Ahlsson A , Fengsrud E , Bodin L , Englund A . Postoperative atrial fibrillation in patients undergoing aortocoronary bypass surgery carries an eightfold risk of future atrial fibrillation and a doubled cardiovascular mortality. Eur J Cardio‐Thorac Surg. 2010;37:1353–9. 10.1016/j.ejcts.2009.12.033 20138531

[200] Dobrev D , Aguilar M , Heijman J , Guichard J‐B , Nattel S . Postoperative atrial fibrillation: mechanisms, manifestations and management. Nat Rev Cardiol. 2019;1 10.1038/s41569-019-0166-5 30792496

[201] Echahidi N , Pibarot P , O’Hara G , Mechanisms MP . prevention, and treatment of atrial fibrillation after cardiac surgery. J Am Coll Cardiol. 2008;51:793–801. 10.1016/j.jacc.2007.10.043 18294562

[202] Zacharias A , Schwann TA , Riordan CJ , Durham SJ , Shah AS , Habib RH . Obesity and risk of new‐onset atrial fibrillation after cardiac surgery. Circulation. 2005;112:3247–55. 10.1161/CIRCULATIONAHA.105.553743 16286585

[203] Lowres N , Mulcahy G , Jin K , Gallagher R , Neubeck L , Freedman B . Incidence of postoperative atrial fibrillation recurrence in patients discharged in sinus rhythm after cardiac surgery: a systematic review and meta‐analysis. Interact Cardiovasc Thoracic Surg. 2018;26:504–11. 10.1093/icvts/ivx348 29161419

[204] National Collaborating Centre for Chronic Conditions (UK) . Stroke: National Clinical Guideline for Diagnosis and Initial Management of Acute Stroke and Transient Ischaemic Attack (TIA). London: Royal College of Physicians; 2008. 21698846

[205] Jauch EC , Saver JL , Adams HP Jr , Bruno A , Connors JJ , Demaerschalk BM , et al. Guidelines for the early management of patients with acute ischemic stroke: a guideline for healthcare professionals from the American Heart Association/American Stroke Association. Stroke. 2013;44:870–947. 10.1161/STR.0b013e318284056a 23370205

[206] Steinberg JS , Varma N , Cygankiewicz I , Aziz P , Balsam P , Baranchuk A , et al. ISHNE‐HRS expert consensus statement on ambulatory ECG and external cardiac monitoring/telemetry. Heart Rhythm. 2017;14:e55–e96 10.1016/j.hrthm.2017.03.038 28495301

[207] Sposato LA , Cipriano LE , Saposnik G , Vargas ER , Riccio PM , Hachinski V . Hachinski V. Diagnosis of atrial fibrillation after stroke and transient ischaemic attack: a systematic review and meta‐analysis. Lancet Neurol. 2015;14:377–87. 10.1016/S1474-4422(15)70027-X 25748102

[208] Adams HP Jr , Bendixen BH , Kappelle LJ , Biller J , Love BB , Gordon DL , et al. Classification of subtype of acute ischemic stroke. Definitions for use in a multicenter clinical trial. TOAST. Trial of Org 10172 in Acute Stroke Treatment. Stroke. 1993;24:35–41. 10.1161/01.STR.24.1.35 7678184

[209] Dussault C , Toeg H , Nathan M , Wang ZJ , Roux J‐F , Secemsky E . Electrocardiographic monitoring for detecting atrial fibrillation after ischemic stroke or transient ischemic attack: systematic review and meta‐analysis. Circ Arrhythm Electrophysiol. 2015;8:263–9. 10.1161/CIRCEP.114.002521 25639643

[210] Gladstone DJ , Spring M , Dorian P , Panzov V , Thorpe KE , Hall J , et al. Atrial fibrillation in patients with cryptogenic stroke. N Engl J Med. 2014;370:2467–77. 10.1056/NEJMoa1311376 24963566

[211] Li YG , Bisson A , Bodin A , Herbert J , Grammatico‐Guillon L , Joung B , et al. C2 HEST Score and prediction of incident atrial fibrillation in poststroke patients: a French Nationwide Study. J Am Heart Assoc. 2019;8:e012546 10.1161/JAHA.119.012546 31234697 PMC6662366

[212] Abdulla J , Nielsen JR . Is the risk of atrial fibrillation higher in athletes than in the general population? A systematic review and meta‐analysis. Europace. 2009;11:1156–9. 10.1093/europace/eup197 19633305

[213] Heidbuchel H , Anne W , Willems R , Adriaenssens B , Van de Werf F , Ector H . Endurance sports is a risk factor for atrial fibrillation after ablation for atrial flutter. Int J Cardiol. 2006;107:67–72. 10.1016/j.ijcard.2005.02.043 16337500

[214] Aizer A , Gaziano JM , Cook NR , Manson JE , Buring JE , Albert CM . Relation of vigorous exercise to risk of atrial fibrillation. Am J Cardiol. 2009;103:1572–7. 10.1016/j.amjcard.2009.01.374 19463518 PMC2687527

[215] Hoogsteen J , Schep G , Van Hemel NM , Van Der Wall EE . Paroxysmal atrial fibrillation in male endurance athletes. A 9‐year follow up. Europace. 2004;6:222–8. 10.1016/j.eupc.2004.01.004 15121075

[216] Swanson DR . Atrial fibrillation in athletes: implicit literature‐based connections suggest that overtraining and subsequent inflammation may be a contributory mechanism. Med Hypotheses. 2006;66:1085–92. 10.1016/j.mehy.2006.01.006 16504414

[217] Bettoni M , Zimmermann M . Autonomic tone variations before the onset of paroxysmal atrial fibrillation. Circulation. 2002;105:2753–9. 10.1161/01.CIR.0000018443.44005.D8 12057990

[218] McGann C , Akoum N , Patel A , Kholmovski E , Revelo P , Damal K , et al. Atrial fibrillation ablation outcome is predicted by left atrial remodeling on MRI. Circ Arrhythm Electrophysiol. 2014;7:23–30. 10.1161/CIRCEP.113.000689 24363354 PMC4086672

[219] Ho SY , Sanchez‐Quintana D , Cabrera JA , Anderson RH . Anatomy of the left atrium: implications for radiofrequency ablation of atrial fibrillation. J Cardiovasc Electrophysiol. 1999;10:1525–33. 10.1111/j.1540-8167.1999.tb00211.x 10571372

[220] Qureshi WT , Alirhayim Z , Blaha MJ , Juraschek SP , Keteyian SJ , Brawner CA , et al. Cardiorespiratory Fitness and risk of incident atrial fibrillation: results from the Henry Ford Exercise Testing (FIT) project. Circulation. 2015;131:1827–34. 10.1161/CIRCULATIONAHA.114.014833 25904645

[221] Allison MA , Jensky NE , Marshall SJ , Bertoni AG , Cushman M . Sedentary behavior and adiposity‐associated inflammation: the Multi‐Ethnic Study of Atherosclerosis. Am J Preventive Med. 2012;42:8–13. 10.1016/j.amepre.2011.09.023 PMC3244676 22176840

[222] Malmo V , Nes BM , Amundsen BH , Tjonna A‐E , Stoylen A , Rossvoll O , et al. Aerobic interval training reduces the burden of atrial fibrillation in the short term: a randomized trial. Circulation. 2016;133:466–73. 10.1161/CIRCULATIONAHA.115.018220 26733609

[223] Pelliccia A , Culasso F , Di Paolo FM , Maron BJ . Physiologic left ventricular cavity dilatation in elite athletes. Ann Intern Med. 1999;130:23–31. 10.7326/0003-4819-130-1-199901050-00005 9890846

[224] Chung MK , Martin DO , Sprecher D , Wazni O , Kanderian A , Carnes CA , et al. C‐reactive protein elevation in patients with atrial arrhythmias: inflammatory mechanisms and persistence of atrial fibrillation. Circulation. 2001;104:2886–91. 10.1161/hc4901.101760 11739301

[225] Korantzopoulos P , Kolettis T , Siogas K , Goudevenos J . Atrial fibrillation and electrical remodeling: the potential role of inflammation and oxidative stress. Med Sci Monit. 2003;9:Ra225–9. 12960937

[226] Psychari SN , Apostolou TS , Sinos L , Hamodraka E , Liakos G , Kremastinos DT . Kremastinos DT. Relation of elevated C‐reactive protein and interleukin‐6 levels to left atrial size and duration of episodes in patients with atrial fibrillation. Am J Cardiol. 2005;95:764–7. 10.1016/j.amjcard.2004.11.032 15757607

[227] Mont L , Elosua R , Brugada J . Endurance sport practice as a risk factor for atrial fibrillation and atrial flutter. Europace. 2009;11:11–7. 18988654 10.1093/europace/eun289 PMC2638655

[228] Mohlenkamp S , Lehmann N , Breuckmann F , Brocker‐Preuss M , Nassenstein K , Halle M , et al.; on behalf of the Marathon Study Investigators and the Heinz Nixdorf Recall Study Investigators . Running: the risk of coronary events: prevalence and prognostic relevance of coronary atherosclerosis in marathon runners. Eur Heart J. 2008;29:1903–10. 10.1093/eurheartj/ehn163 18426850

[229] Benito B , Gay‐Jordi G , Serrano‐Mollar A , Guasch E , Shi Y , Tardif J‐C , et al. Cardiac arrhythmogenic remodeling in a rat model of long‐term intensive exercise training. Circulation. 2011;123:13–22. 10.1161/CIRCULATIONAHA.110.938282 21173356

[230] Boraita A , Santos‐Lozano A , Heras ME , Gonzalez‐Amigo F , Lopez‐Ortiz S , Villacastin JP , et al. Incidence of atrial fibrillation in elite athletes. JAMA Cardiol. 2018;3:1200 10.1001/jamacardio.2018.3482 30383155 PMC6583086

[231] Cuspidi C , Tadic M , Sala C , Gherbesi E , Grassi G , Mancia G . Left atrial function in elite athletes: a meta‐analysis of two‐dimensional speckle tracking echocardiographic studies. Clin Cardiol. 2019;42:579–87. 10.1002/clc.23180 30907013 PMC6523010

[232] Pizzale S , Gollob MH , Gow R , Birnie DH . Sudden death in a young man with catecholaminergic polymorphic ventricular tachycardia and paroxysmal atrial fibrillation. J Cardiovasc Electrophysiol. 2008;19:1319–21. 10.1111/j.1540-8167.2008.01211.x 18554199

[233] Miyake CY , Webster G , Czosek RJ , Kantoch MJ , Dubin AM , Avasarala K , et al. Efficacy of implantable cardioverter defibrillators in young patients with catecholaminergic polymorphic ventricular tachycardia: success depends on substrate. Circ Arrhythm Electrophysiol. 2013;6:579–87. 10.1161/CIRCEP.113.000170 23667268

[234] Antzelevitch C , Brugada P , Borggrefe M , Brugada J , Brugada R , Corrado D , et al. Brugada syndrome: report of the second consensus conference. Heart Rhythm. 2005;2:429–40. 10.1016/j.hrthm.2005.01.005 15898165

[235] Brugada P , Brugada J . Right bundle branch block, persistent ST segment elevation and sudden cardiac death: a distinct clinical and electrocardiographic syndrome. A multicenter report. J Am Coll Cardiol. 1992;20:1391–6. 10.1016/0735-1097(92)90253-J 1309182

[236] Eckardt L , Kirchhof P , Loh P , Schulze‐bahr E , Johna R , Wichter T , et al. Brugada syndrome and supraventricular tachyarrhythmias: a novel association? J Cardiovasc Electrophysiol. 2001;12:680–5. 10.1046/j.1540-8167.2001.00680.x 11405402

[237] Morita H , Kusano‐Fukushima K , Nagase S , Fujimoto Y , Hisamatsu K , Fujio H , et al. Atrial fibrillation and atrial vulnerability in patients with Brugada syndrome. J Am Coll Cardiol. 2002;40(8):1437–44. 10.1016/S0735-1097(02)02167-8 12392834

[238] Francis J , Antzelevitch C . Atrial fibrillation and Brugada syndrome. J Am Coll Cardiol. 2008;51:1149–53. 10.1016/j.jacc.2007.10.062 18355651 PMC2367004

[239] Bordachar P , Reuter S , Garrigue S , Cai X , Hocini M , Jais P , et al. Incidence, clinical implications and prognosis of atrial arrhythmias in Brugada syndrome. Eur Heart J. 2004;25:879–84. 10.1016/j.ehj.2004.01.004 15140537

[240] Bigi MA , Aslani A , Shahrzad S . Clinical predictors of atrial fibrillation in Brugada syndrome. Europace. 2007;9:947–50. 17540664 10.1093/europace/eum110

[241] Sacher Frédéric , Probst V , Iesaka Y , Jacon P , Laborderie J , Mizon‐Gérard Frédérique , et al. Outcome after implantation of a cardioverter‐defibrillator in patients with Brugada syndrome: a multicenter study. Circulation. 2006;114:2317–24. 10.1161/CIRCULATIONAHA.106.628537 17116772

[242] Johnson JN , Tester DJ , Perry J , Salisbury BA , Reed CR , Ackerman MJ . Prevalence of early‐onset atrial fibrillation in congenital long QT syndrome. Heart Rhythm. 2008;5:704–9. 10.1016/j.hrthm.2008.02.007 18452873 PMC3940082

[243] Knoche JW , Orland KM , January CT , Maginot KR . Atrial fibrillation and long QT syndrome presenting in a 12‐year‐old girl. Case Rep Pediatr. 2012;2012:124838 10.1155/2012/124838 23193492 PMC3501806

[244] Sankaranarayanan R , Kirkwood G , Dibb K , Garratt CJ . Comparison of atrial fibrillation in the young versus that in the elderly: a review. Cardiol Res Pract. 2013;2013:1–16. 10.1155/2013/976976 PMC3564268 23401843

[245] Chen YH , Xu SJ , Bendahhou S , Wang XL , Wang Y , Xu WY , et al. KCNQ1 gain‐of‐function mutation in familial atrial fibrillation. Science. 2003;299:251–4. 10.1126/science.1077771 12522251

[246] Lieve KV , Verkerk AO , Podliesna S , van der Werf C , Tanck MW , Hofman N , et al. Gain‐of‐function mutation in SCN5A causes ventricular arrhythmias and early onset atrial fibrillation. Int J Cardiol. 2017;236:187–93. 10.1016/j.ijcard.2017.01.113 28262340

[247] Kirchhof P , Eckardt L , Franz MR , Monnig G , Loh P , Wedekind H , et al. Prolonged atrial action potential durations and polymorphic atrial tachyarrhythmias in patients with long QT syndrome. J Cardiovasc Electrophysiol. 2003;14:1027–33. 10.1046/j.1540-8167.2003.03165.x 14521653

[248] Maruyama M , Joung B , Tang L , Shinohara T , On Y‐K , Han S , et al. Diastolic intracellular calcium‐membrane voltage coupling gain and postshock arrhythmias: role of Purkinje fibers and triggered activity. Circ Res. 2010;106:399–408. 10.1161/CIRCRESAHA.109.211292 19926871 PMC2818796

[249] Morotti S , McCulloch AD , Bers DM , Edwards AG , Grandi E . Atrial‐selective targeting of arrhythmogenic phase‐3 early afterdepolarizations in human myocytes. J Mol Cell Cardiol. 2016;96:63–71. 10.1016/j.yjmcc.2015.07.030 26241847 PMC4734906

[250] Brugada R , Hong K , Dumaine R , Cordeiro J , Gaita F , Borggrefe M , et al. Sudden death associated with short‐QT syndrome linked to mutations in HERG. Circulation. 2004;109:30–5. 10.1161/01.CIR.0000109482.92774.3A 14676148

[251] Hong K , Bjerregaard P , Gussak I , Brugada R . Short QT syndrome and atrial fibrillation caused by mutation in KCNH2. J Cardiovasc Electrophysiol. 2005;16:394–6. 10.1046/j.1540-8167.2005.40621.x 15828882

[252] Priori SG , Pandit SV , Rivolta I , Berenfeld O , Ronchetti E , Dhamoon A , et al. A novel form of short QT syndrome (SQT3) is caused by a mutation in the KCNJ2 gene. Circ Res. 2005;96:800–7. 10.1161/01.RES.0000162101.76263.8c 15761194

[253] Priori SG , Napolitano C , Tiso N , Memmi M , Vignati G , Bloise T , et al. Mutations in the cardiac ryanodine receptor gene (hRyR2) underlie catecholaminergic polymorphic ventricular tachycardia. Circulation. 2001;103:196–200. 10.1161/01.CIR.103.2.196 11208676

[254] Postma AV , Denjoy I , Hoorntje TM , Lupoglazoff J‐M , Da Costa A , Sebillon P , et al. Absence of calsequestrin 2 causes severe forms of catecholaminergic polymorphic ventricular tachycardia. Circ Res. 2002;91:1–6. 12386154 10.1161/01.res.0000038886.18992.6b

[255] Marsman RF , Barc J , Beekman L , Alders M , Dooijes D , van den Wijngaard A , et al. A mutation in CALM1 encoding calmodulin in familial idiopathic ventricular fibrillation in childhood and adolescence. J Am Coll Cardiol. 2014;63:259–66. 10.1016/j.jacc.2013.07.091 24076290

[256] Lieve KVV , Verhagen JMA , Wei J , Bos JM , van der Werf C , Roses i Noguer F , et al. Linking the heart and the brain: neurodevelopmental disorders in patients with catecholaminergic polymorphic ventricular tachycardia. Heart Rhythm. 2019;16:220–8. 10.1016/j.hrthm.2018.08.025 30170228

[257] Lip GYH , Jensen M , Melgaard L , Skjoth F , Nielsen PB , Larsen TB . Stroke and bleeding risk scores in patients with atrial fibrillation and valvular heart disease: evaluating 'valvular heart disease' in a nationwide cohort study. Europace. 2019;21:33–40. 10.1093/europace/euy151 29986001

[258] Dagres N , Chao TF , Fenelon G , Aguinaga L , Benhayon D , Benjamin EJ , et al.;ESC Scientific Document Group . European Heart Rhythm Association (EHRA)/Heart Rhythm Society (HRS)/Asia Pacific Heart Rhythm Society (APHRS)/Latin American Heart Rhythm Society (LAHRS) expert consensus on arrhythmias and cognitive function: what is the best practice? Europace. 2018;20:1399–421. 10.1093/europace/euy046 29562326 PMC6658813

[259] Krawczyk M , Fridman S , Cheng Y , Fang J , Saposnik G , Sposato LA . Atrial fibrillation diagnosed after stroke and dementia risk: cohort study of first‐ever ischaemic stroke patients aged 65 or older. Europace. 2019 10.1093/europace/euz237. [Epub ahead of print]. 31531673

[260] Andrieu S , Coley N , Lovestone S , Aisen PS , Vellas B . Prevention of sporadic Alzheimer's disease: lessons learned from clinical trials and future directions. Lancet Neurol. 2015;14:926–44. 10.1016/S1474-4422(15)00153-2 26213339

[261] Kirchhof P , Benussi S , Kotecha D , Ahlsson A , Atar D , Casadei B , et al. ESC Guidelines for the management of atrial fibrillation developed in collaboration with EACTS. Europace. 2016;18:1609–78. 10.1093/europace/euw295 27567465

[262] Hippisley‐Cox J , Coupland C , Brindle P . Derivation and validation of QStroke score for predicting risk of ischaemic stroke in primary care and comparison with other risk scores: a prospective open cohort study. BMJ. 2013;346(may02 1):f2573 10.1136/bmj.f2573 23641033 PMC3641809

[263] Singer DE , Chang Y , Borowsky LH , Fang MC , Pomernacki NK , Udaltsova N , et al. A new risk scheme to predict ischemic stroke and other thromboembolism in atrial fibrillation: the ATRIA study stroke risk score. J Am Heart Assoc. 2013;2:e000250 10.1161/JAHA.113.000250 23782923 PMC3698792

[264] Hijazi Z , Lindback J , Alexander JH , Hanna M , Held C , Hylek EM , et al. The ABC (age, biomarkers, clinical history) stroke risk score: a biomarker‐based risk score for predicting stroke in atrial fibrillation. Eur Heart J. 2016;37:1582–90. 10.1093/eurheartj/ehw054 26920728 PMC4875560

[265] Hart RG , Pearce LA , Aguilar MI . Meta‐analysis: antithrombotic therapy to prevent stroke in patients who have nonvalvular atrial fibrillation. Ann Intern Med. 2007;146:857–67. 10.7326/0003-4819-146-12-200706190-00007 17577005

[266] Fauchier L , Clementy N , Bisson A , Ivanes F , Angoulvant D , Babuty D , et al. Should atrial fibrillation patients with only 1 nongender‐related CHA_2_DS_2_‐VASc risk factor be anticoagulated? Stroke. 2016;47:1831–6. 10.1161/STROKEAHA.116.013253 27231269

[267] Lip GY , Clementy N , Pericart L , Banerjee A , Fauchier L . Stroke and major bleeding risk in elderly patients aged >/=75 years with atrial fibrillation: the Loire Valley atrial fibrillation project. Stroke. 2015;46:143–50. 10.1161/STROKEAHA.114.007199 25424474

[268] Chao T‐F , Lip GYH , Lin Y‐J , Chang S‐L , Lo L‐W , Hu Y‐F , et al. Age threshold for the use of non‐vitamin K antagonist oral anticoagulants for stroke prevention in patients with atrial fibrillation: insights into the optimal assessment of age and incident comorbidities. Eur Heart J. 2019;40:1504–14. 10.1093/eurheartj/ehy837 30605505

[269] Sepehri Shamloo A , Dagres N , Mussigbrodt A , Stauber A , Kircher S , Richter S , et al. Atrial fibrillation and cognitive impairment: new insights and future directions. Heart Lung Circ. 2020;29:69–85. 10.1016/j.hlc.2019.05.185 31262618

[270] Schwarz N , Kuniss M , Nedelmann M , Kaps M , Bachmann G , Neumann T , et al. Neuropsychological decline after catheter ablation of atrial fibrillation. Heart Rhythm. 2010;7:1761–7. 10.1016/j.hrthm.2010.07.035 20691284

[271] Saw J , Tzikas A , Shakir S , Gafoor S , Omran H , Nielsen‐Kudsk JE , et al. Incidence and clinical impact of device‐associated thrombus and peri‐device leak following left atrial appendage closure with the Amplatzer Cardiac Plug. JACC Cardiovasc Interv. 2017;10:391–9. 10.1016/j.jcin.2016.11.029 28231907

[272] Viles‐Gonzalez JF , Kar S , Douglas P , Dukkipati S , Feldman T , Horton R , et al. The clinical impact of incomplete left atrial appendage closure with the Watchman Device in patients with atrial fibrillation: a PROTECT AF (Percutaneous Closure of the Left Atrial Appendage Versus Warfarin Therapy for Prevention of Stroke in Patients With Atrial Fibrillation) substudy. J Am Coll Cardiol. 2012;59:923–9. 10.1016/j.jacc.2011.11.028 22381428

[273] Dukkipati SR , Kar S , Holmes DR , Doshi SK , Swarup V , Gibson DN , et al. Device‐related thrombus after left atrial appendage closure. Circulation. 2018;138:874–85. 10.1161/CIRCULATIONAHA.118.035090 29752398

[274] Fauchier L , Cinaud A , Brigadeau F , Lepillier A , Pierre B , Abbey S , et al. Device‐related thrombosis after percutaneous left atrial appendage occlusion for atrial fibrillation. J Am Coll Cardiol. 2018;71:1528–36. 10.1016/j.jacc.2018.01.076 29622159

[275] Saw J , Fahmy P , DeJong P , Lempereur M , Spencer R , Tsang M , et al. Cardiac CT angiography for device surveillance after endovascular left atrial appendage closure. Eur Heart J Cardiovasc Imaging. 2015;16:1198–206. 10.1093/ehjci/jev067 25851318 PMC4609159

[276] Berte B , Jost CA , Maurer D , Fäh‐Gunz A , Pillois X , Naegeli B , et al. Long‐term follow‐up after left atrial appendage occlusion with comparison of transesophageal echocardiography vs. computed tomography to guide medical therapy and data about post‐closure cardioversion. J Cardiovasc Electrophysiol. 2017;28:1140–50. 10.1111/jce.13289 28675629

[277] Wolf RK , Schneeberger EW , Osterday R , Miller D , Merrill W , Flege JB Jr , et al. Video‐assisted bilateral pulmonary vein isolation and left atrial appendage exclusion for atrial fibrillation. J Thorac Cardiovasc Surg. 2005;130:797–802. 10.1016/j.jtcvs.2005.03.041 16153931

[278] Calkins H , Hindricks G , Cappato R , Kim YH , Saad EB , Aguinaga L , et al. HRS/EHRA/ECAS/APHRS/SOLAECE expert consensus statement on catheter and surgical ablation of atrial fibrillation: executive summary. Europace. 2017;2018(20):157–208. 10.1093/europace/eux275 PMC5892164 29016841

[279] Kirchhof P , Benussi S , Kotecha D , Ahlsson A , Atar D , Casadei B , et al. 2016 ESC Guidelines for the management of atrial fibrillation developed in collaboration with EACTS. Eur Heart J. 2016;37:2893–962. 10.1093/eurheartj/ehw210 27567408

[280] Schellinger PD , Tsivgoulis G , Steiner T , Kohrmann M . Percutaneous left atrial appendage occlusion for the prevention of stroke in patients with atrial fibrillation: review and critical appraisal. J Stroke. 2018;20:281–91. 10.5853/jos.2018.02537 30309224 PMC6186917

[281] Sharma SP , Park P , Lakkireddy D . Left atrial appendages occlusion: current status and prospective. Korean Circ J. 2018;48:692–704. 10.4070/kcj.2018.0231 30073807 PMC6072669

[282] Caliskan E , Sahin A , Yilmaz M , Seifert B , Hinzpeter R , Alkadhi H , et al. Epicardial left atrial appendage AtriClip occlusion reduces the incidence of stroke in patients with atrial fibrillation undergoing cardiac surgery. Europace. 2018;20:e105–e114. 10.1093/europace/eux211 29016813

[283] Reddy VY , Sievert H , Halperin J , Doshi SK , Buchbinder M , Neuzil P , et al. Percutaneous left atrial appendage closure vs warfarin for atrial fibrillation: a randomized clinical trial. JAMA. 2014;312:1988–98. 10.1001/jama.2014.15192 25399274

[284] Holmes DR Jr , Kar S , Price MJ , Whisenant B , Sievert H , Doshi SK , et al. Prospective randomized evaluation of the Watchman Left Atrial Appendage Closure device in patients with atrial fibrillation versus long‐term warfarin therapy: the PREVAIL trial. J Am Coll Cardiol. 2014;64:1–12. 10.1016/j.jacc.2014.04.029 24998121

[285] Reddy VY , Doshi SK , Kar S , Gibson DN , Price MJ , Huber K , et al. 5‐Year outcomes after left atrial appendage closure: from the PREVAIL and PROTECT AF trials. J Am Coll Cardiol. 2017;70:2964–75. 10.1016/j.jacc.2017.10.021 29103847

[286] Aryana A , Singh SK , Singh SM , Gearoid O’Neill P , Bowers MR , Allen SL , et al. Association between incomplete surgical ligation of left atrial appendage and stroke and systemic embolization. Heart Rhythm 2015;12:1431–7. 10.1016/j.hrthm.2015.03.028 25998141

[287] Mohanty S , Gianni C , Trivedi C , Gadiyaram V , Della Rocca DG , MacDonald B , et al. Risk of thromboembolic events after percutaneous left atrial appendage ligation in patients with atrial fibrillation: long‐term results from a multicenter study. Heart Rhythm. 2019 10.1016/j.hrthm.2019.08.003 31400519

[288] Kotecha D , Piccini JP . Atrial fibrillation in heart failure: what should we do? Eur Heart J. 2015;36:3250–7. 10.1093/eurheartj/ehv513 26419625 PMC4670966

[289] Wang TJ , Larson MG , Levy D , Vasan RS , Leip EP , Wolf PA , et al. Temporal relations of atrial fibrillation and congestive heart failure and their joint influence on mortality: the Framingham Heart Study. Circulation. 2003;107:2920–5. 10.1161/01.CIR.0000072767.89944.6E 12771006

[290] Denham NC , Pearman CM , Caldwell JL , Madders GW , Eisner DA , Trafford AW , et al. Calcium in the pathophysiology of atrial fibrillation and heart failure. Front Physiol. 2018;9 10.3389/fphys.2018.01380 PMC6180171 30337881

[291] Deedwania PC , Lardizabal JA . Atrial fibrillation in heart failure: a comprehensive review. Am J Med. 2010;123:198–204. 10.1016/j.amjmed.2009.06.033 20193823

[292] Muller‐Edenborn B , Minners J , Allgeier J , Burkhardt T , Lehrmann H , Ruile P , et al. Rapid improvement in left ventricular function after sinus rhythm restoration in patients with idiopathic cardiomyopathy and atrial fibrillation. Europace. 2019;21:871–8. 10.1093/europace/euz013 31157388

[293] Stewart S , Hart CL , Hole DJ , McMurray JJ . A population‐based study of the long‐term risks associated with atrial fibrillation: 20‐year follow‐up of the Renfrew/Paisley study. Am J Med. 2002;113:359–64. 10.1016/S0002-9343(02)01236-6 12401529

[294] Olsson LG , Swedberg K , Ducharme A , Granger CB , Michelson EL , McMurray JJ , et al. Atrial fibrillation and risk of clinical events in chronic heart failure with and without left ventricular systolic dysfunction: results from the Candesartan in Heart failure‐Assessment of Reduction in Mortality and morbidity (CHARM) program. J Am Coll Cardiol. 2006;47:1997–2004. 10.1016/j.jacc.2006.01.060 16697316

[295] Kotecha D , Chudasama R , Lane DA , Kirchhof P , Lip GY . Atrial fibrillation and heart failure due to reduced versus preserved ejection fraction: a systematic review and meta‐analysis of death and adverse outcomes. Int J Cardiol. 2016;203:660–6. 10.1016/j.ijcard.2015.10.220 26580351

[296] Packer DL , Mark DB , Robb RA , Monahan KH , Bahnson TD , Poole JE , et al.;for the CABANA Investigators . Effect of catheter ablation vs antiarrhythmic drug therapy on mortality, stroke, bleeding, and cardiac arrest among patients with atrial fibrillation: the CABANA randomized clinical trial. JAMA. 2019;321:1261 10.1001/jama.2019.0693 30874766 PMC6450284

[297] Marrouche NF , Brachmann J , Andresen D , Siebels J , Boersma L , Jordaens L , et al. Catheter ablation for atrial fibrillation with heart failure. N Engl J Med. 2018;378:417–27. 10.1056/NEJMoa1707855 29385358

[298] Mathew JS , Marzec LN , Kennedy KF , Jones PG , Varosy PD , Masoudi FA , et al. Atrial fibrillation in heart failure US ambulatory cardiology practices and the potential for uptake of catheter ablation: an National Cardiovascular Data Registry (NCDR((R))) Research to Practice (R2P) Project. J Am Heart Assoc. 2017;6(8):1–11. e005273. 10.1161/JAHA.116.005273 PMC5586408 28862932

[299] Healey JS , Oldgren J , Ezekowitz M , Zhu J , Pais P , Wang J , et al. Occurrence of death and stroke in patients in 47 countries 1 year after presenting with atrial fibrillation: a cohort study. Lancet. 2016;388:1161–9. 10.1016/S0140-6736(16)30968-0 27515684

[300] Fauchier L , Samson A , Chaize G , Gaudin AF , Vainchtock A , Bailly C , et al. Cause of death in patients with atrial fibrillation admitted to French hospitals in 2012: a nationwide database study. *Open* . Heart. 2015;2:e000290 10.1136/openhrt-2015-000290 PMC4680587 26688739

[301] Pokorney SD , Piccini JP , Stevens SR , Patel MR , Pieper KS , Halperin JL , et al. Cause of death and predictors of all‐cause mortality in anticoagulated patients with nonvalvular atrial fibrillation: data from ROCKET AF. J Am Heart Assoc. 2016;7:3 e00219. 10.1161/JAHA.118.008755 PMC4943233 26955859

[302] Turagam MK , Velagapudi P , Visotcky A , Szabo A , Kocheril AG . African Americans have the highest risk of in‐hospital mortality with atrial fibrillation related hospitalizations among all racial/ethnic groups: a nationwide analysis. Int J Cardiol. 2012;158:165–6. 10.1016/j.ijcard.2012.04.090 22564385

[303] Jacobs V , May HT , Bair TL , Crandall BG , Cutler M , Day JD , et al. The impact of risk score (CHADS2 versus CHA2DS2VASc) on long‐term outcomes after atrial fibrillation ablation. Heart Rhythm. 2015;12:681–6. 10.1016/j.hrthm.2014.12.034 25546809

[304] Fox KAA , Lucas JE , Pieper KS , Bassand J‐P , Camm AJ , Fitzmaurice DA , et al. Improved risk stratification of patients with atrial fibrillation: an integrated GARFIELD‐AF tool for the prediction of mortality, stroke and bleed in patients with and without anticoagulation. BMJ Open. 2017;7:e017157 10.1136/bmjopen-2017-017157 PMC5778339 29273652

[305] Niederdockl J , Simon A , Schnaubelt S , Schuetz N , Laggner R , Sulzgruber P , et al. Cardiac biomarkers predict mortality in emergency patients presenting with atrial fibrillation. Heart. 2019;105:482–8. 10.1136/heartjnl-2018-313145 30415208 PMC6580776

[306] Hijazi Z , Oldgren J , Lindback J , Alexander JH , Connolly SJ , Eikelboom JW , et al.;the ARISTOTLE and RE‐LY Investigators . A biomarker‐based risk score to predict death in patients with atrial fibrillation: the ABC (age, biomarkers, clinical history) death risk score. Eur Heart J. 2018;39(6):477–85. 10.1093/eurheartj/ehx584 29069359 PMC5837352

[307] Esteve‐Pastor MA , Roldan V , Rivera‐Caravaca JM , Ramirez‐Macias I , Lip GYH , Marin F . The use of biomarkers in clinical management guidelines: a critical appraisal. Thromb Haemost. 2019 10.1055/s-0039-1696955 31499565

[308] Selvanayagam JB , Hartshorne T , Billot L , Grover S , Hillis GS , Jung W , et al. Cardiovascular magnetic resonance‐GUIDEd management of mild to moderate left ventricular systolic dysfunction (CMR GUIDE): study protocol for a randomized controlled trial. Ann Noninvasive Electrocardiol. 2017;22:e12420 10.1111/anec.12420 PMC6931571 28117536

[309] Chang TY , Lip GYH , Chen SA , Chao TF . Importance of risk reassessment in patients with atrial fibrillation in guidelines: assessing risk as a dynamic process. Can J Cardiol. 2019;35:611–8. 10.1016/j.cjca.2019.01.018 31030863

[310] Asunción Esteve‐Pastor M , Miguel Rivera‐Caravaca J , Roldan V , Vicente V , Valdes M , Marin F , et al. Long‐term bleeding risk prediction in 'real world' patients with atrial fibrillation: comparison of the HAS‐BLED and ABC‐Bleeding risk scores. The Murcia Atrial Fibrillation Project. Thromb Haemost. 2017;117:1848–58. 10.1160/TH17-07-0478 28799620

[311] Rivera‐Caravaca JM , Roldan V , Esteve‐Pastor MA , Valdes M , Vicente V , Lip GYH , et al. Long‐term stroke risk prediction in patients with atrial fibrillation: comparison of the ABC‐stroke and CHA_2_DS_2_‐VASc scores. J Am Heart Assoc. 2017;6(7):1–9. e006490. 10.1161/JAHA.117.006490 PMC5586327 28729407

[312] Kaarisalo MM , Immonen‐Raiha P , Marttila RJ , Salomaa V , Kaarsalo E , Salmi K , et al. Atrial fibrillation and stroke. Mortality and causes of death after the first acute ischemic stroke. Stroke. 1997;28:311–5. 10.1161/01.STR.28.2.311 9040681

[313] Gao H , Sun X , Li W , Gao Q , Zhang J , Zhang Y , et al. Development and validation of a risk score to predict 30‐day mortality in patients with atrial fibrillation‐related stroke: GPS‐GF score. Neurol Res. 2018;40:532–40. 10.1080/01616412.2018.1451431 29544401

[314] Reinier K , Marijon E , Uy‐Evanado A , Teodorescu C , Narayanan K , Chugh H , et al. The association between atrial fibrillation and sudden cardiac death: the relevance of heart failure. JACC Heart Fail. 2014;2:221–7. 10.1016/j.jchf.2013.12.006 24952687

[315] Okin PM , Bang CN , Wachtell K , Hille DA , Kjeldsen SE , Dahlof B , et al. Relationship of sudden cardiac death to new‐onset atrial fibrillation in hypertensive patients with left ventricular hypertrophy. Circ Arrhythm Electrophysiol. 2013;6:243–51. 10.1161/CIRCEP.112.977777 23403268

[316] Chen LY , Sotoodehnia N , Bůžková P , Lopez FL , Yee LM , Heckbert SR , et al. Atrial fibrillation and the risk of sudden cardiac death: the atherosclerosis risk in communities study and cardiovascular health study. JAMA Intern Med. 2013;173:29–35. 10.1001/2013.jamainternmed.744 23404043 PMC3578214

[317] Rattanawong P , Upala S , Riangwiwat T , Jaruvongvanich V , Sanguankeo A , Vutthikraivit W , et al. Atrial fibrillation is associated with sudden cardiac death: a systematic review and meta‐analysis. J Interv Card Electrophysiol. 2018;51:91–104. 10.1007/s10840-017-0308-9 29332241

[318] Chao TF , Liu CJ , Tuan TC , Chen SJ , Chen TJ , Lip GYH , et al. Risk and prediction of sudden cardiac death and ventricular arrhythmias for patients with atrial fibrillation—a Nationwide Cohort Study. Sci Rep. 2017;7:46445 10.1038/srep46445 28422144 PMC5396069

[319] Eisen A , Ruff CT , Braunwald E , Nordio F , Corbalan R , Dalby A , et al. Sudden cardiac death in patients with atrial fibrillation: insights from the ENGAGE AF‐TIMI 48 trial. J Am Heart Assoc. 2016;5(7):1–12. e003735. 10.1161/JAHA.116.003735 PMC5015407 27402235

[320] Roy D , Talajic M , Nattel S , Wyse DG , Dorian P , Lee KL , et al. Rhythm control versus rate control for atrial fibrillation and heart failure. N Engl J Med. 2008;358:2667–77. 10.1056/NEJMoa0708789 18565859

[321] Arbelo E , Brugada J , Hindricks G , Maggioni A , Tavazzi L , Vardas P , et al.;on behalf of the Atrial Fibrillation Ablation Pilot Study Investigators . ESC‐EURObservational Research Programme: the Atrial Fibrillation Ablation Pilot Study, conducted by the European Heart Rhythm Association. Europace. 2012;14:1094–103. 10.1093/europace/eus153 22628450

[322] Arbelo E , Brugada J , Hindricks G , Maggioni AP , Tavazzi L , Vardas P , et al.;on the behalf of the Atrial Fibrillation Ablation Pilot Study Investigators . The atrial fibrillation ablation pilot study: a European Survey on Methodology and results of catheter ablation for atrial fibrillation conducted by the European Heart Rhythm Association. Eur Heart J. 2014;35:1466–78. 10.1093/eurheartj/ehu001 24487524

[323] Arbelo E , Brugada J , Blomstrom‐Lundqvist C , Laroche C , Kautzner J , Pokushalov E , et al.;on the behalf of the ESC‐EHRA Atrial Fibrillation Ablation Long‐term Registry Investigators . Contemporary management of patients undergoing atrial fibrillation ablation: in‐hospital and 1‐year follow‐up findings from the ESC‐EHRA atrial fibrillation ablation long‐term registry. Eur Heart J. 2017;38:1303–16. 10.1093/eurheartj/ehw564 28104790

[324] De Greef Y , Stroker E , Schwagten B , Kupics K , De Cocker J , Chierchia GB , et al. Complications of pulmonary vein isolation in atrial fibrillation: predictors and comparison between four different ablation techniques: results from the MIddelheim PVI‐registry. Europace. 2018;20:1279–86. 10.1093/europace/eux233 29016870

[325] Steinbeck G , Sinner MF , Lutz M , Muller‐Nurasyid M , Kaab S , Reinecke H . Incidence of complications related to catheter ablation of atrial fibrillation and atrial flutter: a nationwide in‐hospital analysis of administrative data for Germany in 2014. Eur Heart J. 2018;39:4020–9. 10.1093/eurheartj/ehy452 30085086 PMC6269631

[326] Fink T , Metzner A , Willems S , Eckardt L , Ince H , Brachmann J , et al. Procedural success, safety and patients satisfaction after second ablation of atrial fibrillation in the elderly: results from the German Ablation Registry. Clin Res Cardiol. 2019;108:1354–63. 10.1007/s00392-019-01471-5 30953179

[327] Bunch TJ , May HT , Bair TL , Anderson JL , Crandall BG , Cutler MJ , et al. Long‐term natural history of adult Wolff‐Parkinson‐White syndrome patients treated with and without catheter ablation. Circ Arrhythm Electrophysiol. 2015;8:1465–71. 10.1161/CIRCEP.115.003013 26480930

[328] Haissaguerre M , Jaïs P , Shah DC , Takahashi A , Hocini M , Quiniou G , et al. Spontaneous initiation of atrial fibrillation by ectopic beats originating in the pulmonary veins. N Engl J Med. 1998;339:659–66. 10.1056/NEJM199809033391003 9725923

[329] Ebert M , Stegmann C , Kosiuk J , Dinov B , Richter S , Predictors AA , et al. and outcome of cardioversion failure early after atrial fibrillation ablation. Europace. 2018;20:1428–34. 10.1093/europace/eux327 29165582

[330] Deng H , Bai Y , Shantsila A , Fauchier L , Potpara TS , Lip GY . Clinical scores for outcomes of rhythm control or arrhythmia progression in patients with atrial fibrillation: a systematic review. Clin Res Cardiol. 2017;106:813–23. 10.1007/s00392-017-1123-0 28560516 PMC5613037

[331] Degiovanni A , Boggio E , Prenna E , Sartori C , De Vecchi F , Marino PN ,; et al. Association between left atrial phasic conduit function and early atrial fibrillation recurrence in patients undergoing electrical cardioversion. Clin Res Cardiol. 2018;107:329–37. 10.1007/s00392-017-1188-9 29181725 PMC5869942

[332] Mujović N , Marinković M , Lip GY , Potpara TS . Predicting recurrent atrial fibrillation after catheter ablation. Europace. 2018;20(FI_3):f460–1. 10.1093/europace/euy022 29554247

[333] Sepehri Shamloo A , Dagres N , Dinov B , Sommer P , Husser‐Bollmann D , Bollmann A , et al. Is epicardial fat tissue associated with atrial fibrillation recurrence after ablation? A systematic review and meta‐analysis. Int J Cardiol Heart Vasc. 2019;22: 132–8. 10.1016/j.ijcha.2019.01.003 30740509 PMC6356021

[334] Mesquita J , Ferreira AM , Cavaco D , Moscoso Costa F , Carmo P , Marques H , et al. Development and validation of a risk score for predicting atrial fibrillation recurrence after a first catheter ablation procedure—ATLAS score. Europace. 2018;20: f428–f435. 10.1093/europace/eux265 29016770

[335] Winkle RA , Jarman JWE , Mead RH , Engel G , Kong MH , Fleming W , et al. Predicting atrial fibrillation ablation outcome: the CAAP‐AF score. Heart Rhythm. 2016;13: 2119–25. 10.1016/j.hrthm.2016.07.018 27435586

[336] Canpolat U , Aytemir K , Yorgun H , Şahiner L , Kaya EB , Oto A . A proposal for a new scoring system in the prediction of catheter ablation outcomes: Promising results from the Turkish Cryoablation Registry. Int J Cardiol. 2013;169:201–6. 10.1016/j.ijcard.2013.08.097 24063932

[337] Oacute Jcik M , Berkowitsch A , Greiss H , Zaltsberg S , Pajitnev D , Deubner N , et al. Repeated catheter ablation of atrial fibrillation. Circ J. 2013;77: 2271–9. 10.1253/circj.CJ-13-0308 23759661

[338] Kornej J , Buttner P , Sommer P , Dagres N , Dinov B , Schumacher K , et al. Prediction of electro‐anatomical substrate using APPLE score and biomarkers. Europace. 2019;21:54–9. 10.1093/europace/euy120 29893827

[339] Buttner P , Schumacher K , Dinov B , Zeynalova S , Sommer P , Bollmann A , et al. Role of NT‐proANP and NT‐proBNP in patients with atrial fibrillation: association with atrial fibrillation progression phenotypes. Heart Rhythm. 2018;15:1132–7. 10.1016/j.hrthm.2018.03.021 29604419

[340] Kornej J , Hindricks G , Shoemaker MB , Husser D , Arya A , Sommer P , et al. The APPLE score: a novel and simple score for the prediction of rhythm outcomes after catheter ablation of atrial fibrillation. Clin Res Cardiol. 2015;104: 871–6. 10.1007/s00392-015-0856-x 25876528 PMC4726453

[341] Kornej J , Hindricks G , Arya A , Sommer P , Husser D , Bollmann A . The APPLE Score—a novel score for the prediction of rhythm outcomes after repeat catheter ablation of atrial fibrillation. PLoS ONE. 2017;12:e0169933 10.1371/journal.pone.0169933 28085921 PMC5235369

[342] Kornej J , Schumacher K , Dinov B , Kosich F , Sommer P , Arya A , et al. Prediction of electro‐anatomical substrate and arrhythmia recurrences using APPLE, DR‐FLASH and MB‐LATER scores in patients with atrial fibrillation undergoing catheter ablation. Sci Rep. 2018;8:12686 10.1038/s41598-018-31133-x 30139967 PMC6107514

[343] Kosiuk J , Dinov B , Kornej J , Acou WJ , Schonbauer R , Fiedler L , et al. Prospective, multicenter validation of a clinical risk score for left atrial arrhythmogenic substrate based on voltage analysis: DR‐FLASH score. Heart Rhythm. 2015;12:2207–12. 10.1016/j.hrthm.2015.07.003 26144350

[344] Cappato R , Calkins H , Chen SA , Davies W , Iesaka Y , Kalman J , et al. Updated worldwide survey on the methods, efficacy, and safety of catheter ablation for human atrial fibrillation. Circ Arrhythm Electrophysiol. 2010;3:32–8. 10.1161/CIRCEP.109.859116 19995881

[345] Lee G , Sparks PB , Morton JB , Kistler PM , Vohra JK , Medi C , et al. Low risk of major complications associated with pulmonary vein antral isolation for atrial fibrillation: results of 500 consecutive ablation procedures in patients with low prevalence of structural heart disease from a single center. J Cardiovasc Electrophysiol. 2011;22:163–8. 10.1111/j.1540-8167.2010.01870.x 20731742

[346] Deshmukh A , Patel NJ , Pant S , Shah N , Chothani A , Mehta K , et al. In‐hospital complications associated with catheter ablation of atrial fibrillation in the United States between 2000 and 2010: analysis of 93 801 procedures. Circulation. 2013;128:2104–12. 10.1161/CIRCULATIONAHA.113.003862 24061087

[347] Tripathi B , Arora S , Kumar V , Abdelrahman M , Lahewala S , Dave M , et al. Temporal trends of in‐hospital complications associated with catheter ablation of atrial fibrillation in the United States: an update from Nationwide Inpatient Sample database (2011–2014). J Cardiovasc Electrophysiol. 2018;29:715–24. 10.1111/jce.13471 29478273

[348] Voskoboinik A , Sparks PB , Morton JB , Lee G , Joseph SA , Hawson JJ , et al. Low rates of major complications for radiofrequency ablation of atrial fibrillation maintained over 14 years: a single centre experience of 2750 consecutive cases. Heart Lung Circ. 2018;27:976–83. 10.1016/j.hlc.2018.01.002 29523465

[349] Shamloo AS , Dagres N , Arya A , Hindricks G . Atrial fibrillation: a review of modifiable risk factors and preventive strategies. Rom J Intern Med. 2019;57:99–109. 10.2478/rjim-2018-0045 30648669

[350] Pathak RK , Middeldorp ME , Lau DH , Mehta AB , Mahajan R , Twomey D , et al. Aggressive risk factor reduction study for atrial fibrillation and implications for the outcome of ablation: the ARREST‐AF cohort study. J Am Coll Cardiol. 2014;64:2222–31. 10.1016/j.jacc.2014.09.028 25456757

[351] Providencia R , Adragao P , de Asmundis C , Chun J , Chierchia G , Defaye P , et al. Impact of body mass index on the outcomes of catheter ablation of atrial fibrillation: a European Observational Multicenter Study. J Am Heart Assoc. 2019;8:e012253. 31581876 10.1161/JAHA.119.012253 PMC6818047

[352] Voskoboinik A , Kalman JM , De Silva A , Nicholls T , Costello B , Nanayakkara S , et al. Alcohol abstinence in drinkers with atrial fibrillation. N Engl J Med. 2020;382:20–8. 10.1056/NEJMoa1817591 31893513

[353] Pappone C , Rosanio S , Augello G , Gallus G , Vicedomini G , Mazzone P , et al. Mortality, morbidity, and quality of life after circumferential pulmonary vein ablation for atrial fibrillation: outcomes from a controlled nonrandomized long‐term study. J Am Coll Cardiol. 2003;42:185–97. 10.1016/S0735-1097(03)00577-1 12875749

[354] Mansour M , Heist EK , Agarwal R , Bunch TJ , Karst E , Ruskin JN , et al. Stroke and cardiovascular events after ablation or antiarrhythmic drugs for treatment of patients with atrial fibrillation. Am J Cardiol. 2018;121:1192–9. 10.1016/j.amjcard.2018.01.043 29571722

[355] Themistoclakis S , Corrado A , Marchlinski FE , Jais P , Zado E , Rossillo A , et al. The risk of thromboembolism and need for oral anticoagulation after successful atrial fibrillation ablation. J Am Coll Cardiol. 2010;55:735–43. 10.1016/j.jacc.2009.11.039 20170810

[356] Cox JL , Ad N , Palazzo T , Fitzpatrick S , Suyderhoud JP , DeGroot KW , et al. Current status of the Maze procedure for the treatment of atrial fibrillation. Semin Thorac Cardiovasc Surg. 2000;12:15–9. 10.1016/S1043-0679(00)70011-6 10746917

[357] La Meir M , Gelsomino S , Luca F , Pison L , Colella A , Lorusso R , et al. Minimal invasive surgery for atrial fibrillation: an updated review. Europace. 2013;15:170–82. 10.1093/europace/eus216 22782971

[358] Gaynor SL , Diodato MD , Prasad SM , Ishii Y , Schuessler RB , Bailey MS , et al. A prospective, single‐center clinical trial of a modified Cox Maze procedure with bipolar radiofrequency ablation. J Thorac Cardiovasc Surg. 2004;128:535–42. 10.1016/j.jtcvs.2004.02.044 15457154

[359] Fumagalli S , Chen J , Dobreanu D , Madrid AH , Tilz R , Dagres N . The role of the Arrhythmia Team, an integrated, multidisciplinary approach to treatment of patients with cardiac arrhythmias: results of the European Heart Rhythm Association survey. Europace. 2016;18:623–7. 10.1093/europace/euw090 27174994

[360] Gammie JS , Haddad M , Milford‐Beland S , Welke KF , Ferguson TB Jr , O'Brien SM , et al. Atrial fibrillation correction surgery: lessons from the Society of Thoracic Surgeons National Cardiac Database. Ann Thorac Surg. 2008;85:909–14. 10.1016/j.athoracsur.2007.10.097 18291169

[361] Khurram IM , Maqbool F , Berger RD , Marine JE , Spragg DD , Ashikaga H , et al. Association between left atrial stiffness index and atrial fibrillation recurrence in patients undergoing left atrial ablation. Circ Arrhythm Electrophysiol. 2016;9(3):1–9. e003163 10.1161/CIRCEP.115.003163 26966287

[362] Chen MC , Chang JP , Chang HW . Preoperative atrial size predicts the success of radiofrequency Maze procedure for permanent atrial fibrillation in patients undergoing concomitant valvular surgery. Chest. 2004;125:2129–34. 10.1378/chest.125.6.2129 15189932

[363] Sunderland N , Maruthappu M , Nagendran M . What size of left atrium significantly impairs the success of Maze surgery for atrial fibrillation? Interact Cardiovasc Thorac Surg. 2011;13:332–8. 10.1510/icvts.2011.271999 21632865

[364] Chaiyaroj S , Ngarmukos T , Lertsithichai P . Predictors of sinus rhythm after radiofrequency Maze and mitral valve surgery. Asian Cardiovasc Thorac Ann. 2008;16:292–7. 10.1177/021849230801600407 18670021

[365] Gillinov AM , Bhavani S , Blackstone EH , Rajeswaran J , Svensson LG , Navia JL , et al. Surgery for permanent atrial fibrillation: impact of patient factors and lesion set. Ann Thorac Surg. 2006;82:502–13; discussion 13‐4. 10.1016/j.athoracsur.2006.02.030 16863753

[366] Beukema WP , Sie HT , Misier AR , Delnoy PP , Wellens HJ , Elvan A . Predictive factors of sustained sinus rhythm and recurrent atrial fibrillation after a radiofrequency modified Maze procedure. Eur J Cardio‐Thorac Surg. 2008;34:771–5. 10.1016/j.ejcts.2008.07.026 18768326

[367] Boersma LVA , Castella M , van Boven WimJan , Berruezo A , Yilmaz A , Nadal M , et al. Atrial fibrillation catheter ablation versus surgical ablation treatment (FAST): a 2‐center randomized clinical trial. Circulation. 2012;125:23–30. 10.1161/CIRCULATIONAHA.111.074047 22082673

[368] Damiano RJ Jr , Schwartz FH , Bailey MS , Maniar HS , Munfakh NA , Moon MR , et al. The Cox Maze IV procedure: predictors of late recurrence. J Thorac Cardiovasc Surg. 2011;141:113–21. 10.1016/j.jtcvs.2010.08.067 21168019 PMC3035158

[369] Edgerton JR , McClelland JH , Duke D , Gerdisch MW , Steinberg BM , Bronleewe SH , et al. Minimally invasive surgical ablation of atrial fibrillation: six‐month results. J Thorac Cardiovasc Surg. 2009;138:109–13; discussion 14. 10.1016/j.jtcvs.2008.09.080 19577065

[370] Myerburg RJ , Mitrani R , Interian A Jr , Castellanos A . Interpretation of outcomes of antiarrhythmic clinical trials: design features and population impact. Circulation. 1998;97:1514–21. 10.1161/01.CIR.97.15.1514 9576433

[371] Disertori M , Rigoni M , Pace N , Casolo G , Mase M , Gonzini L , et al. Myocardial fibrosis assessment by LGE is a powerful predictor of ventricular tachyarrhythmias in ischemic and nonischemic LV dysfunction: a meta‐analysis. JACC Cardiovasc Imaging. 2016;9:1046–55. 10.1016/j.jcmg.2016.01.033 27450871

[372] Connolly SJ , Hallstrom AP , Cappato R , Schron EB , Kuck KH , Zipes DP , et al. Meta‐analysis of the implantable cardioverter defibrillator secondary prevention trials. AVID, CASH and CIDS studies. Antiarrhythmics vs Implantable Defibrillator study. Cardiac Arrest Study Hamburg. Canadian Implantable Defibrillator Study. Eur Heart J. 2000;21:2071–8. 10.1053/euhj.2000.2476 11102258

[373] Disertori M , Mase M , Rigoni M , Nollo G , Ravelli F . heart rate turbulence is a powerful predictor of cardiac death and ventricular arrhythmias in postmyocardial infarction and heart failure patients: a systematic review and meta‐analysis. Circ Arrhythm Electrophysiol. 2016;9 10.1161/CIRCEP.116.004610 27879279

[374] Morin DP , Zacks ES , Mauer AC , Ageno S , Janik M , Markowitz SM , et al. Effect of bundle branch block on microvolt T‐wave alternans and electrophysiologic testing in patients with ischemic cardiomyopathy. Heart Rhythm. 2007;4:904–12. 10.1016/j.hrthm.2007.02.027 17599676

[375] Biering‐Sorensen T , Olsen FJ , Storm K , Fritz‐Hansen T , Olsen NT , Jons C , et al. Prognostic value of tissue Doppler imaging for predicting ventricular arrhythmias and cardiovascular mortality in ischaemic cardiomyopathy. Eur Heart J Cardiovasc Imaging. 2016;17:722–31. 10.1093/ehjci/jew066 27084397

[376] Bui AH , Cannon CP , Steg PG , Storey RF , Husted S , Guo J , et al. Relationship between early and late nonsustained ventricular tachycardia and cardiovascular death in patients with acute coronary syndrome in the Platelet Inhibition and Patient Outcomes (PLATO) Trial. Circ Arrhythm Electrophysiol. 2016;9:e002951 10.1161/CIRCEP.115.002951 26810596

[377] Prystowsky EN , Nisam S . Prophylactic implantable cardioverter defibrillator trials: MUSTT, MADIT, and beyond. Multicenter Unsustained Tachycardia Trial. Multicenter Automatic Defibrillator Implantation Trial. Am J Cardiol. 2000;86:1214–5. a5. 10.1016/S0002-9149(00)01205-4 11090794

[378] Goldberger JJ , Subacius H , Patel T , Cunnane R , Kadish AH . Sudden cardiac death risk stratification in patients with nonischemic dilated cardiomyopathy. J Am Coll Cardiol. 2014;63:1879–89. 10.1016/j.jacc.2013.12.021 24445228

[379] Desai AS , Fang JC , Maisel WH , Baughman KL . Implantable defibrillators for the prevention of mortality in patients with nonischemic cardiomyopathy: a meta‐analysis of randomized controlled trials. JAMA. 2004;292:2874–9. 10.1001/jama.292.23.2874 15598919

[380] Kuruvilla S , Adenaw N , Katwal AB , Lipinski MJ , Kramer CM , Salerno M . Late gadolinium enhancement on cardiac magnetic resonance predicts adverse cardiovascular outcomes in nonischemic cardiomyopathy: a systematic review and meta‐analysis. Circ Cardiovasc Imaging. 2014;7:250–8. 10.1161/CIRCIMAGING.113.001144 24363358 PMC4007583

[381] Marume K , Noguchi T , Tateishi E , Morita Y , Kamakura T , Ishibashi K , et al. Mortality and sudden cardiac death risk stratification using the noninvasive combination of wide QRS duration and late gadolinium enhancement in idiopathic dilated cardiomyopathy. Circ Arrhythm Electrophysiol. 2018;11:e006233 10.1161/CIRCEP.117.006233 29654132

[382] Tung R , Bauer B , Schelbert H , Lynch JP , Auerbach M , Gupta P , et al. Incidence of abnormal positron emission tomography in patients with unexplained cardiomyopathy and ventricular arrhythmias: The potential role of occult inflammation in arrhythmogenesis. Heart Rhythm. 2015;12(12):2488–98. 10.1016/j.hrthm.2015.08.014 26272522 PMC4656080

[383] Heymans S , Eriksson U , Lehtonen J , Cooper LT Jr . The quest for new approaches in myocarditis and inflammatory cardiomyopathy. J Am Coll Cardiol. 2016;68:2348–64. 10.1016/j.jacc.2016.09.937 27884253

[384] Betensky BP , Tschabrunn CM , Zado ES , Goldberg LR , Marchlinski FE , Garcia FC , et al. Long‐term follow‐up of patients with cardiac sarcoidosis and implantable cardioverter‐defibrillators. Heart Rhythm. 2012;9:884–91. 10.1016/j.hrthm.2012.02.010 22338670

[385] Smedema J‐P , Snoep G , van Kroonenburgh MPG , van Geuns R‐J , Dassen WRM , Gorgels APM , et al. Evaluation of the accuracy of gadolinium‐enhanced cardiovascular magnetic resonance in the diagnosis of cardiac sarcoidosis. J Am Coll Cardiol. 2005;45:1683–90. 10.1016/j.jacc.2005.01.047 15893188

[386] Patel MR , Cawley PJ , Heitner JF , Klem I , Parker MA , Jaroudi WA , et al. Detection of myocardial damage in patients with sarcoidosis. Circulation. 2009;120:1969–77. 10.1161/CIRCULATIONAHA.109.851352 19884472 PMC2778859

[387] Murtagh G , Laffin LJ , Beshai JF , Maffessanti F , Bonham CA , Patel AV , et al. Prognosis of myocardial damage in sarcoidosis patients with preserved left ventricular ejection fraction: risk stratification using cardiovascular magnetic resonance. Circ Cardiovasc Imaging. 2016;9:e003738 10.1161/CIRCIMAGING.115.003738 26763280 PMC4718184

[388] Aquaro GD , Perfetti M , Camastra G , Monti L , Dellegrottaglie S , Moro C , et al. Cardiac MR with late gadolinium enhancement in acute myocarditis with preserved systolic function: ITAMY study. J Am Coll Cardiol. 2017;70:1977–87. 10.1016/j.jacc.2017.08.044 29025554

[389] Grani C , Eichhorn C , Biere L , Murthy VL , Agarwal V , Kaneko K , et al. Prognostic value of cardiac magnetic resonance tissue characterization in risk stratifying patients with suspected myocarditis. J Am Coll Cardiol. 2017;70:1964–76. 10.1016/j.jacc.2017.08.050 29025553 PMC6506846

[390] Mahfoud F , Gartner B , Kindermann M , Ukena C , Gadomski K , Klingel K , et al. Virus serology in patients with suspected myocarditis: utility or futility? Eur Heart J. 2011;32:897–903. 10.1093/eurheartj/ehq493 21217143

[391] Ichinose A , Otani H , Oikawa M , Takase K , Saito H , Shimokawa H , et al. MRI of cardiac sarcoidosis: basal and subepicardial localization of myocardial lesions and their effect on left ventricular function. Am J Roentgenol. 2008;191:862–9. 10.2214/AJR.07.3089 18716120

[392] Van Hare GF , Javitz H , Carmelli D , Saul JP , Tanel RE , Fischbach PS , et al. Prospective assessment after pediatric cardiac ablation: demographics, medical profiles, and initial outcomes. J Cardiovasc Electrophysiol. 2004;15:759–70. 10.1046/j.1540-8167.2004.03645.x 15250858

[393] Khairy P , Van Hare GF , Balaji S , Berul CI , Cecchin F , Cohen MI , et al. PACES/HRS expert consensus statement on the recognition and management of arrhythmias in adult congenital heart disease: developed in partnership between the Pediatric and Congenital Electrophysiology Society (PACES) and the Heart Rhythm Society (HRS). Endorsed by the governing bodies of PACES, HRS, the American College of Cardiology (ACC), the American Heart Association (AHA), the European Heart Rhythm Association (EHRA), the Canadian Heart Rhythm Society (CHRS), and the International Society for Adult Congenital Heart Disease (ISACHD). Can J Cardiol. 2014;30:e1–63. 10.1016/j.cjca.2014.09.002 25262867

[394] Brugada J , Blom N , Sarquella‐Brugada G , Blomstrom‐Lundqvist C , Deanfield J , Janousek J , et al. Pharmacological and non‐pharmacological therapy for arrhythmias in the pediatric population: EHRA and AEPC‐Arrhythmia Working Group joint consensus statement. Europace. 2013;15:1337–82. 10.1093/europace/eut082 23851511

[395] Harrison DA , Harris L , Siu SC , MacLoghlin CJ , Connelly MS , Webb GD , et al. Sustained ventricular tachycardia in adult patients late after repair of tetralogy of Fallot. J Am Coll Cardiol. 1997;30:1368–73. 10.1016/S0735-1097(97)00316-1 9350941

[396] Knauth AL , Gauvreau K , Powell AJ , Landzberg MJ , Walsh EP , Lock JE , et al. Ventricular size and function assessed by cardiac MRI predict major adverse clinical outcomes late after tetralogy of Fallot repair. Heart. 2008;94:211–6. 10.1136/hrt.2006.104745 17135219

[397] Khairy P , Landzberg MJ , Gatzoulis MA , Lucron H , Lambert J , MarçOn FOIS , et al. Value of programmed ventricular stimulation after tetralogy of fallot repair: a multicenter study. Circulation. 2004;109:1994–2000. 10.1161/01.CIR.0000126495.11040.BD 15051640

[398] Hernandez‐Madrid A , Paul T , Abrams D , Aziz PF , Blom NA , Chen J , et al. Arrhythmias in congenital heart disease: a position paper of the European Heart Rhythm Association (EHRA), Association for European Paediatric and Congenital Cardiology (AEPC), and the European Society of Cardiology (ESC) Working Group on Grown‐up Congenital heart disease, endorsed by HRS, PACES, APHRS, and SOLAECE. Europace. 2018;20:1719–53. 10.1093/europace/eux380 29579186

[399] Vehmeijer JT , Koyak Z , Bokma JP , Budts W , Harris L , Mulder BJM , et al. Sudden cardiac death in adults with congenital heart disease: does QRS‐complex fragmentation discriminate in structurally abnormal hearts? Europace. 2018;20:f122–f128. 10.1093/europace/eux044 28402450

[400] Priori SG , Wilde AA , Horie M , Cho Y , Behr ER , Berul C , et al. HRS/EHRA/APHRS expert consensus statement on the diagnosis and management of patients with inherited primary arrhythmia syndromes: document endorsed by HRS, EHRA, and APHRS in May 2013 and by ACCF, AHA, PACES, and AEPC in June 2013. Heart Rhythm. 2013;10:1932–63. 10.1016/j.hrthm.2013.05.014 24011539

[401] Towbin JA , McKenna WJ , Abrams DJ , Ackerman MJ , Calkins H , Darrieux FCC , et al. HRS expert consensus statement on evaluation, risk stratification, and management of arrhythmogenic cardiomyopathy. Heart Rhythm. 2019 10.1016/j.hrthm.2019.09.019 31078652

[402] Roston TM , Yuchi Z , Kannankeril PJ , Hathaway J , Vinocur JM , Etheridge SP , et al. The clinical and genetic spectrum of catecholaminergic polymorphic ventricular tachycardia: findings from an international multicentre registry. Europace. 2018;20:541–7. 10.1093/europace/euw389 28158428 PMC6059141

[403] Schwartz PJ , Ackerman MJ . The long QT syndrome: a transatlantic clinical approach to diagnosis and therapy. Eur Heart J. 2013;34:3109–16. 10.1093/eurheartj/eht089 23509228

[404] Hayashi M , Denjoy I , Extramiana F , Maltret A , Buisson NR , Lupoglazoff JM , et al. Incidence and risk factors of arrhythmic events in catecholaminergic polymorphic ventricular tachycardia. Circulation. 2009;119:2426–34. 10.1161/CIRCULATIONAHA.108.829267 19398665

[405] Adler A , Rosso R , Chorin E , Havakuk O , Antzelevitch C , Viskin S . Risk stratification in Brugada syndrome: clinical characteristics, electrocardiographic parameters, and auxiliary testing. Heart Rhythm. 2016;13:299–310. 10.1016/j.hrthm.2015.08.038 26341603

[406] Sieira J , Conte G , Ciconte G , Chierchia GB , Casado‐Arroyo R , Baltogiannis G , et al. A score model to predict risk of events in patients with Brugada Syndrome. Eur Heart J. 2017;38:1756–63. 10.1093/eurheartj/ehx119 28379344

[407] Yamagata K , Horie M , Aiba T , Ogawa S , Aizawa Y , Ohe T , et al. Genotype‐phenotype correlation of SCN5A mutation for the clinical and electrocardiographic characteristics of probands with Brugada syndrome: a Japanese Multicenter Registry. Circulation. 2017;135:2255–70. 10.1161/CIRCULATIONAHA.117.027983 28341781

[408] O'Mahony C , Jichi F , Pavlou M , Monserrat L , Anastasakis A , Rapezzi C , et al. A novel clinical risk prediction model for sudden cardiac death in hypertrophic cardiomyopathy (HCM risk‐SCD). Eur Heart J. 2014;35:2010–20. 10.1093/eurheartj/eht439 24126876

[409] Priori SG , Reviewers D , Wilde AA , Horie M , Cho Y , Behr ER , et al. Executive summary: HRS/EHRA/APHRS expert consensus statement on the diagnosis and management of patients with inherited primary arrhythmia syndromes. Europace. 2013;15:1389–406. 10.1093/europace/eut272 23994779

[410] van Rijsingen IA , Arbustini E , Elliott PM , Mogensen J , Hermans‐van Ast JF , van der Kooi AJ et al. Risk factors for malignant ventricular arrhythmias in Lamin A/C mutation carriers a European cohort study. J Am Coll Cardiol. 2012;59:493–500. 10.1016/j.jacc.2011.08.078 22281253

[411] van Rijsingen IA , van der Zwaag PA , Groeneweg JA , Nannenberg EA , Jongbloed JD , Zwinderman AH , et al. Outcome in phospholamban R14del carriers: results of a large multicentre cohort study. Circ Cardiovasc Genet. 2014;7:455–65. 10.1161/CIRCGENETICS.113.000374 24909667

[412] Corrado D , Link MS , Calkins H . Arrhythmogenic right ventricular cardiomyopathy. N Engl J Med. 2017;376:61–72. 10.1056/NEJMra1509267 28052233

[413] Pedersen CT , Kay GN , Kalman J , Borggrefe M , Della‐Bella P , Dickfeld T , et al. EHRA/HRS/APHRS expert consensus on ventricular arrhythmias. Heart Rhythm. 2014;11:e166–e196. 10.1016/j.hrthm.2014.07.024 25179489

[414] Calkins H , Corrado D , Marcus F . Risk stratification in arrhythmogenic right ventricular cardiomyopathy. Circulation. 2017;136:2068–82. 10.1161/CIRCULATIONAHA.117.030792 29158215 PMC5777304

[415] Wang W , Orgeron G , Tichnell C , Murray B , Crosson J , Monfredi O , et al. Impact of exercise restriction on arrhythmic risk among patients with arrhythmogenic right ventricular cardiomyopathy. J Am Heart Assoc. 2018;7 10.1161/JAHA.118.008843 PMC6220537 29909402

[416] Sawant AC , Te Riele AS , Tichnell C , Murray B , Bhonsale A , Tandri H , et al. Safety of American Heart Association‐recommended minimum exercise for desmosomal mutation carriers. Heart Rhythm. 2016;13:199–207. 10.1016/j.hrthm.2015.08.035 26321091

[417] Philips B , Madhavan S , James C , Tichnell C , Murray B , Needleman M , et al. High prevalence of catecholamine‐facilitated focal ventricular tachycardia in patients with arrhythmogenic right ventricular dysplasia/cardiomyopathy. Circ Arrhythm Electrophysiol. 2013;6:160–6. 10.1161/CIRCEP.112.975441 23275260

[418] Pinto YM , Elliott PM , Arbustini E , Adler Y , Anastasakis A , Bohm M , et al. Proposal for a revised definition of dilated cardiomyopathy, hypokinetic non‐dilated cardiomyopathy, and its implications for clinical practice: a position statement of the ESC working group on myocardial and pericardial diseases. Eur Heart J. 2016;37:1850–8. 10.1093/eurheartj/ehv727 26792875

[419] Deac M , Alpendurada F , Fanaie F , Vimal R , Carpenter JP , Dawson A , et al. Prognostic value of cardiovascular magnetic resonance in patients with suspected arrhythmogenic right ventricular cardiomyopathy. Int J Cardiol. 2013;168:3514–21. 10.1016/j.ijcard.2013.04.208 23701935

[420] Marcus FI , McKenna WJ , Sherrill D , Basso C , Bauce B , Bluemke DA , et al. Diagnosis of arrhythmogenic right ventricular cardiomyopathy/dysplasia: proposed modification of the Task Force Criteria. Eur Heart J. 2010;31:806–14. 10.1093/eurheartj/ehq025 20172912 PMC2848326

[421] Denis A , Sacher F , Derval N , Martin R , Lim HS , Pambrun T , et al. Arrhythmogenic response to isoproterenol testing vs. exercise testing in arrhythmogenic right ventricular cardiomyopathy patients. Europace. 2018;20:f30–f36. 10.1093/europace/euy007 29401235

[422] Bhonsale A , Groeneweg JA , James CA , Dooijes D , Tichnell C , Jongbloed JD , et al. Impact of genotype on clinical course in arrhythmogenic right ventricular dysplasia/cardiomyopathy‐associated mutation carriers. Eur Heart J. 2015;36:847–55. 10.1093/eurheartj/ehu509 25616645

[423] Quarta G , Syrris P , Ashworth M , Jenkins S , Zuborne Alapi K , Morgan J , et al. Mutations in the Lamin A/C gene mimic arrhythmogenic right ventricular cardiomyopathy. Eur Heart J. 2012;33:1128–36. 10.1093/eurheartj/ehr451 22199124

[424] Gali WL , Sarabanda AV , Baggio JM , Silva EF , Gomes GG . Junqueira LF. Predictors of mortality and heart transplantation in patients with Chagas' cardiomyopathy and ventricular tachycardia treated with implantable cardioverter‐defibrillators. Europace. 2019;21:1070–8. 10.1093/europace/euz012 30820579

[425] Ezekowitz JA , Rowe BH , Dryden DM , Hooton N , Vandermeer B , Spooner C , et al. Systematic review: implantable cardioverter defibrillators for adults with left ventricular systolic dysfunction. Ann Intern Med. 2007;147:251–62. 10.7326/0003-4819-147-4-200708210-00007 17709759

[426] Kolodziejczak M , Andreotti F , Kowalewski M , Buffon A , Ciccone MM , Parati G , et al. Implantable cardioverter‐defibrillators for primary prevention in patients with ischemic or nonischemic cardiomyopathy: a systematic review and meta‐analysis. Ann Intern Med. 2017;167:103–11. 10.7326/M17-0120 28632280

[427] Lee DS , Green LD , Liu PP , Dorian P , Newman DM , Grant FC , et al. Effectiveness of implantable defibrillators for preventing arrhythmic events and death: a meta‐analysis. J Am Coll Cardiol. 2003;41:1573–82. 10.1016/S0735-1097(03)00253-5 12742300

[428] Daubert JP , Zareba W , Cannom DS , McNitt S , Rosero SZ , Wang P , et al. Inappropriate implantable cardioverter‐defibrillator shocks in MADIT II: frequency, mechanisms, predictors, and survival impact. J Am Coll Cardiol. 2008;51:1357–65. 10.1016/j.jacc.2007.09.073 18387436

[429] Koller MT , Schaer B , Wolbers M , Sticherling C , Bucher HC , Osswald S . Death without prior appropriate implantable cardioverter‐defibrillator therapy: a competing risk study. Circulation. 2008;117:1918–26. 10.1161/CIRCULATIONAHA.107.742155 18391108

[430] Poole JE , Johnson GW , Hellkamp AS , Anderson J , Callans DJ , Raitt MH , et al. Prognostic importance of defibrillator shocks in patients with heart failure. N Engl J Med. 2008;359:1009–17. 10.1056/NEJMoa071098 18768944 PMC2922510

[431] Blom LJ , Visser M , Christiaans I , Scholten MF , Bootsma M , van den Berg MP , et al. Incidence and predictors of implantable cardioverter‐defibrillator therapy and its complications in idiopathic ventricular fibrillation patients. Europace. 2019;21:1519–26. 10.1093/europace/euz151 31114860

[432] Milman A , Hochstadt A , Andorin A , Gourraud JB , Sacher F , Mabo P , et al. Time‐to‐first appropriate shock in patients implanted prophylactically with an implantable cardioverter‐defibrillator: data from the Survey on Arrhythmic Events in BRUgada Syndrome (SABRUS). Europace. 2019;21:796–802. 10.1093/europace/euy301 30590530

[433] Dougherty CM , Hunziker J . Predictors of implantable cardioverter defibrillator shocks during the first year. J Cardiovasc Nurs. 2009;24:21–8. quiz 9–30. 10.1097/01.JCN.0000317473.42801.a8 19114797 PMC2673010

[434] Konstantino Y , Shafat T , Novack V , Novack L , Amit G . Incidence of implantable cardioverter defibrillator therapy and mortality in primary and secondary prevention of sudden cardiac death. Isr Med Assoc J. 2015;17:760–3. 26897978

[435] Nagahara D , Fujito T , Mochizuki A , Shimoshige S , Hashimoto A , Miura T . Predictors of appropriate ICD therapy in Japanese patients with structural heart diseases: a major role of prior sustained ventricular tachycardia in secondary prevention. J Arrhythmia. 2018;34:527–35. 10.1002/joa3.12086 PMC6174420 30327698

[436] van Welsenes GH , van Rees JB , Borleffs CJ , Cannegieter SC , Bax JJ , van Erven L , et al. Long‐term follow‐up of primary and secondary prevention implantable cardioverter defibrillator patients. Europace. 2011;13:389–94. 10.1093/europace/euq494 21208947

[437] Zareba W , Moss AJ , Jackson Hall W , Wilber DJ , Ruskin JN , McNitt S , et al.; MADIT II Investigators . Clinical course and implantable cardioverter defibrillator therapy in postinfarction women with severe left ventricular dysfunction. J Cardiovasc Electrophysiol. 2005;16:1265–70. 10.1111/j.1540-8167.2005.00224.x 16403053

[438] Santangeli P , Pelargonio G , Dello Russo A , Casella M , Bisceglia C , Bartoletti S , et al. Gender differences in clinical outcome and primary prevention defibrillator benefit in patients with severe left ventricular dysfunction: a systematic review and meta‐analysis. Heart Rhythm. 2010;7:876–82. 10.1016/j.hrthm.2010.03.042 20380893

[439] MacFadden DR , Crystal E , Krahn AD , Mangat I , Healey JS , Dorian P , et al. Sex differences in implantable cardioverter‐defibrillator outcomes: findings from a prospective defibrillator database. Ann Intern Med. 2012;156:195–203. 10.7326/0003-4819-156-3-201202070-00007 22312139

[440] Seegers J , Conen D , Jung K , Bergau L , Dorenkamp M , Luthje L , et al. Sex difference in appropriate shocks but not mortality during long‐term follow‐up in patients with implantable cardioverter‐defibrillators. Europace. 2016;18:1194–202. 10.1093/europace/euv361 26622054 PMC4974631

[441] van der Heijden AC , Thijssen J , Borleffs CJ , van Rees JB , Hoke U , van der Velde ET , et al. Gender‐specific differences in clinical outcome of primary prevention implantable cardioverter defibrillator recipients. Heart. 2013;99:1244–9. 10.1136/heartjnl-2013-304013 23723448

[442] Wijers SC , van der Kolk BY , Tuinenburg AE , Doevendans PA , Vos MA , Meine M . Implementation of guidelines for implantable cardioverter‐defibrillator therapy in clinical practice: which patients do benefit? Neth Heart J. 2013;21:274–83. 10.1007/s12471-013-0407-x 23572330 PMC3661880

[443] Conen D , Arendacka B , Rover C , Bergau L , Munoz P , Wijers S , et al. gender differences in appropriate shocks and mortality among patients with primary prophylactic implantable cardioverter‐defibrillators: systematic review and meta‐analysis. PLoS ONE. 2016;11:e0162756 10.1371/journal.pone.0162756 27618617 PMC5019464

[444] Ghanbari H , Dalloul G , Hasan R , Daccarett M , Saba S , David S , et al. Effectiveness of implantable cardioverter‐defibrillators for the primary prevention of sudden cardiac death in women with advanced heart failure: a meta‐analysis of randomized controlled trials. Arch Intern Med. 2009;169:1500–6. 10.1001/archinternmed.2009.255 19752408

[445] Providencia R , Marijon E , Lambiase PD , Bouzeman A , Defaye P , Klug D , et al. Primary prevention implantable cardioverter defibrillator (ICD) therapy in women‐data from a Multicenter French Registry. J Am Heart Assoc. 2016;5(2):1–10. e002756. 10.1161/JAHA.115.002756 PMC4802475 26873687

[446] Borleffs CJ , van Rees JB , van Welsenes GH , van der Velde ET , van Erven L , Bax JJ , et al. Prognostic importance of atrial fibrillation in implantable cardioverter‐defibrillator patients. J Am Coll Cardiol. 2010;55:879–85. 10.1016/j.jacc.2009.09.053 20185038

[447] Darma A , Nedios S , Kosiuk J , Richter S , Doering M , Arya A , et al. Differences in predictors of implantable cardioverter‐defibrillator therapies in patients with ischaemic and non‐ischaemic cardiomyopathies. Europace. 2016;18:405–12. 10.1093/europace/euv138 26056190

[448] Gronefeld GC , Mauss O , Li YG , Klingenheben T . Hohnloser SH. Association between atrial fibrillation and appropriate implantable cardioverter defibrillator therapy: results from a prospective study. J Cardiovasc Electrophysiol. 2000;11:1208–14. 10.1046/j.1540-8167.2000.01208.x 11083241

[449] Rienstra M , Smit MD , Nieuwland W , Tan ES , Wiesfeld AC , Anthonio RL , et al. Persistent atrial fibrillation is associated with appropriate shocks and heart failure in patients with left ventricular dysfunction treated with an implantable cardioverter defibrillator. Am Heart J. 2007;153:120–6. 10.1016/j.ahj.2006.09.010 17174649

[450] Chang IC , Agamawi YM , Austin E , Adkisson WO , Roukoz H , von Wald LN , et al. Usefulness of atrial fibrillation as a marker for adverse cardiovascular outcomes in both primary and secondary prevention in patients with implantable cardioverter‐defibrillators. Am J Cardiol. 2016;118:1497–502. 10.1016/j.amjcard.2016.08.016 27649879

[451] van Rees JB , Borleffs CJ , de Bie MK , Stijnen T , van Erven L , Bax JJ , et al. Inappropriate implantable cardioverter‐defibrillator shocks: incidence, predictors, and impact on mortality. J Am Coll Cardiol. 2011;57:556–62. 10.1016/j.jacc.2010.06.059 21272746

[452] Yang JH , Byeon K , Yim HR , Park JW , Park SJ , Huh J , et al. Predictors and clinical impact of inappropriate implantable cardioverter‐defibrillator shocks in Korean patients. J Korean Med Sci. 2012;27:619–24. 10.3346/jkms.2012.27.6.619 22690092 PMC3369447

[453] Deyell MW , Qi A , Chakrabarti S , Yeung‐Lai‐Wah JA , Tung S , Khoo C , et al. Prognostic impact of inappropriate defibrillator shocks in a population cohort. Heart. 2013;99:1250–5. 10.1136/heartjnl-2012-303407 23468515

[454] Fernandez‐Cisnal A , Arce‐Leon A , Arana‐Rueda E , Rodriguez‐Manero M , Gonzalez‐Cambeiro C , Moreno‐Arribas J , et al. Analyses of inappropriate shocks in a Spanish ICD primary prevention population: predictors and prognoses. Int J Cardiol. 2015;195:188–94. 10.1016/j.ijcard.2015.05.146 26046421

[455] Mustafa U , Dherange P , Reddy R , DeVillier J , Chong J , Ihsan A , et al. Atrial fibrillation is associated with higher overall mortality in patients with implantable cardioverter‐defibrillator: a systematic review and meta‐analysis. J Am Heart Assoc. 2018;7:e010156 10.1161/JAHA.118.010156 30554547 PMC6404454

[456] Powell BD , Saxon LA , Boehmer JP , Day JD , Gilliam FR , Heidenreich PA , et al. Survival after shock therapy in implantable cardioverter‐defibrillator and cardiac resynchronization therapy‐defibrillator recipients according to rhythm shocked. The ALTITUDE survival by rhythm study. J Am Coll Cardiol. 2013;62:1674–9. 10.1016/j.jacc.2013.04.083 23810882

[457] Bergau L , Willems R , Sprenkeler DJ , Fischer TH , Flevari P , Hasenfuß G , et al. Differential multivariable risk prediction of appropriate shock versus competing mortality—a prospective cohort study to estimate benefits from ICD therapy. Int J Cardiol. 2018;272:102–7. 10.1016/j.ijcard.2018.06.103 29983251

[458] Lelakowski J , Piekarz J , Rydlewska A , Majewski J , Senderek T , Ząbek A , et al. Factors predisposing to ventricular tachyarrhythmia leading to appropriate ICD intervention in patients with coronary artery disease or non‐ischaemic dilated cardiomyopathy. Kardiologia Polska. 2012;70:1264–75. 23264245

[459] Weeke P , Johansen JB , Jorgensen OD , Nielsen JC , Moller M , Videbaek R , et al. Mortality and appropriate and inappropriate therapy in patients with ischaemic heart disease and implanted cardioverter‐defibrillators for primary prevention: data from the Danish ICD Register. Europace. 2013;15:1150–7. 10.1093/europace/eut017 23407630

[460] Schaer B , Kuhne M , Reichlin T , Osswald S . Sticherling C. Incidence of and predictors for appropriate implantable cardioverter‐defibrillator therapy in patients with a secondary preventive implantable cardioverter‐defibrillator indication. Europace. 2016;18:227–31. 10.1093/europace/euv188 26063686

[461] Lee AKY , Andrade J , Hawkins NM , Alexander G , Bennett MT , Chakrabarti S , et al. Outcomes of untreated frequent premature ventricular complexes with normal left ventricular function. Heart. 2019;105:1408–13. 10.1136/heartjnl-2019-314922 31142596

[462] Baman TS , Lange DC , Ilg KJ , Gupta SK , Liu TY , Alguire C , et al. Relationship between burden of premature ventricular complexes and left ventricular function. Heart Rhythm. 2010;7:865–9. 10.1016/j.hrthm.2010.03.036 20348027

[463] Duffee DF , Shen WK , Smith HC . Suppression of frequent premature ventricular contractions and improvement of left ventricular function in patients with presumed idiopathic dilated cardiomyopathy. Mayo Clin Proc. 1998;73:430–3. 10.1016/S0025-6196(11)63724-5 9581582

[464] Hasdemir C , Ulucan C , Yavuzgil O , Yuksel A , Kartal Y , Simsek E , et al. Tachycardia‐induced cardiomyopathy in patients with idiopathic ventricular arrhythmias: the incidence, clinical and electrophysiologic characteristics, and the predictors. J Cardiovasc Electrophysiol. 2011;22:663–8. 10.1111/j.1540-8167.2010.01986.x 21235667

[465] Huizar JF , Ellenbogen KA , Tan AY , Kaszala K . Induced cardiomyopathy: JACC state‐of‐the‐art review. J Am Coll Cardiol. 2019;73:2328–44. 10.1016/j.jacc.2019.02.045 31072578 PMC6538508

[466] Tsuji A , Nagashima M , Hasegawa S , Nagai N , Nishibata K , Goto M , et al. Long‐term follow‐up of idiopathic ventricular arrhythmias in otherwise normal children. Jpn Circ J. 1995;59:654–62. 10.1253/jcj.59.654 8558749

[467] Hasdemir C , Yuksel A , Camli D , Kartal Y , Simsek E , Musayev O , et al. Late gadolinium enhancement CMR in patients with tachycardia‐induced cardiomyopathy caused by idiopathic ventricular arrhythmias. Pacing Clin Electrophysiol. 2012;35:465–70. 10.1111/j.1540-8159.2011.03324.x 22303908

[468] Penela D , Martinez M , Fernandez‐Armenta J , Aguinaga L , Tercedor L , Ordonez A , et al. Influence of myocardial scar on the response to frequent premature ventricular complex ablation. Heart. 2018. 10.1136/heartjnl-2018-313452 30242139

[469] Bogun F , Crawford T , Chalfoun N , Kuhne M , Sarrazin JF , Wells D , et al. Relationship of frequent postinfarction premature ventricular complexes to the reentry circuit of scar‐related ventricular tachycardia. Heart Rhythm. 2008;5:367–74. 10.1016/j.hrthm.2007.11.026 18313593

[470] Ellis ER , Josephson ME . Heart failure and tachycardia‐induced cardiomyopathy. Curr Heart Fail Rep. 2013;10:296–306. 10.1007/s11897-013-0150-z 23963583

[471] Huizar J . Prospective assessment of premature ventricular contractions suppression in cardiomyopathy (PAPS) (NCT03228823). 2017 [cited 2020 Mar 18]. Available from https://clinicaltrials.gov/ct2/show/NCT03228823

[472] Dabbagh GS , Bogun F . Predictors and therapy of cardiomyopathy caused by frequent ventricular ectopy. Curr Cardiol Rep. 2017;19:80 10.1007/s11886-017-0887-1 28752278

[473] Macatangay C , Viles‐Gonzalez JF , Goldberger JJ . Role of cardiac imaging in evaluating risk for sudden cardiac death. *Cardiac Electrophysiol* . Cardiac Electrophysiol Clin. 2017;9:639–50. 10.1016/j.ccep.2017.08.001 29173407

[474] Al‐Khatib SM , Stevenson WG , Ackerman MJ , Bryant WJ , Callans DJ , Curtis AB , et al. 2017 AHA/ACC/HRS guideline for management of patients with ventricular arrhythmias and the prevention of sudden cardiac death: executive summary: a report of the American College of Cardiology/American Heart Association Task Force on Clinical Practice Guidelines and the Heart Rhythm Society. Heart Rhythm. 2018;15:e190–e252. 10.1016/j.hrthm.2017.10.035 29097320

[475] Proclemer A , Bongiorni MG , Dagres N , Sciaraffia E , Todd D . Blomstrom‐Lundqvist C. How are European patients at risk of malignant arrhythmias or sudden cardiac death identified and informed about their risk profile: results of the European Heart Rhythm Association survey. Europace. 2015;17:994–8. 10.1093/europace/euv203 26023178

[476] Deyell MW , Krahn AD , Goldberger JJ . Sudden cardiac death risk stratification. Circ Res. 2015;116:1907–18. 10.1161/CIRCRESAHA.116.304493 26044247 PMC4466101

[477] Fallavollita JA , Dare JD , Carter RL , Baldwa S , Canty JM Jr . Denervated myocardium is preferentially associated with sudden cardiac arrest in ischemic cardiomyopathy: a pilot competing risks analysis of cause‐specific mortality. Circ Cardiovasc Imaging. 2017;10(8):1–11. e006446. 10.1161/CIRCIMAGING.117.006446 PMC5555076 28794139

[478] Deng D , Arevalo HJ , Prakosa A , Callans DJ , Trayanova NA . A feasibility study of arrhythmia risk prediction in patients with myocardial infarction and preserved ejection fraction. Europace. 2016;18:iv60–iv66. 10.1093/europace/euw351 28011832 PMC5225965

[479] Brugada P , Talajic M , Smeets J , Mulleneers R . Wellens HJ. The value of the clinical history to assess prognosis of patients with ventricular tachycardia or ventricular fibrillation after myocardial infarction. Eur Heart J. 1989;10:747–52. 10.1093/oxfordjournals.eurheartj.a059559 2792116

[480] Sarter BH , Finkle JK , Gerszten RE , Buxton AE . What is the risk of sudden cardiac death in patients presenting with hemodynamically stable sustained ventricular tachycardia after myocardial infarction? J Am Coll Cardiol. 1996;28:122–9. 10.1016/0735-1097(96)00123-4 8752804

[481] Al‐Khatib SM , Stevenson WG , Ackerman MJ , Bryant WJ , Callans DJ , Curtis AB , et al. AHA/ACC/HRS guideline for management of patients with ventricular arrhythmias and the prevention of sudden cardiac death. Circulation. 2017;2018(138):e272–e391. 10.1161/CIR.0000000000000549 29084731

[482] Friedman DJ , Al‐Khatib SM , Zeitler EP , Han J , Bardy GH , Poole JE , et al. New York Heart Association class and the survival benefit from primary prevention implantable cardioverter defibrillators: a pooled analysis of 4 randomized controlled trials. Am Heart J. 2017;191:21–9. 10.1016/j.ahj.2017.06.002 28888266 PMC5657554

[483] Tung R , Vaseghi M , Frankel DS , Vergara P , Di Biase L , Nagashima K , et al. Freedom from recurrent ventricular tachycardia after catheter ablation is associated with improved survival in patients with structural heart disease: an International VT Ablation Center Collaborative Group Study. Heart Rhythm. 2015;12:1997–2007. 10.1016/j.hrthm.2015.05.036 26031376 PMC4549209

[484] Bilchick KC , Wang Y , Cheng A , Curtis JP , Dharmarajan K , Stukenborg GJ , et al. Seattle heart failure and proportional risk models predict benefit from implantable cardioverter‐defibrillators. J Am Coll Cardiol. 2017;69:2606–18. 10.1016/j.jacc.2017.03.568 28545633 PMC5502749

[485] Kumar S , Androulakis AF , Sellal JM , Maury P , Gandjbakhch E , Waintraub X , et al. Multicenter experience with catheter ablation for ventricular tachycardia in Lamin A/C cardiomyopathy. Circ Arrhythm Electrophysiol. 2016;9(8):1–11. e004357. 10.1161/CIRCEP.116.004357 27506821

[486] Levy WC , Mozaffarian D , Linker DT , Sutradhar SC , Anker SD , Cropp AB , et al. The Seattle Heart Failure Model: prediction of survival in heart failure. Circulation. 2006;113:1424–33. 10.1161/CIRCULATIONAHA.105.584102 16534009

[487] Santangeli P , Muser D , Zado ES , Magnani S , Khetpal S , Hutchinson MD , et al. Acute hemodynamic decompensation during catheter ablation of scar‐related ventricular tachycardia: incidence, predictors, and impact on mortality. Circ Arrhythm Electrophysiol. 2015;8:68–75. 10.1161/CIRCEP.114.002155 25491601

[488] Vakil KP , Roukoz H , Tung R , Levy WC , Anand IS , Shivkumar K , et al. Mortality prediction using a modified Seattle Heart Failure Model may improve patient selection for ventricular tachycardia ablation. Am Heart J. 2015;170:1099–104. 10.1016/j.ahj.2015.09.008 26678631

[489] Santangeli P , Frankel DS , Tung R , Vaseghi M , Sauer WH , Early TWS , et al. mortality after catheter ablation of ventricular tachycardia in patients with structural heart disease. J Am Coll Cardiol. 2017;69:2105–15. 10.1016/j.jacc.2017.02.044 28449770

[490] Frankel DS , Tung R , Santangeli P , Tzou WS , Vaseghi M , Di Biase L , et al. Sex and catheter ablation for ventricular tachycardia: an International Ventricular Tachycardia Ablation. Center Collaborative Group Study. JAMA Cardiol. 2016;1:938–44. 10.1001/jamacardio.2016.2361 27556589

[491] Tzou WS , Tung R , Frankel DS , Vaseghi M , Bunch TJ , Di Biase L , et al. Ventricular tachycardia ablation in severe heart failure: an International Ventricular Tachycardia Ablation Center Collaboration Analysis. Circ Arrhythm Electrophysiol. 2017;10 10.1161/CIRCEP.116.004494 28082527

[492] Vaseghi M , Hu TY , Tung R , Vergara P , Frankel DS , Di Biase L , et al. Outcomes of catheter ablation of ventricular tachycardia based on etiology in nonischemic heart disease: an International Ventricular Tachycardia Ablation Center Collaborative study. JACC Clin Electrophysiol. 2018;4:1141–50. 10.1016/j.jacep.2018.05.007 30236386 PMC6242273

[493] Briceno DF , Romero J , Villablanca PA , Londono A , Diaz JC , Maraj I , et al. Long‐term outcomes of different ablation strategies for ventricular tachycardia in patients with structural heart disease: systematic review and meta‐analysis. Europace. 2018;20:104–15. 10.1093/europace/eux109 28575378 10.1093/europace/eux109

[494] Papageorgiou N , Providencia R , Bronis K , Dechering DG , Srinivasan N , Eckardt L , et al. Catheter ablation for ventricular tachycardia in patients with cardiac sarcoidosis: a systematic review. Europace. 2018;20:682–91. 10.1093/europace/eux077 28444174

[495] Avila P , Perez‐David E , Izquierdo M , Rojas‐Gonzalez A , Sanchez‐Gomez JM , Ledesma‐Carbayo MJ , et al. Scar extension measured by magnetic resonance‐based signal intensity mapping predicts ventricular tachycardia recurrence after substrate ablation in patients with previous myocardial infarction. JACC Clin Electrophysiol. 2015;1:353–65. 10.1016/j.jacep.2015.07.006 29759462

[496] Berruezo A , Acosta J , Fernandez‐Armenta J , Pedrote A , Barrera A , Arana‐Rueda E , et al. Safety, long‐term outcomes and predictors of recurrence after first‐line combined endoepicardial ventricular tachycardia substrate ablation in arrhythmogenic cardiomyopathy. Impact of arrhythmic substrate distribution pattern. A prospective multicentre study. Europace. 2017;19:607–16. 28431051 10.1093/europace/euw212

[497] Santangeli P , Zado ES , Supple GE , Haqqani HM , Garcia FC , Tschabrunn CM , et al. Long‐term outcome with catheter ablation of ventricular tachycardia in patients with arrhythmogenic right ventricular cardiomyopathy. Circ Arrhythm Electrophysiol. 2015;8:1413–21. 10.1161/CIRCEP.115.003562 26546346

[498] Dalal D , Jain R , Tandri H , Dong J , Eid SM , Prakasa K , et al. Long‐term efficacy of catheter ablation of ventricular tachycardia in patients with arrhythmogenic right ventricular dysplasia/cardiomyopathy. J Am Coll Cardiol. 2007;50:432–40. 10.1016/j.jacc.2007.03.049 17662396

[499] Fernandez‐Armenta J , Andreu D , Penela D , Trucco E , Cipolletta L , Arbelo E , et al. Sinus rhythm detection of conducting channels and ventricular tachycardia isthmus in arrhythmogenic right ventricular cardiomyopathy. Heart Rhythm. 2014;11:747–54. 10.1016/j.hrthm.2014.02.016 24561159

[500] Oloriz T , Silberbauer J , Maccabelli G , Mizuno H , Baratto F , Kirubakaran S , et al. Catheter ablation of ventricular arrhythmia in nonischemic cardiomyopathy: anteroseptal versus inferolateral scar sub‐types. Circ Arrhythm Electrophysiol. 2014;7:414–23. 10.1161/CIRCEP.114.001568 24785410

[501] Ghanbari H , Baser K , Yokokawa M , Stevenson W , Della Bella P , Vergara P , et al. Noninducibility in postinfarction ventricular tachycardia as an end point for ventricular tachycardia ablation and its effects on outcomes: a meta‐analysis. Circ Arrhythm Electrophysiol. 2014;7:677–83. 10.1161/CIRCEP.113.001404 24879789

[502] Yokokawa M , Kim HM , Baser K , Stevenson W , Nagashima K , Della Bella P , et al. Predictive value of programmed ventricular stimulation after catheter ablation of post‐infarction ventricular tachycardia. J Am Coll Cardiol. 2015;65:1954–9. 10.1016/j.jacc.2015.02.058 25913000

[503] Frankel DS , Mountantonakis SE , Zado ES , Anter E , Bala R , Cooper JM , et al. Noninvasive programmed ventricular stimulation early after ventricular tachycardia ablation to predict risk of late recurrence. J Am Coll Cardiol. 2012;59:1529–35. 10.1016/j.jacc.2012.01.026 22516442

[504] Oloriz T , Baratto F , Trevisi N , Barbaro M , Bisceglia C , D’Angelo G , et al. Defining the outcome of ventricular tachycardia ablation: timing and value of programmed ventricular stimulation. Circ Arrhythm Electrophysiol. 2018;11:e005602 10.1161/CIRCEP.117.005602 29545359

[505] Reddy VY , Reynolds MR , Neuzil P , Richardson AW , Taborsky M , Jongnarangsin K , et al. Prophylactic catheter ablation for the prevention of defibrillator therapy. N Engl J Med. 2007;357:2657–65. 10.1056/NEJMoa065457 18160685 PMC2390777

[506] Kuck KH , Schaumann A , Eckardt L , Willems S , Ventura R , Delacretaz E , et al. Catheter ablation of stable ventricular tachycardia before defibrillator implantation in patients with coronary heart disease (VTACH): a multicentre randomised controlled trial. Lancet. 2010;375:31–40. 10.1016/S0140-6736(09)61755-4 20109864

[507] Tilz RR , Kuck K‐H , Kääb S , Wegscheider K , Thiem A , Wenzel B , et al. Rationale and design of BERLIN VT study: a multicenter randomised trial comparing preventive versus deferred ablation of ventricular tachycardia. BMJ Open. 2019;9:e022910 10.1136/bmjopen-2018-022910 PMC6528000 31072848

[508] Kuck K‐H , Tilz RR , Deneke T , Hoffmann BA , Ventura R , Hansen PS , et al. Impact of substrate modification by catheter ablation on implantable cardioverter‐defibrillator interventions in patients with unstable ventricular arrhythmias and coronary artery disease: results from the multicenter randomized controlled SMS (Substrate Modification Study). Circ Arrhythm Electrophysiol. 2017;10 10.1161/CIRCEP.116.004422 28292751

[509] Dukes JW , Dewland TA , Vittinghoff E , Mandyam MC , Heckbert SR , Siscovick DS , et al. Ventricular ectopy as a predictor of heart failure and death. J Am Coll Cardiol. 2015;66:101–9. 10.1016/j.jacc.2015.04.062 26160626 PMC4499114

[510] Yokokawa M , Good E , Crawford T , Chugh A , Pelosi F Jr , Latchamsetty R , et al. Recovery from left ventricular dysfunction after ablation of frequent premature ventricular complexes. Heart Rhythm. 2013;10:172–5. 10.1016/j.hrthm.2012.10.011 23099051

[511] Carballeira Pol L , Deyell MW , Frankel DS , Benhayon D , Squara F , Chik W , et al. Ventricular premature depolarization QRS duration as a new marker of risk for the development of ventricular premature depolarization‐induced cardiomyopathy. Heart Rhythm. 2014;11:299–306. 10.1016/j.hrthm.2013.10.055 24184787

[512] Niwano S , Wakisaka Y , Niwano H , Fukaya H , Kurokawa S , Kiryu M , et al. Prognostic significance of frequent premature ventricular contractions originating from the ventricular outflow tract in patients with normal left ventricular function. Heart. 2009;95:1230–7. 10.1136/hrt.2008.159558 19429571

[513] Lin C‐Y , Chang S‐L , Lin Y‐J , Lo L‐W , Chung F‐P , Chen Y‐Y , et al. Long‐term outcome of multiform premature ventricular complexes in structurally normal heart. Int J Cardiol. 2015;180:80–5. 10.1016/j.ijcard.2014.11.110 25438221

[514] Del carpio munoz F , Syed FF , Noheria A , Cha Y‐M , Friedman PA , Hammill SC , et al. Characteristics of premature ventricular complexes as correlates of reduced left ventricular systolic function: study of the burden, duration, coupling interval, morphology and site of origin of PVCs. J Cardiovasc Electrophysiol. 2011;22:791–8. 10.1111/j.1540-8167.2011.02021.x 21332870

[515] Zhong LI , Lee Y‐H , Huang X‐M , Asirvatham SJ , Shen W‐K , Friedman PA , et al. Relative efficacy of catheter ablation vs antiarrhythmic drugs in treating premature ventricular contractions: a single‐center retrospective study. Heart Rhythm. 2014;11:187–93. 10.1016/j.hrthm.2013.10.033 24157533

[516] Olgun H , Yokokawa M , Baman T , Kim HM , Armstrong W , Good E , et al. The role of interpolation in PVC‐induced cardiomyopathy. Heart Rhythm. 2011;8:1046–9. 10.1016/j.hrthm.2011.02.034 21376837

[517] Leenhardt A , Glaser E , Burguera M , Nurnberg M , Maison‐Blanche P , Coumel P . Short‐coupled variant of torsade de pointes. A new electrocardiographic entity in the spectrum of idiopathic ventricular tachyarrhythmias. Circulation. 1994;89:206–15. 10.1161/01.CIR.89.1.206 8281648

[518] Prystowsky EN , Padanilam BJ , Joshi S , Fogel RI . Ventricular arrhythmias in the absence of structural heart disease. J Am Coll Cardiol. 2012;59:1733–44. 10.1016/j.jacc.2012.01.036 22575310

[519] Pappone C , Vicedomini G , Manguso F , Saviano M , Baldi M , Pappone A , et al. Wolff‐Parkinson‐White syndrome in the era of catheter ablation: insights from a registry study of 2169 patients. Circulation. 2014;130:811–9. 10.1161/CIRCULATIONAHA.114.011154 25052405

[520] Timmermans C , Smeets JL , Rodriguez L‐M , Vrouchos G , van den Dool A , Wellens HJJ . Aborted sudden death in the Wolff‐Parkinson‐White syndrome. Am J Cardiol. 1995;76:492–4. 10.1016/S0002-9149(99)80136-2 7653450

[521] Katritsis DG , Boriani G , Cosio FG , Hindricks G , Jais P , European JME , et al. Heart Rhythm Association (EHRA) consensus document on the management of supraventricular arrhythmias, endorsed by Heart Rhythm Society (HRS), Asia‐Pacific Heart Rhythm Society (APHRS), and Sociedad Latinoamericana de Estimulación Cardiaca y Electrofisiologia (SOLAECE). Europace. 2017;19:465–511. 10.1093/europace/euw301 27856540

[522] Katritsis DG , Boriani G , Cosio FG , Hindricks G , Jais P , European JME , et al. Heart Rhythm Association (EHRA) consensus document on the management of supraventricular arrhythmias, endorsed by Heart Rhythm Society (HRS), Asia‐Pacific Heart Rhythm Society (APHRS), and Sociedad Latinoamericana de Estimulacion Cardiaca y Electrofisiologia (SOLAECE). Eur Heart J. 2018;39:1442–5. 10.1093/eurheartj/ehw455 27856499

[523] Wackel P , Irving C , Webber S , Beerman L , Arora G . Risk stratification in Wolff‐Parkinson‐White syndrome: the correlation between noninvasive and invasive testing in pediatric patients. Pacing Clin Electrophysiol. 2012;35: 1451–7. 10.1111/j.1540-8159.2012.03518.x 22978820

[524] Spar DS , Silver ES , Hordof AJ , Liberman L . Relation of the utility of exercise testing for risk assessment in pediatric patients with ventricular preexcitation to pathway location. Am J Cardiol. 2012;109:1011–4. 10.1016/j.amjcard.2011.11.030 22221954

[525] Brugada J , Katritsis DG , Arbelo E , Arribas F , Bax JJ Blomstrom‐Lundqvist C , et al. 2019 ESC Guidelines for the management of patients with supraventricular tachycardiaThe Task Force for the management of patients with supraventricular tachycardia of the European Society of Cardiology (ESC). Eur Heart J. 2020;41(5):655–720. 31504425 10.1093/eurheartj/ehz467

[526] Medi C , Kalman JM , Haqqani H , Vohra JK , Morton JB , Sparks PB , et al. Tachycardia‐mediated cardiomyopathy secondary to focal atrial tachycardia: long‐term outcome after catheter ablation. J Am Coll Cardiol. 2009;53:1791–7. 10.1016/j.jacc.2009.02.014 19422986

[527] Arnar DO , Mairesse GH , Boriani G , Calkins H , Chin A , Coats A , et al. Management of asymptomatic arrhythmias: a European Heart Rhythm Association (EHRA) consensus document, endorsed by the Heart Failure Association (HFA), Heart Rhythm Society (HRS), Asia Pacific Heart Rhythm Society (APHRS), Cardiac Arrhythmia Society of Southern Africa (CASSA), and Latin America Heart Rhythm Society (LAHRS). Europace. 2019 10.1093/europace/euz046. [Epub ahead of print]. 30882141

